# Anhydrous and Stereoretentive Fluoride-Enhanced Suzuki–Miyaura Coupling of Immunomodulatory Imide Drug Derivatives

**DOI:** 10.1021/acs.joc.3c02873

**Published:** 2024-03-07

**Authors:** William F. Tracy, Geraint H. M. Davies, Lauren N. Grant, Jacob M. Ganley, Jesus Moreno, Emily C. Cherney, Huw M. L. Davies

**Affiliations:** † Department of Chemistry, Emory University, Atlanta, Georgia 30322, United States; ‡ Small Molecule Drug Discovery, Bristol Myers Squibb, Cambridge, Massachusetts 02140, United States; § Chemical Process Development, Bristol Myers Squibb, New Brunswick, New Jersey 08903, United States; ∥ Small Molecule Drug Discovery, Bristol Myers Squibb, San Diego, California 92121, United States; ⊥ Small Molecule Drug Discovery, Bristol Myers Squibb, Princeton, New Jersey 08543, United States

## Abstract

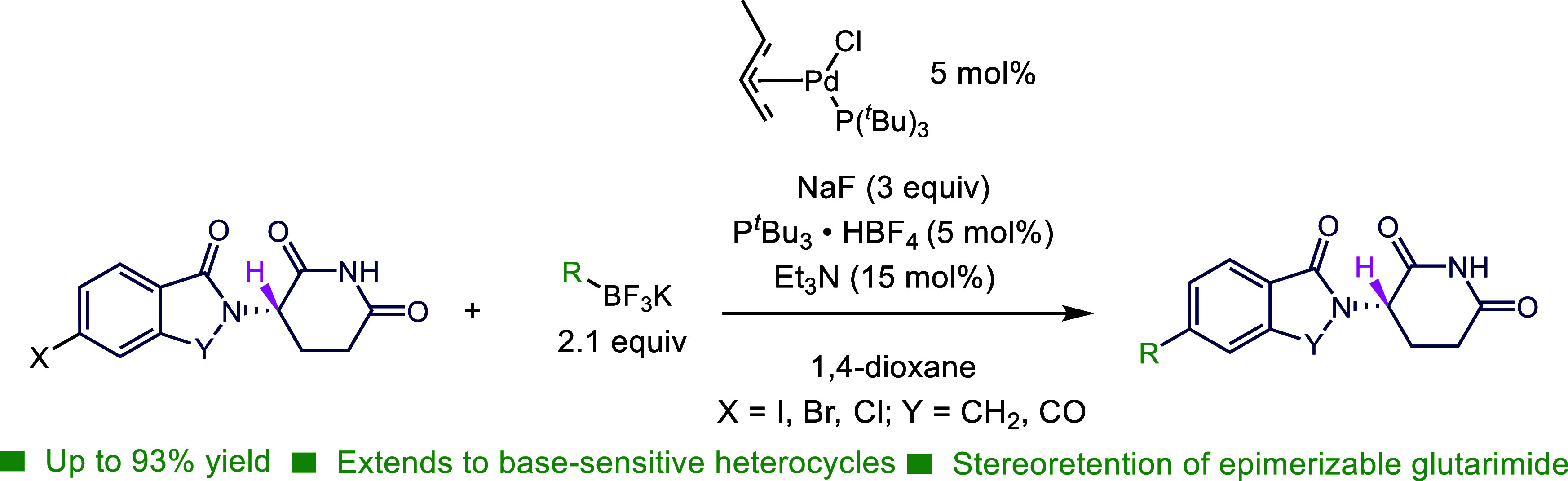

Immunomodulatory imide drugs form the core of many pharmaceutically relevant structures, but C_sp_^2^–C_sp_^2^ bond formation via metal-catalyzed cross coupling is difficult due to the sensitivity of the glutarimide ring ubiquitous in these structures. We report that replacement of the traditional alkali base with a fluoride source enhances a previously challenging Suzuki–Miyaura coupling on glutarimide-containing compounds with trifluoroborates. These enabling conditions are reactive enough to generate these derivatives in high yields but mild enough to preserve both the glutarimide and its sensitive stereocenter. Experimental and computational data suggest a mechanistically distinct process of π-coordination of the trifluoroborate enabled by these conditions.

## Introduction

Our understanding of glutarimide-containing drugs, such as thalidomide, has evolved over the past several decades to reveal a discrete mechanism that can be exploited for the targeted degradation of a wide variety of proteins of interest.^1a−1d^ As a result, glutarimides have proven to be exceptionally valuable to both scientists and patients, leading to the discovery of several marketed drugs.^2,3^ The key pharmacophore of these drugs, commonly referred to as immunomodulatory imide drugs (IMiDs) or Cereblon E3 Ligase Modulators (CELMoDs), is the glutarimide ring. While Cereblon was identified as the target binder for these molecules several years ago,^4,5^ the discovery that the glutarimide serves as a mimic for C-terminal degrons derived from the cyclization of asparagine or glutamine was only recently disclosed.^6,7^ Deep structural and mechanistic understanding, coupled with the potential for glutarimides to efficiently and selectively degrade previously undruggable targets of interest with event-driven pharmacology, has only intensified interest.^1d,8^ As such, there is high demand to create new glutarimide derivatives that can potentially impact human health.

**Figure 1:**
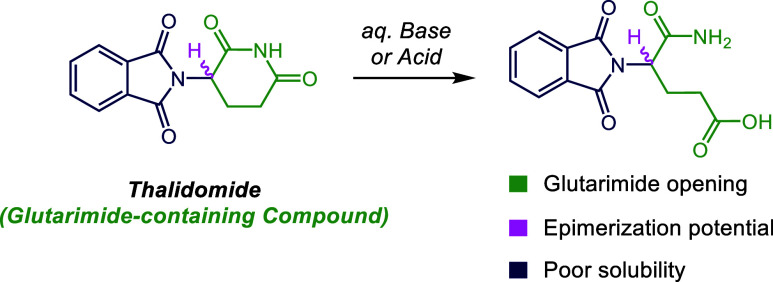
Synthetic challenges associated with glutarimide-containing compounds.

While a variety of methods have been used to generate IMiD derivatives,^9a−9h^ the glutarimide moiety presents synthetic challenges, stemming from poor solubility and propensity to open under a variety of basic or aqueous conditions (Figure 1).^10a−10c^ An additional complication is that the stereocenter adjacent to the imide is susceptible to epimerization under basic and acidic conditions.^11a−11d^ As a result, newly reported synthetic methods often highlight glutarimide compatibility.^12,13^ In our efforts to generate vinyl IMiD derivatives, we found a dearth of conditions in the academic literature for this particular kind of coupling in the presence of a glutarimide, despite the fact that vinylation of aryl halides is a well-studied reaction class.^14^ This can be explained, at least in part, by aforementioned sensitivity of the glutarimide to the traditional aqueous alkali conditions employed for Suzuki–Miyaura couplings (SMCs).^9g^ Base sensitivity in SMCs has been addressed outside the realm of glutarimides by Denmark,^15^ Niwa,^16^ and others. These methods, while providing elegant solutions, rely on less- or noncommercially available stoichiometric reagents (e.g., neopentyl boronates, in the case of Denmark’s work, and the zinc-based Lewis acid used in Niwa’s), or are limited to a small subset of useable solvents.^15−17^

Herein, we describe a method that enables the SMC of potassium trifluoroborates to glutarimide derivatives under mild and anhydrous conditions.^18^ Our studies of the reaction instigated a computational study, which helped explain the significant role of inorganic fluoride on the reaction and why vinyl trifluoroborates are particularly favored substrates.

## Results and Discussion

**Scheme 1:**
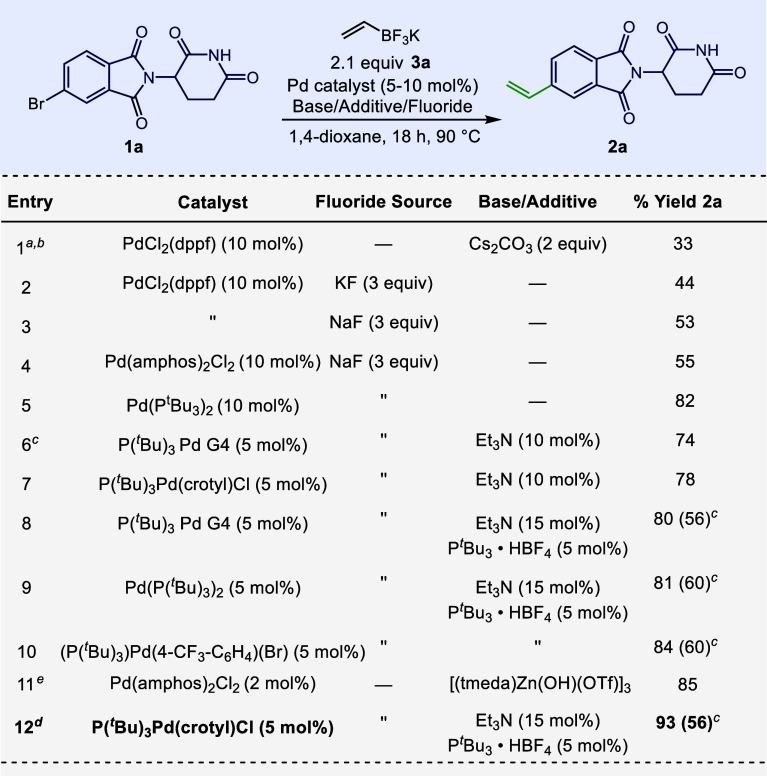
Optimization of Reaction Conditions Isolated yields reported. ^*a*^3.0 equic **3a** used. ^*b*^mol % catalyst used. ^*c*^Isolated reaction yield without added fluoride. ^*d*^A reaction run in PTFE-lined vesse gave **2a** in 78% yield. ^*e*^Reaction conducted using 2 mol % catalyst, 2.34 equiv [(tmeda)Zn(OH)(OTf)]_3_, 1.1 equiv **3a**, in THF.

In our own approach to generating glutarimide derivative **2a**, alternative routes (e.g., semireduction of the alkyne or formylation followed by Wittig olefination) were found ineffective. SMC conditions previously developed for use on IMiD-class structures failed to give an appreciable amount of **2a**,^9c^ as did more general conditions for the vinylation of aryl halides.^17,19^ Conditions from the patent literature demonstrating a Suzuki-type vinylation of glutarimides in the presence of cesium carbonate (Scheme 1, entry 1)^20^ gave a 33% yield of **2a**. We substituted potassium fluoride with the hypothesis that a milder noncarbonate base would be more compatible and indeed, under these conditions, the reaction proceeded in 44% yield (entry 2). We found that inorganic fluoride sources enhanced reactivity irrespective of the metal cation, although sodium fluoride seemed to perform slightly better than the others, giving a 53% yield of **2a** (entry 3; see Scheme S1 for the full optimization). The electron-rich trialkyl phosphine, P(^*t*^Bu)_3_, in the form of the Pd(0) precatalyst, gave a significant increase in yield to 82% (entry 5). We desired to use a bench-stable catalyst precursor and swapped Pd(P(^*t*^Bu)_3_)_2_ for two different precatalysts, P(^*t*^Bu)_3_ Pd G4 and P(^*t*^Bu)_3_Pd(crotyl)Cl, which gave similar yields (entries 6–7). The addition of extra ligands in the form of the phosphonium tetrafluoroborate salt (entry 8) gave a slightly increased yield. Screening of four different commercially available Pd(P(^*t*^Bu)_3_) precursors led us to the conditions in entry 12, which gave **2a** in 93% yield. We found that the effect of fluoride in the reaction was independent of catalyst precursor and necessary for a high yield, as no matter the precatalyst, fluoride provided a notable increase in reactivity versus the same conditions as sans fluoride (entries 8–10, 12). As an alternative route, we attempted Niwa’s zinc-mediated SMC, which gave **2a** in 85% yield (entry 11), but when we attempted to extend the coupling to other important IMiD derivatives (**2b**–**f**), the conditions failed to fully convert starting material to product, which we concluded was at least partially due to the high degree of insolubility of **2b**–**e** in the ethereal solvents required for the reaction to proceed.^16^

**Scheme 2:**
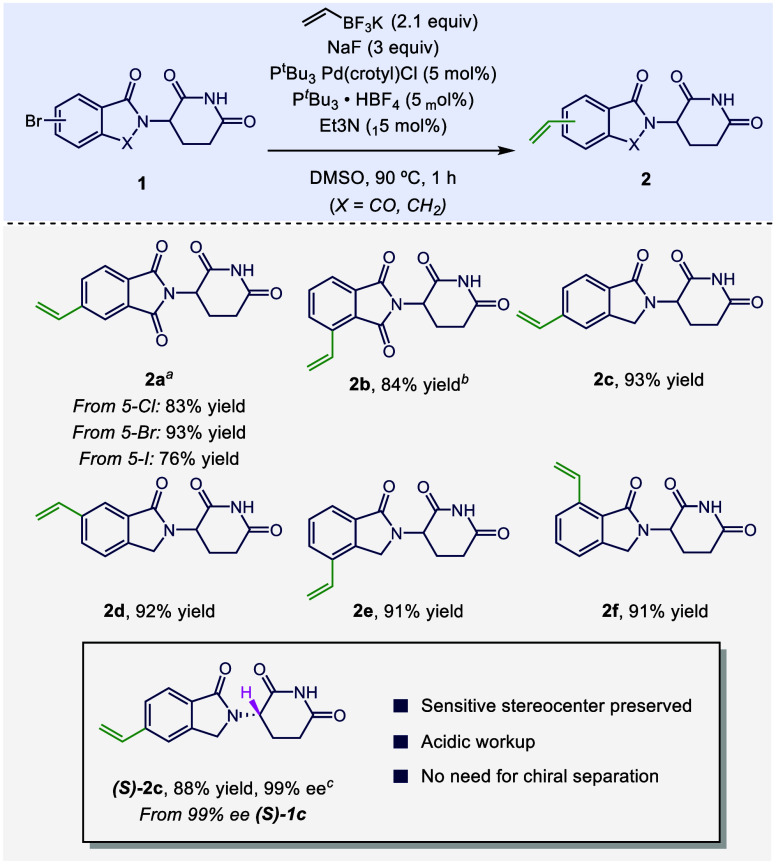
Scope of IMiD-type Compounds Isolated yields reported. ^*a*^Reaction run using 1,4-dioxane as solvent. ^*b*^Reaction conducted using 10 mol % catalyst, 30 mol % Et_3_N, 10 mol % ligand. ^c^Reaction conducted for 0.17 h, using 1M eq. citric acid as the extraction solvent.

In evaluating the generality of these conditions to other IMiD-type structures, we found the conditions in entry 12 to be halide-agnostic, as exchange of the 5-bromo substituent of **1a** with other halides (Cl, I) did not significantly change reaction yields (Scheme 2). These conditions worked just as well in DMSO, which significantly boosted solubility and enabled the generation of **2c**–**2f** in yields over 90% while significantly shortening reaction times from 18 to 1 h. Compound **2b** required an increase in catalyst loading to give an 84% isolated yield. Although the glutarimide stereocenter in IMiD derivatives is easily epimerizable, especially under physiological conditions, the study of optically pure thalidomide derivatives has merit.^11a,21a,−21c^ Studies of IMiD derivatives that focus on one glutarimide enantiomer often require chiral separation or the installation of functionality prior to the formation of the glutarimide.^9a,11a,22^ We found that the existing conditions for vinylation of thalidomide caused complete racemization of this stereocenter.^20^ However, under our conditions, a sample of (*S*)-**1c** in 99% e.e. fully converts to (*S*)-**2c** with complete retention of stereochemistry (Scheme 2). To ensure enantiofidelity, an acidic aqueous wash was employed rather than the typical neutral one.

**Scheme 3:**
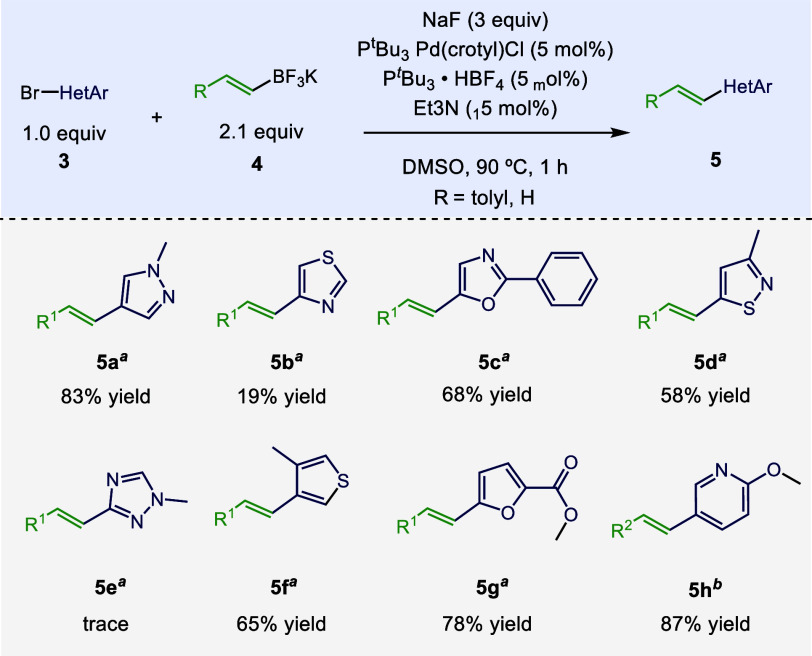
Scope of Heteroaryl Bromides Isolated yields reported. ^*a*^R^1^ = *p*-tolyl. ^*b*^R^2^ = H.

After exploring the scope of compatibility with glutarimide derivatives, we were also interested in whether the method was more generally applicable (Scheme 3). Taking inspiration from Buchwald and co-workers’ work on C–N couplings of base-sensitive 5-membered heterocycles,^23^ we tested the cross-coupling of potassium 4-methyl-ß-styryltrifluoroborate (to avoid working with volatile products) and several heteroaryl bromides (**3a**–**h**). Apart from isothiazole (**5b**) and 1,2,4-triazole (**5e**), all gave good to excellent yields. The method is also perfectly tolerant of methyl esters (**5g**). Furthermore, the vinylation of pyridyl substrate (**5h**), returned an excellent yield, demonstrating the relevance of these conditions to six-membered heterocyclic coupling partners.

**Scheme 4:**
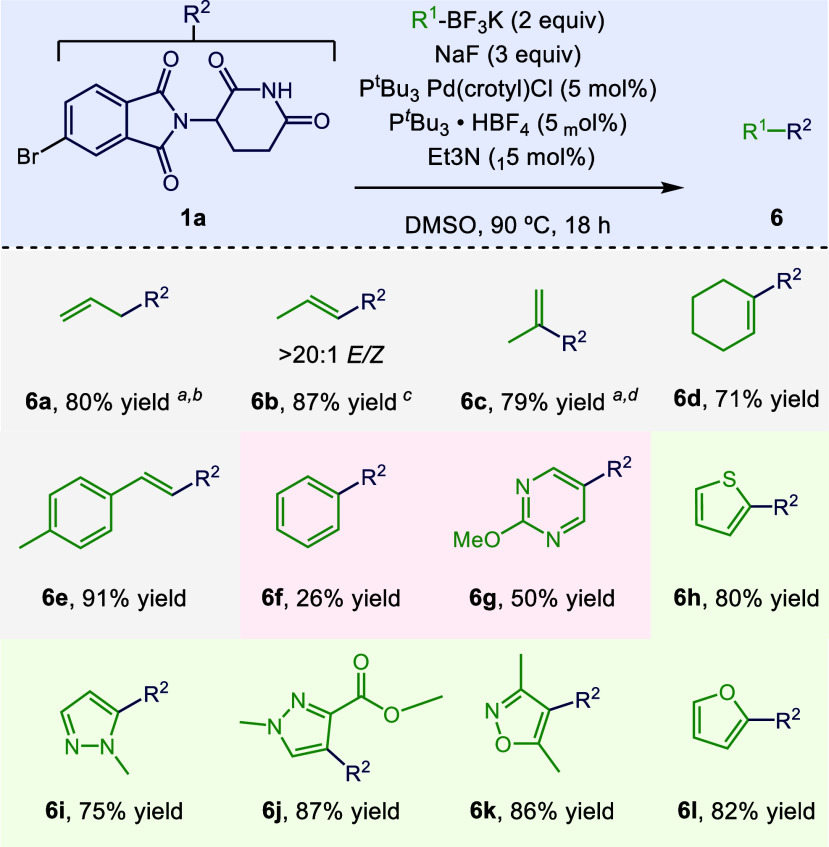
Scope of the Trifluoroborate Coupling Partner Isolated yields reported. ^*a*^Reaction run for 0.17 h using 10 mol % P^*t*^Bu_3_ Pd (crotyl)Cl, 10 mol % P^*t*^Bu_3_ · HBF_4_, 30 mol % TEA. ^*b*^18:1 ratio of terminal to internal (i.e., Pd-isomerized, i.e., **6a** to **6b**) alkene. ^*c*^>20:1 ratio **6b**:**6a**. ^*d*^7.5:1 raio **6c**:**6b**; standard conditions gave a 1:2 ratio of **6c**:**6b**.

We next investigated the scope of trifluoroborate derivatives under the reaction conditions (Scheme 4). Various alkene derivatives performed very well, as illustrated by **6a**–**e**, although the reaction must be carefully monitored to ensure that off-cycle palladium species do not engage in rearrangement of the subject alkene. For example, in the formation of **6a**, the alkene will begin to rearrange to the more thermodynamically stable product **6b** within 10 min under the reaction conditions. We found a stark contrast between aryl trifluoroborates and alkenyl trifluoroborates. Phenyltrifluoroborate gives only a 26% yield of **6f**. The rate of this reaction is quite slow compared to that of the vinylation; we observed that after 10 min, the reaction conditions gave a 76% yield of **6a**, while the same conditions gave a mere 7% of **6f**. Fortunately, the reaction can be extended to a variety of heteroaryl derivatives, as illustrated for **6g** to **6l**. These conditions do not appear to extend to alkyl trifluoroborates, and we generally recovered both the starting aryl bromide and the trifluoroborate in these cases (Figure S4).

**Figure 2:**
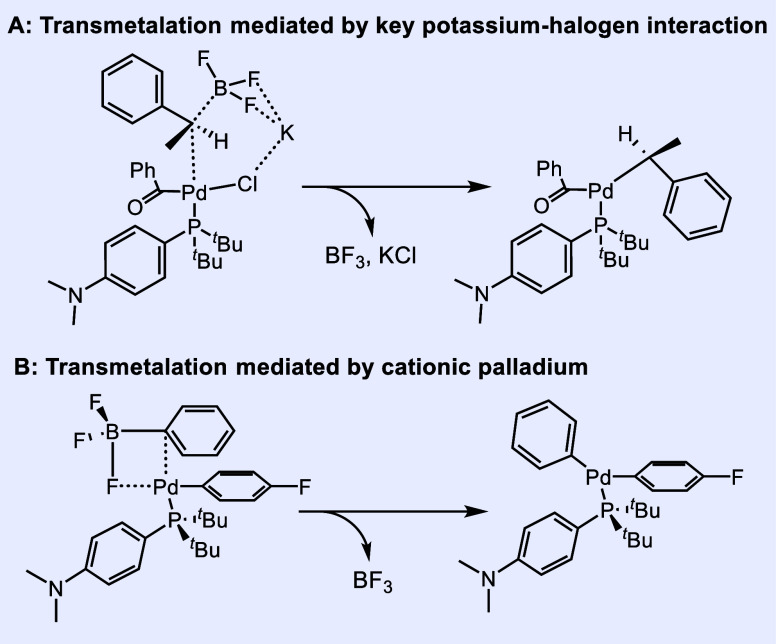
(A) Transmetalation via the potassium–halogen interaction. (B) Transmetalation via cationic palladium.

Trifluoroborates are slow to react under anhydrous conditions^25^ except in special cases; typically, hydrolytic conditions are required to convert the trifluoroborate to a more reactive boronic acid in situ.^18^ One case of transmetalation under anhydrous conditions relies on electrophilic acyl halides (Figure 2A),^26^ in which an alkyl trifluoroborate is transferred to palladium via a key potassium-mediated interaction, releasing BF_3_ and KCl in the process.

Another instance involves the activation of palladium toward transmetalation via complete or partial abstraction of the halide by Lewis acids.^16,26^ This is exemplified by Niwa’s proposed mechanism in Figure 2B, which proceeds through a four-center transition state, releasing BF_3_ as the substrate is transferred to palladium.^16^ Thus, we found it unusual that the cross-coupling of vinyl trifluoroborate proceeded so easily under our conditions.

**Figure 3:**
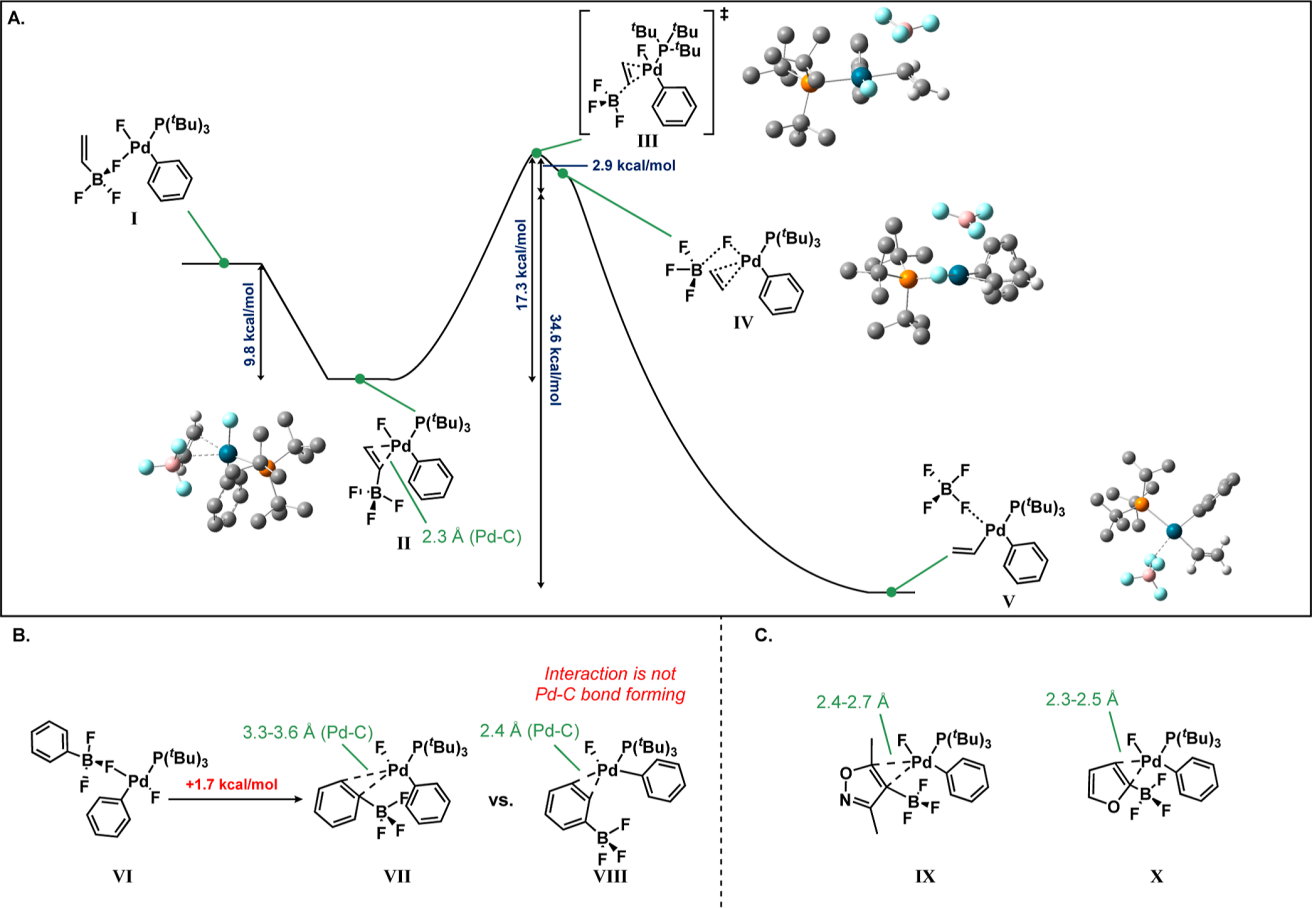
(A) Computed structures of association and rearrangement complexes of vinylborate. Δ*G* demonstrates the favorability of this transformation for vinylborate salts. (B) Computed structures of productive and nonproductive association and rearrangement complexes of phenylborate. Δ*G* as well as elongated pi-bonding interactions demonstrate that this transformation for phenylborate salts is less favorable. (C) Computed structures of association for a more hindered isoxazoleborate and furanborate reveal the ability to form vinyl-like association complexes despite aromaticity or increased steric demand.

To better probe the reaction, we turned to computational studies, conducting calculations at the M06/6-31G* level of theory (complete computational details of which can be found in the Supporting Information). A proposed “association complex”, wherein a fluoride of the trifluoroborate associates with palladium, was first computed. All possible isomeric arrangements were studied (see Supporting Information). The most stable of these features is the vinyl trifluoroborate transoid to the phosphine and the cisoid to the model phenyl ring (Figure 3A, **I**, see Supporting Information for all isomers). To allow for the formation of the pretransmetalation complex, we then proposed the rearrangement of the borate to a π-bound motif (**II**). The Δ*G* for this transformation indeed demonstrates the favorability of this rearrangement, with an exotherm of 9.8 kcal/mol (Figure 3A). π-Complex **II** forms transition state **III** with a Δ*G*^‡^ of 17.3 kcal/mol. Weakening of the B–C bond and association with fluoride (**IV**) leads to a metal center primed for reductive elimination (**V**), with an exotherm of 20.2 kcal/mol (Figure 3A; the exotherm is relative to the rearrangement complex).

We have also conducted some parallel computational studies on the reaction with phenyl trifluoroborate, which is far less effective in this reaction. In this case, the π-complex is far less favorable, requiring an endothermic reaction (Figure 3B) compared to an exothermic rearrangement with the vinyltrifluoroborate. Furthermore, efforts to find the analogous transition state for the transmetalation were unsuccessful. These results suggest that either the phenyl transfer is a far less favorable process under the mechanism observed for the vinyltrifluoroborate or it proceeds by another mechanism, such as a potassium-mediated process. Calculations with and without potassium countercations were performed, and in both instances, the ground state trend in free energy was upheld. We attribute the differing favorability of π-complex formation between substrates primarily to sterics: the distances to the Pd center are elongated for the phenyl trifluoroborate π-complex relative to the vinyl trifluoroborate (3.3–3.6 Å, **VII** vs 2.3 Å, **II** respectively).

In the only instance in which a shorter interaction between Pd and C for the phenylborate was identified (**VIII**, 2.4 Å), the carbons of the ring playing a role in the π-bonding interaction were not those adjacent to the borate, and thus it is not a species that plays a role in reactivity (Figure 3B). To further confirm that this hypothesis was consistent with experimental findings, Pd–C bond distances for the borate π-complexes of the isoxazole leading to **6k** (**IX**) and furan (**X**) leading to **6l**, both better able to tolerate the disruption of aromaticity, were explored (Figure 3C). Computational results showed that these borates were able to form complexes with bond distances similar to the vinylborate (2.3–2.7 Å vs 2.3 Å, respectively), which correlates well with the higher yields observed experimentally.

The use of fluoride in cross-coupling reactions can be traced back to 1992, and due to the work of Fu, Le Duc, and others, the role of fluoride is understood, but only under hydrolytic conditions.^27a−27g^ In Fu’s 2000 work,^24^ the authors use potassium fluoride as a base and propose an intermediate trifluoroborate, but go on to demonstrate the complete inability of potassium *o*-tolyl trifluoroborate to couple with an aryl chloride under their conditions. Taking our finding that the effect of fluoride on the reaction was precatalyst-agnostic (vide supra), along with the fact that various Lewis- and Brønsted-Lowry basic reaction additives failed to provide an effect of similar magnitude (Scheme S2), we propose that the effects of inorganic fluoride play a crucial role in this reaction.

## Conclusions

During our research, we discovered conditions that were generally applicable to coupling alkene-type potassium trifluoroborates with various glutarimide-containing structures, as well as base-sensitive heteroarenes. We found that fluoride played a key role in enhancing the system. We also found that the reaction conditions were mild enough to preserve chiral fidelity at the sensitive glutarimide stereocenter. Further computational studies revealed a π-complex potentially formed during pretransmetalation that helped explain a clear preference of the reaction toward alkene-type trifluoroborates, as well as arene-type trifluoroborates that possess a relatively lower degree of aromaticity, over those that possess a relatively higher degree of aromaticity. Overall, this study represents an addition to the methodological toolbox available to chemists interested in preparing derivatives of glutarimide-containing compounds and could facilitate the discovery of new CELMoDs that have the potential to impact human health.

## Experimental Section

### General Methods

All reactions were conducted in flame-dried glassware under an inert atmosphere of dry nitrogen or argon, unless otherwise noted. All reagents were purchased from Oakwood, Combi-Blocks, Millipore Sigma, Strem, or Ambeed and used as received. Anhydrous dimethyl sulfoxide (DMSO), 1,4-dioxane, and all other solvents were purchased from Fisher Scientific and used as received. Proton (^1^H) NMR spectra were recorded at 400 MHz on Varian and Bruker spectrometers, 500 MHz on an Inova-500 spectrometer, or 600 MHz on an Inova-600 spectrometer. Carbon-13 (^13^C{^1^H}) NMR spectra were recorded at 100 MHz on Varian and Bruker spectrometers or 151 MHz on an Inova-600 spectrometer. NMR spectra were recorded in deuterated solvents and were referenced to tetramethylsilane (in the case of CDCl_3_) or the respective residual solvent signal. NMR chemical shifts were reported in parts per million (ppm). Abbreviations for signal couplings are as follows: s, singlet; d, doublet; t, triplet; q, quartet; quin, quintet; sex, sextet; sep, septet; and m, multiplet. The coupling constants were taken from the spectra directly and are uncorrected. Fourier transform infrared (FTIR) spectra were collected on a Nicolet Impact Series 10 FT-IR equipped with an attenuated total reflection apparatus. Mass spectrometric determinations were carried out on a Thermo Finnigan LTQ-FTMS spectrometer with atmospheric pressure chemical ionization (APCI), using a Fourier transform ion cyclotron resonance mass analyzer. Optical rotations were measured on a Rudolph Research Analytical Automatic Polarimeter, APIV-1W. Analytical thin layer chromatography was performed on silica gel plates using ultraviolet light to visualize analytes. Normal phase column chromatography was performed with SiliCycle silica gel 60 Å (50 μm) hand-packed in Biotage Sfär columns on Biotage Isolera Four chromatographs, with Celite as a dry-loading absorbent. Reverse phase chromatography was conducted using Teledyne ISCO RediSep Gold 18 reverse-phase columns using Teledyne ISCO CombiFlash or Biotage Isolera chromatographs. Supercritical fluid chromatography (SFC) analysis was performed on a Water Acquity UPC2 instrument. Melting points were measured on an electrothermal IA6304 melting point apparatus.

Caution! Glutarimide-containing compounds such as thalidomide are known reproductive, neurological, and hematological toxins. Care must be exercised to avoid direct contact with glutarimide-containing material; any glassware used with glutarimide-containing material should be treated with a 2 M aqueous solution of a strong base such as sodium hydroxide to destroy the material.

#### General Procedure **A** for Reaction Optimization

A septum-cap vial was equipped with a PTFE magnetic stir bar, flame-dried under a vacuum, and then allowed to cool under N_2_ atmosphere. The vial was charged with 5-bromo-2-(2,6-dioxopiperidin-3-yl)isoindoline-1,3-dione (169 mg, 1.0 equiv, 0.50 mmol), sodium fluoride (63 mg, 3 equiv, 1.50 mmol), chloro(crotyl)(tri*tert*-butylphosphine)palladium(II) (10 mg, 5 mol %, 25.0 μmol), tri*tert*-butylphosphoniumtetrafluoroborate (7 mg, 5 mol %, 25.0 μmol), and potassium trifluoro(vinyl)borate (142 mg, 2.12 equiv, 1.06 mmol) under backflow of nitrogen. Dry 1,4-dioxane (1.71 mL) and triethylamine (10.5 μL, 15 mol %, 75.0 μmol) were charged by syringe, and the vial was equipped with an argon-filled balloon. The vial was placed in an aluminum heating block preheated to 90 °C and stirred at this temperature for 24 h. The reaction was concentrated in vacuo onto Celite and purified by flash column chromatography (20–50% EtOAc/hexanes). The product-containing fractions were aggregated and concentrated in vacuo, affording 2-(2,6-dioxopiperidin-3-yl)-5-vinylisoindoline-1,3-dione as an amorphous tan to off-white solid.

#### General Procedure **B-1** for the Cross-Coupling of Potassium Trifluoro(vinyl)borates and Aryl Halides

A septum-cap vial was equipped with a PTFE magnetic stir bar, flame-dried under a vacuum, and then allowed to cool under an N_2_ atmosphere. The vial was charged with aryl halide (1.0 equiv, 0.50 mmol), sodium fluoride (63 mg, 3 equiv, 1.50 mmol), chloro(crotyl)(tri*tert*-butylphosphine)palladium(II) (10 mg, 5 mol %, 25.0 μmol), tri*tert*-butylphosphoniumtetrafluoroborate (7 mg, 5 mol %, 25.0 μmol), and potassium trifluoro(vinyl)borate (142 mg, 2.12 equiv, 1.06 mmol) under the backflow of nitrogen. Dry DMSO (0.70 mL) and triethylamine (10.5 μL, 15 mol %, 75.0 μmol) were charged by syringe, and the vial was equipped with an argon-filled balloon. The vial was placed in an aluminum heating block preheated to 90 °C and stirred at this temperature for 1 h. The reaction was partitioned between ethyl acetate (25 mL) and water (50 mL). The aqueous layer was extracted thrice with 25 mL portions of ethyl acetate, after which the combined organic layers were washed with a 10% w/w aqueous solution of lithium chloride. The organic layers were dried over magnesium sulfate, then filtered and concentrated in vacuo. The material was purified via reverse phase chromatography (C_18_, 10–100% MeCN/H_2_O, 0.1% v/v TFA) and/or normal phase column chromatography with an appropriate mixture of solvents (SiO_2_).

#### General Procedure **B-2** for the Cross-Coupling of Potassium Trifluoroborates and Aryl Bromides

A septum-cap vial was equipped with a PTFE magnetic stir bar and flame-dried under vacuum, then allowed to cool under an N_2_ atmosphere. The vial was charged with aryl halide (1.0 equiv, 0.50 mmol), sodium fluoride (63 mg, 3 equiv, 1.50 mmol), chloro(crotyl)(tri*tert*-butylphosphine)palladium(II) (10 mg, 5 mol %, 25.0 μmol), tri*tert*-butylphosphoniumtetrafluoroborate (7 mg, 5 mol %, 25.0 μmol), and potassium trifluoro(vinyl)borate (142 mg, 2.12 equiv, 1.06 mmol) under the backflow of nitrogen. Dry DMSO (0.70 mL) and triethylamine (10.5 μL, 15 mol %, 75.0 μmol) were charged by syringe, and the vial was equipped with an argon-filled balloon. The vial was placed in an aluminum heating block preheated to 90 °C and stirred at this temperature for 18 h. The reaction was partitioned between ethyl acetate (25 mL) and water (50 mL). The aqueous layer was extracted thrice with 25 mL portions of ethyl acetate, after which the combined organic layers were washed with a 10% w/w aqueous solution of lithium chloride. The organic layers were dried over magnesium sulfate, then filtered and concentrated in vacuo. The material was purified via reverse phase chromatography (C_18_, 10–100% MeCN/H_2_O, 0.1% v/v TFA) and/or normal phase column chromatography with an appropriate mixture of solvents (SiO_2_).

#### General Procedure **B-3** for the 1 mmol-Scale Cross-Coupling of Potassium Trifluoro(vinyl)borate and 5-Bromo-2-(2,6-dioxopiperidin-3-yl)isoindoline-1,3-dione

A septum-cap vial was equipped with a PTFE magnetic stir bar, flame-dried under a vacuum, and then allowed to cool under a N_2_ atmosphere. The vial was charged with 5-bromo-2-(2,6-dioxopiperidin-3-yl)isoindoline-1,3-dione (337 mg, 1.0 equiv, 1.00 mmol), sodium fluoride (126 mg, 3.0 equiv, 3.00 mmol), chloro(crotyl)(tri*tert*-butylphosphine)palladium(II) (21 mg, 5 mol %, 50.0 μmol), tri*tert*-butylphosphoniumtetrafluoroborate (15 mg, 5 mol %, 50.0 μmol), and potassium trifluoro(vinyl)borate (284 mg, 2.12 equiv, 2.12 mmol) under the backflow of nitrogen. Dry DMSO (1.71 mL) and triethylamine (10.5 μL, 15 mol %, 75.0 μmol) were charged by syringe, and the vial was equipped with an argon-filled balloon. The vial was placed in an aluminum heating block preheated to 90 °C and stirred at this temperature for 24 h. The reaction was partitioned between ethyl acetate (25 mL) and water (50 mL). The aqueous layer was extracted thrice with 25 mL portions of ethyl acetate, after which the combined organic layers were washed with a 10% w/w aqueous solution of lithium chloride. The organic layers were dried over magnesium sulfate, then filtered and concentrated in vacuo. The material was concentrated in vacuo onto Celite and purified by flash column chromatography (20–50% EtOAc/hexanes). The product-containing fractions were aggregated and concentrated in vacuo, affording 2-(2,6-dioxopiperidin-3-yl)-5-vinylisoindoline-1,3-dione (261 mg, 0.92 mmol, 92% yield) as a fine, amorphous tan solid.

#### General Procedure **C** for the Stereoretentive Cross-Coupling of Potassium Trifluoroborates and Thalidomide Derivatives

A septum-cap vial was equipped with a PTFE magnetic stir bar, flame-dried under a vacuum, and then allowed to cool under N_2_ atmosphere. The vial was charged with thalidomide derivative (81 mg, 1.0 equiv, 0.25 mmol), sodium fluoride (32 mg, 3.0 equiv, 0.75 mmol), potassium trifluoro(vinyl)borate (71 mg, 2.12 equiv, 0.53 μmol), chloro(crotyl)(tri*tert*-butylphosphine)palladium(II) (5 mg, 5 mol % 13 μmol), and tri*tert*-butylphosphonium tetrafluoroborate (4 mg, 5 mol %, 13 μmol) under the backflow of nitrogen. Dry DMSO (0.9 mL) and triethylamine (5.2 μL, 15 mol %, 38 μmol) were charged by a syringe. The vial was placed in an aluminum heating block preheated to 90 °C and stirred at this temperature for 10 min. The reaction was partitioned between 20 mL of 1 M aqueous citric acid solution and 10 mL of ethyl acetate. The aqueous layer was extracted thrice with 10 mL portions of ethyl acetate, which was dried over magnesium sulfate and evaporated in vacuo. The material was purified by reverse-phase chromatography (10–100% MeCN/H_2_O, 0.1% v/v TFA).

#### 2-(2,6-Dioxopiperidin-3-yl)-5-vinylisoindoline-1,3-dione (**2a**)

Compound **2a** was prepared via General Procedure **A** using **1a** (169 mg, 1.0 equiv, 0.50 mmol), sodium fluoride (63 mg, 3 equiv, 1.50 mmol), chloro(crotyl)(tri*tert*-butylphosphine)palladium(II) (10 mg, 5 mol %, 25.0 μmol), tri*tert*-butylphosphoniumtetrafluoroborate (7 mg, 5 mol %, 25.0 μmol), **4** (142 mg, 2.12 equiv, 1.06 mmol), dry 1,4-dioxane (1.71 mL), and triethylamine (10.5 μL, 15 mol %, 75.0 μmol). The material was purified by flash column chromatography (20–50% EtOAc/hexanes, SiO_2_). The product-containing fractions were aggregated and concentrated in vacuo, affording **2a** (132 mg, 0.46 mmol, 93% yield) as a fine amorphous tan solid. ^1^H NMR (500 MHz, DMSO-*d*_6_): δ 11.15 (s, 1H), 8.06 (s, 1H), 7.95 (d, *J* = 7.6 Hz, 1H), 7.89 (d, *J* = 7.6 Hz, 1H), 6.95 (dd, *J* = 17.6, 11.0 Hz, 1H), 6.20 (d, *J* = 17.6 Hz, 1H), 5.54 (d, *J* = 11.0 Hz, 1H), 5.16 (dd, *J* = 12.7, 5.2 Hz, 1H), 2.93–2.86 (m, 1H), 2.62–2.50 (m, 2H, partially obscured by solvent signal), 2.08–2.05 (m, 1H). ^13^C{^1^H} NMR (101 MHz, DMSO): δ 172.8, 169.9, 167.1, 166.9, 143.8, 135.2, 132.4, 132.1, 130.1, 123.9, 120.7, 119.1, 49.1, 31.0, 22.0. HRMS (APCI) *m*/*z*: [M + H]^+^ calcd for C_15_H_13_O_4_N_2_, 285.0870; found, 285.0868. FTIR (neat) ν_max_/cm^–1^: 3468, 3202, 3101, 2990, 2904, 1773, 1695, 1616.

#### 2-(2,6-Dioxopiperidin-3-yl)-4-vinylisoindoline-1,3-dione (**2b**)

Compound **2b** was prepared via a modification of General Procedure **B-1** using **1b** (169 mg, 1.0 equiv, 0.50 mmol), sodium fluoride (63 mg, 3 equiv, 1.50 mmol), chloro(crotyl)(tri*tert*-butylphosphine)palladium(II) (21 mg, 10 mol %, 50.0 μmol), tri*tert*-butylphosphoniumtetrafluoroborate (15 mg, 10 mol %, 10.0 μmol), **4** (142 mg, 2.12 equiv, 1.06 mmol), dry DMSO (1.71 mL), and triethylamine (21 μL, 30 mol %, 0.15 mmol). The material was purified by flash column chromatography (0–10% MeOH/CH_2_Cl_2_, SiO_2_), affording **2b** (120 mg, 0.42 mmol, 84% yield) as a fine, amorphous tan solid. ^1^H NMR (500 MHz, DMSO-*d*_6_): δ 11.13 (s, 1H), 8.21–8.14 (m, 1H), 7.87–7.78 (m, 1H), 7.66 (dd, *J* = 17.8, 11.1 Hz, 1H), 6.23 (dd, *J* = 17.8, 0.9 Hz, 1H), 5.66 (dd, *J* = 11.1, 0.9 Hz, 1H), 5.15 (dd, *J* = 12.8, 5.4 Hz, 11H), 2.89 (ddd, *J* = 16.9, 13.9, 5.4 Hz, 1H), 2.66–2.50 (m, 2H, partially obscured by solvent signal), 2.13–1.97 (m, 1H). ^13^C{^1^H} NMR (101 MHz, DMSO-*d*_6_): δ 172.8, 169.9, 167.6, 166.8, 135.7, 134.7, 131.8, 130.6, 129.8, 125.9, 122.7, 120.6, 48.9, 30.9, 21.9. HRMS (APCI) *m*/*z*: [M + H]^+^ requires 285.0870, calcd for C_15_H_13_O_4_N_2_; found, 285.0868. FTIR (neat) ν_max_/cm^–1^: 3202, 3097, 2920, 1774, 1698, 1391, 1370, 1262, 1201. 3-(1-Oxo-5-vinylisoindolin-2-yl)piperidine-2,6-dione (2c, racemic material), (*S*)-3-(1-oxo-5-vinylisoindolin-2-yl)piperidine-2,6-dione ((*S*)-2c, enantioenriched material). Compound **2c** was prepared via General Procedure **B-1** using **1c**, (162 mg, 1.0 equiv, 0.50 mmol), sodium fluoride (63 mg, 3 equiv, 1.50 mmol), chloro(crotyl)(tri*tert*-butylphosphine)palladium(II) (10 mg, 5 mol %, 25.0 μmol), tri*tert*-butylphosphoniumtetrafluoroborate (7 mg, 5 mol %, 25.0 μmol), **4** (142 mg, 2.12 equiv, 1.06 mmol), dry DMSO (1.71 mL), and triethylamine (10.5 μL, 15 mol %, 75.0 μmol). The material was purified by flash column chromatography (0–3% MeOH/CH_2_Cl_2_, SiO_2_), affording **2c** (126 mg, 0.47 mmol, 93% yield) as a fine, amorphous tan solid. Compound (***S***)-**2c** was prepared via General Procedure **C** using (***S***)-**1c** (99% ee by SFC) (81 mg, 1.0 equiv, 0.25 mmol), sodium fluoride (32 mg, 3.0 equiv, 0.75 mmol), **4** (71 mg, 2.12 equiv, 0.53 μmol), chloro(crotyl)(tri*tert*-butylphosphine)palladium(II) (5 mg, 5 mol % 13 μmol), and tri*tert*-butylphosphonium tetrafluoroborate (4 mg, 5 mol %, 13 μmol), dry DMSO (0.9 mL), and triethylamine (5.2 μL, 15 mol %, 38 μmol). The material was purified by reverse phase chromatography (10–100% MeCN/H_2_O, 0.1% v/v TFA), giving (***S***)-**2c** as a fine, amorphous light-gray solid in 99% ee (59 mg, 0.22 mmol, 88% yield). The same reaction run at 70 °C for 0.5 h gave an 80% yield and 99% ee (54 mg, 0.20 mmol). ^1^H NMR (400 MHz, DMSO-*d*_6_): δ 11.00 (s, 1H), 7.96–7.65 (m, 2H), 7.62 (dd, *J* = 7.9, 1.4 Hz, 1H), 6.87 (dd, *J* = 17.6, 11.0 Hz, 1H), 6.00 (d, *J* = 17.7 Hz, 1H), 5.41 (d, *J* = 11.0 Hz, 1H), 5.12 (dd, *J* = 13.3, 5.1 Hz, 1H), 4.46 (d, *J* = 17.3 Hz, 1H), 4.33 (d, *J* = 17.2 Hz, 1H), 2.92 (ddd, *J* = 17.3, 13.6, 5.4 Hz, 1H), 2.65–2.56 (m, 1H), 2.40 (qd, *J* = 13.2, 4.4 Hz, 1H), 2.01 (ddq, *J* = 10.3, 5.3, 2.5 Hz, 1H). ^13^C{^1^H} NMR (101 MHz, DMSO-*d*_6_): δ 172.9, 171.0, 167.8, 142.7, 140.6, 136.2, 131.1, 126.2, 123.2, 121.0, 116.7, 51.6, 47.1, 31.2, 22.5. HRMS (APCI) *m*/*z*: [M + H]^+^ calcd for C_15_H_15_O_3_N_2_, 271.1077; found, 271.1076. FTIR (neat) ν_max_/cm^–1^: 3079, 2854, 1712, 1677, 1619, 1349, 1198. For (*S*)-**2c**: Specific rotation [α]_D_^23^ – 42.0° (*c* 0.57, DMSO). SFC analysis: major enantiomer (Chiralcel OJ-3, 20% 1:1 MeOH/^*i*^PrOH in CO_2_, 2.5 mL/min, 254 nm) indicated 99% ee: *t*_R_ (major enantiomer) = 1.43 min, *t*_R_ (minor enantiomer) = 1.12 min.

#### 3-(1-Oxo-6-vinylisoindolin-2-yl)piperidine-2,6-dione (**2d**)

Compound **2d** was prepared via General Procedure **B-1** using **1d** (162 mg, 1.0 equiv, 0.50 mmol), sodium fluoride (63 mg, 3 equiv, 1.50 mmol), chloro(crotyl)(tri*tert*-butylphosphine)palladium(II) (10 mg, 5 mol %, 25.0 μmol), tri*tert*-butylphosphoniumtetrafluoroborate (7 mg, 5 mol %, 25.0 μmol), **4** (142 mg, 2.12 equiv, 1.06 mmol), dry DMSO (1.71 mL), and triethylamine (10.5 μL, 15 mol %, 75.0 μmol). The material was purified by flash column chromatography (0–10% MeOH/CH_2_Cl_2_, SiO_2_), affording **2d** (126 mg, 0.47 mmol, 92% yield) as a fine, amorphous tan solid. ^1^H NMR (600 MHz, DMSO-*d*_6_): δ 10.99 (s, 1H), 7.82 (d, *J* = 1.5 Hz, 1H), 7.75 (d, *J* = 1.6 Hz, 1H), 7.59 (d, *J* = 7.9 Hz, 1H), 6.87 (dd, *J* = 17.7, 11.0 Hz, 1H), 5.98 (d, *J* = 18.1 Hz, 1H), 5.34 (d, *J* = 11.0 Hz, 1H), 5.13 (dd, *J* = 13.3, 5.1 Hz, 1H), 4.46 (d, *J* = 17.4 Hz, 1H), 4.33 (d, *J* = 17.4 Hz, 1H), 2.91 (ddd, *J* = 17.3, 13.7, 5.4 Hz, 1H), 2.64–2.56 (m, 1H), 2.40 (qd, *J* = 13.3, 4.4 Hz, 1H), 2.01 (dtd, *J* = 12.7, 5.3, 2.3 Hz, 1H). ^13^C{^1^H} NMR (151 MHz, DMSO): δ 172.9, 171.0, 167.9, 141.6, 137.2, 136.0, 132.2, 129.6, 123.8, 120.3, 115.4, 51.6, 47.1, 31.2, 22.4. HRMS (APCI) *m*/*z*: [M + H]^+^ calcd for C_15_H_15_O_3_N_2_, 271.1077; found, 271.1076. FTIR (neat) ν_max_/cm^–1^: 2967, 2365, 1739, 1664, 1371, 1271.

#### 3-(1-Oxo-4-vinylisoindolin-2-yl)piperidine-2,6-dione (**2e**)

Compound **2e** was prepared via General Procedure **B-1** using **1e** (162 mg, 1.0 equiv, 0.50 mmol), sodium fluoride (63 mg, 3 equiv, 1.50 mmol), chloro(crotyl)(tri*tert*-butylphosphine)palladium(II) (10 mg, 5 mol %, 25.0 μmol), tri*tert*-butylphosphoniumtetrafluoroborate (7 mg, 5 mol %, 25.0 μmol), **4** (142 mg, 2.12 equiv, 1.06 mmol), dry DMSO (1.71 mL), and triethylamine (10.5 μL, 15 mol %, 75.0 μmol). The material was purified by flash column chromatography (0–10% MeOH/CH_2_Cl_2_, SiO_2_), affording **2e** (126 mg, 0.47 mmol, 91% yield) as a fine, amorphous tan solid. ^1^H NMR (400 MHz, DMSO-*d*_6_): δ 11.03 (s, 1H), 7.80 (d, *J* = 7.6 Hz, 1H), 7.66 (d, *J* = 7.4 Hz, 1H), 7.54 (t, *J* = 7.6 Hz, 1H), 6.83 (dd, *J* = 17.8, 11.3 Hz, 1H), 5.90 (d, *J* = 17.7 Hz, 1H), 5.50 (d, *J* = 11.3 Hz, 1H), 5.15 (dd, *J* = 13.3, 5.1 Hz, 1H), 4.57 (d, *J* = 17.5 Hz, 1H), 4.41 (d, *J* = 17.6 Hz, 1H), 2.93 (ddd, *J* = 17.2, 13.7, 5.4 Hz, 1H), 2.74–2.56 (m, 1H), 2.46–2.27 (m, 1H, partially obscured by solvent signal at 2.50 ppm), 2.11–1.72 (m, 1H). ^13^C{1H} NMR (101 MHz, DMSO-*d*_6_): δ 172.9, 171.0, 167.9, 139.3, 132.8, 132.4, 132.1, 129.1, 128.5, 122.5, 117.9, 51.6, 47.0, 31.2, 22.5. HRMS (APCI) *m*/*z*: [M + H]^+^ calcd for C_15_H_15_O_3_N_2_, 271.1077; found, 271.1075. FTIR (neat) ν_max_/cm^–1^: 3712, 3083, 3016, 2914, 1706, 1660, 1628.

#### 3-(1-Oxo-7-vinylisoindolin-2-yl)piperidine-2,6-dione (**2f**)

Compound **2f** was prepared via General Procedure **B-1** using **1f** (50 mg, 1.0 equiv, 0.16 mmol), sodium fluoride (20 mg, 3 equiv, 0.46 mmol), chloro(crotyl)(tri*tert*-butylphosphine)palladium(II) (3.2 mg, 5 mol %, 7.7 μmol), tri*tert*-butylphosphoniumtetrafluoroborate (2.2 mg, 5 mol %, 7.7 μmol), **4** (44 mg, 2.12 equiv, 0.33 mmol), dry DMSO (0.53 mL), and triethylamine (3.2 μL, 15 mol %, 23 μmol). The material was purified by flash column chromatography (10–100% MeCN/H_2_O, 0.1% v/v TFA, C_18_), followed by a second purification (0–10% MeOH/CH_2_Cl_2_, SiO_2_), affording **2f** (38 mg, 0.14 mmol, 91% yield) as an amorphous solid. ^1^H NMR (500 MHz, DMSO-*d*_6_): δ 11.00 (s, 1H), 7.99 (dd, *J* = 17.9, 11.1 Hz, 1H), 7.79 (dt, *J* = 7.8, 0.7 Hz, 1H), 7.59 (td, *J* = 7.6, 0.7 Hz, 1H), 7.50 (dd, *J* = 7.5, 0.9 Hz, 1H), 6.02 (dd, *J* = 17.9, 1.2 Hz, 1H), 5.44 (dd, *J* = 11.1, 1.2 Hz, 1H), 5.10 (dd, *J* = 13.3, 5.1 Hz, 1H), 4.42 (d, *J* = 17.2 Hz, 1H), 4.29 (d, *J* = 17.2 Hz, 1H), 2.91 (ddd, *J* = 17.4, 13.7, 5.4 Hz, 1H), 2.60 (dddd, *J* = 17.4, 4.5, 2.3, 1.0 Hz, 1H), 2.39 (qd, *J* = 13.6, 4.5 Hz, 1H), 2.00 (dtd, *J* = 12.7, 5.4, 2.3 Hz, 1H). ^13^C{^1^H}: NMR (101 MHz, DMSO-*d*_6_): δ 173.0, 171.1, 168.5, 142.7, 135.3, 131.6, 130.9, 127.0, 123.5, 122.8, 117.0, 51.5, 46.6, 31.3, 22.4. HRMS (APCI) *m*/*z*: [M + H]^+^ calcd for C_15_H_15_O_3_N_2_, 271.1077; found, 271.1076. FTIR (neat) ν_max_/cm^–1^: 3195, 3099, 2979, 2193, 1732, 0693, 1590, 1454, 1411, 1484, 1226, 1198, 1179, 1042, 1004, 949, 931, 860, 800.

#### (*E*)-1-Methyl-4-(4-methylstyryl)-1*H*-pyrazole (**5a**)

Compound **5a** was prepared via General Procedure **B-1** using **3a** (51.7 μL, 1.0 equiv, 0.50 mmol), sodium fluoride (63 mg, 3 equiv, 1.50 mmol), chloro(crotyl)(tri*tert*-butylphosphine)palladium(II) (10 mg, 5 mol %, 25.0 μmol), tri*tert*-butylphosphoniumtetrafluoroborate (7 mg, 5 mol %, 25.0 μmol), **4e** (142 mg, 2.12 equiv, 1.06 mmol), dry DMSO (1.71 mL), and triethylamine (10.5 μL, 15 mol %, 75.0 μmol). The material was purified by flash column chromatography (20–30% EtOAc/hexanes, SiO_2_). The product-containing fractions were aggregated and concentrated in vacuo, affording **5a** (82 mg, 0.41 mmol, 83% yield) as a crystalline tan solid. ^1^H NMR indicated a >20:1 *E*/*Z* ratio of the alkene. ^1^H NMR (400 MHz, CDCl_3_): δ 7.66 (s, 1H), 7.44 (s, 1H), 7.33 (d, *J* = 8.1 Hz, 2H), 7.14 (d, *J* = 7.8 Hz, 2H), 6.88 (d, *J* = 16.4 Hz, 1H), 6.79 (d, *J* = 16.4 Hz, 1H), 3.90 (s, 3H), 2.34 (s, 3H). ^13^C NMR{^1^H} NMR (101 MHz, CDCl_3_): δ 137.3, 137.0, 134.9, 129.4, 127.8, 126.9, 125.9, 121.2, 117.7, 39.1, 21.3. HRMS (APCI) *m*/*z*: [M + H]^+^ calcd for C_13_H_15_N_2_, 199.1230; found, 199.1228. FTIR (film) ν_max_/cm^–1^: 2915, 1738, 1638, 1510, 1407, 967, 624. mp: 129–131 °C.

#### (*E*)-4-(4-Methylstyryl)thiazole (**5b**)

Compound **5b** was prepared via General Procedure **B-1** using **3b** (17.8 μL, 1.0 equiv, 0.20 mmol), sodium fluoride (25 mg, 3.0 equiv, 0.60 mmol), chloro(crotyl)(tri*tert*-butylphosphine)palladium(II) (4 mg, 5 mol %, 10.0 μmol), tri*tert*-butylphosphoniumtetrafluoroborate (3 mg, 5 mol %, 10.0 μmol), **4e** (142 mg, 2.12 equiv, 0.42 mmol), dry DMSO (0.70 mL), and triethylamine (4.2 μL, 15 mol %, 30.0 μmol). The material was purified by flash column chromatography (0–40% EtOAc/hexanes, SiO_2_). The product-containing fractions were aggregated and concentrated in vacuo, affording **5b** as an amorphous white solid (7 mg, 0.03 mmol, 19% yield). ^1^H NMR indicated a >20:1 *E*/*Z* of the alkene. ^1^H NMR (600 MHz, CDCl_3_): δ 8.82 (d, *J* = 2.0 Hz, 1H), 7.49 (d, *J* = 16.0 Hz, 1H), 7.43 (d, *J* = 8.1 Hz, 2H), 7.19 (d, *J* = 2.0 Hz, 1H), 7.17 (d, *J* = 7.9 Hz, 2H), 7.12 (d, *J* = 15.9 Hz, 1H), 2.36 (s, 3H). ^13^C NMR{^1^H} (101 MHz, CDCl_3_): δ 155.3, 153.1, 138.1, 134.2, 131.8, 129.6, 126.8, 120.1, 114.4, 21.4. HRMS (APCI) *m*/*z*: [M + H]^+^ calcd for C_12_H_12_N^32^S, 202.0685; found, 202.0684. FTIR (neat) ν_max_/cm^–1^: 3107, 3031, 2920, 2854, 1737, 1680, 1606, 1514, 973, 819, 808.

#### (*E*)-5-(4-Methylstyryl)-2-phenyloxazole (**5c**)

Compound **5c** was prepared via General Procedure **B-1** using **3c** (45 mg, 1.0 equiv, 0.20 mmol), sodium fluoride (25 mg, 3.0 equiv, 0.60 mmol), chloro(crotyl)(tri*tert*-butylphosphine)palladium(II) (4 mg, 5 mol %, 10.0 μmol), tri*tert*-butylphosphoniumtetrafluoroborate (3 mg, 5 mol %, 10.0 μmol), **4e** (142 mg, 2.12 equiv, 0.42 mmol), dry DMSO (0.70 mL), and triethylamine (4.2 μL, 15 mol %, 30.0 μmol). The material was purified by flash column chromatography (% EtOAc/hexanes, SiO_2_). The product-containing fractions were aggregated and concentrated in vacuo, affording **5c** as an amorphous white solid (35 mg, 0.14 mmol, 68% yield). ^1^H NMR indicated a >20:1 *E*/*Z* of the alkene. ^1^H NMR (400 MHz, CDCl_3_): δ 8.16–7.88 (m, 2H), 7.52–7.44 (m, 3H), 7.41 (d, *J* = 8.1 Hz, 2H), 7.21–7.10 (m, 4H), 6.89 (d, *J* = 16.3 Hz, 1H), 2.37 (s, 3H). ^13^C{^1^H} NMR (101 MHz, CDCl_3_): δ 161.0, 150.7, 138.4, 133.7, 130.5, 129.7, 129.6, 128.9, 127.5, 126.6, 126.5, 126.2, 112.3, 21.5. HRMS (APCI) *m*/*z*: [M + H]^+^ calcd for C_18_H_16_ON, 262.1226; found, 262.1225. FTIR (film) ν_max_/cm^–1^: 3202, 2920, 1607, 1535, 1508, 1483, 1449.

#### (*E*)-3-Methyl-5-(4-methylstyryl)isothiazole (**5d**)

Compound **5d** was prepared via General Procedure **B-1** using **3d** (20.9 μL, 1.0 equiv, 0.20 mmol), sodium fluoride (25 mg, 3.0 equiv, 0.60 mmol), chloro(crotyl)(tri*tert*-butylphosphine)palladium(II) (4 mg, 5 mol %, 10.0 μmol), tri*tert*-butylphosphoniumtetrafluoroborate (3 mg, 5 mol %, 10.0 μmol), **4e** (142 mg, 2.12 equiv, 0.42 mmol), dry DMSO (0.70 mL), and triethylamine (4.2 μL, 15 mol %, 30.0 μmol). The material was purified by flash column chromatography (0–10% EtOAc/hexanes, SiO_2_). The product-containing fractions were aggregated and concentrated in vacuo, affording **5d** as an amorphous white solid (25 mg, 0.12 mmol, 58% yield). ^1^H NMR indicated a >20:1 *E*/*Z* of the alkene. ^1^H NMR (400 MHz, CDCl_3_): δ 7.38 (d, *J* = 8.0 Hz, 2H), 7.18 (d, *J* = 7.9 Hz, 2H), 7.13 (d, *J* = 16.2 Hz, 1H), 7.02 (d, *J* = 16.2 Hz, 1H), 6.95 (s, 1H), 2.48 (s, 3H), 2.37 (s, 3H). ^13^C NMR{^1^H} (101 MHz, CDCl_3_): δ 167.6, 165.1, 139.0, 133.8, 133.2, 129.7, 126.9, 121.7, 116.7, 21.5, 19.1. HRMS (APCI) *m*/*z*: [M + H]^+^ calcd for C_13_H_14_N^32^S, 216.0842; found, 216.0841. FTIR (film) ν_max_/cm^–1^: 3028, 2921, 2360, 1739, 1523, 961, 826, 802.

#### (*E*)-3-Methyl-4-(4-methylstyryl)thiophene (**5f**)

Compound **5f** was prepared via General Procedure **B-1** using **3f** (22.3 μL, 1.0 equiv, 0.20 mmol), sodium fluoride (25 mg, 3.0 equiv, 0.60 mmol), chloro(crotyl)(tri*tert*-butylphosphine)palladium(II) (4 mg, 5 mol %, 10.0 μmol), tri*tert*-butylphosphoniumtetrafluoroborate (3 mg, 5 mol %, 10.0 μmol), **4e** (142 mg, 2.12 equiv, 0.42 mmol), dry DMSO (0.70 mL), and triethylamine (4.2 μL, 15 mol %, 30.0 μmol). The material was purified by flash column chromatography (0–1% EtOAc/hexanes, SiO_2_). The product-containing fractions were aggregated and concentrated in vacuo, affording **5f** as an amorphous white solid (28 mg, 0.13 mmol, 65% yield). ^1^H NMR indicated a >20:1 *E*/*Z* of the alkene. ^1^H NMR (400 MHz, CDCl_3_): δ 7.41 (d, *J* = 8.0 Hz, 2H), 7.37 (d, *J* = 3.2 Hz, 1H), 7.18 (d, *J* = 7.9 Hz, 2H), 7.06–6.91 (m, 3H), 2.38 (s, 3H), 2.35 (s, 3H). ^13^C NMR{^1^H} (101 MHz, CDCl_3_): δ 139.3, 137.5, 136.6, 134.9, 129.5, 129.5, 126.4, 121.7, 121.0, 120.4, 21.4, 15.2. HRMS (APCI) *m*/*z*: [M + H]^+^ calcd for C_14_H_15_^32^S, 215.0889; found, 215.0888. FTIR (film) ν_max_/cm^–1^: 3025, 2919, 2862, 1511, 1448, 959, 801, 780, 510.

#### Methyl (*E*)-5-(4-Methylstyryl)furan-2-carboxylate (**5g**)

Compound **5g** was prepared via General Procedure **B-1** using **3g** (41, 1.0 equiv, 0.20 mmol), sodium fluoride (25 mg, 3.0 equiv, 0.60 mmol), chloro(crotyl)(tri*tert*-butylphosphine)palladium(II) (4 mg, 5 mol %, 10.0 μmol), tri*tert*-butylphosphoniumtetrafluoroborate (3 mg, 5 mol %, 10.0 μmol), **4e** (142 mg, 2.12 equiv, 0.42 mmol), dry DMSO (0.70 mL), and triethylamine (4.2 μL, 15 mol %, 30.0 μmol). The material was purified by flash column chromatography (0–20% EtOAc/hexanes, SiO_2_). The product-containing fractions were aggregated and concentrated in vacuo, affording **5g** as an amorphous white solid (38 mg, 0.16 mmol, 78% yield). ^1^H NMR indicated a >20:1 *E*/*Z* of the alkene. ^1^H NMR (400 MHz, CDCl_3_): δ 7.39 (d, *J* = 8.1 Hz, 2H), 7.26 (d, *J* = 16.3 Hz, 1H), 7.20–7.13 (m, 3H), 6.86 (d, *J* = 16.4 Hz, 1H), 6.42 (d, *J* = 3.6 Hz, 1H), 3.91 (s, 3H), 2.36 (s, 3H). ^13^C NMR{^1^H} (101 MHz, CDCl_3_): δ 159.3, 157.3, 143.3, 138.8, 133.5, 131.6, 129.7, 126.9, 120.2, 114.7, 109.5, 52.0, 21.5. HRMS (APCI) *m*/*z*: [M + H]^+^ calcd for C_15_H_15_O_3_, 243.1016; found, 243.1015. FTIR (film) ν_max_/cm^–1^: 3018, 2590, 1716, 1517, 1496, 1300, 994, 1136, 808.

#### 2-Methoxy-5-vinylpyridine (**5h**)

Compound **5h** was prepared via General Procedure **B-1** using **3h** (129 μL, 1.0 equiv, 0.50 mmol), sodium fluoride (63 mg, 3 equiv, 1.50 mmol), chloro(crotyl)(tri*tert*-butylphosphine)palladium(II) (10 mg, 5 mol %, 25.0 μmol), tri*tert*-butylphosphoniumtetrafluoroborate (7 mg, 5 mol %, 25.0 μmol), **4** (142 mg, 2.12 equiv, 1.06 mmol), dry DMSO (1.71 mL), and triethylamine (10.5 μL, 15 mol %, 75.0 μmol). The material was purified by flash column chromatography (0–7% pentane/ether, SiO_2_). The product-containing fractions were aggregated and concentrated in vacuo, affording **5h** (117 mg, 0.87 mmol, 87% yield) as a translucent yellow liquid. ^1^H NMR (600 MHz, CDCl_3_): δ 8.12 (d, *J* = 2.4 Hz, 1H), 7.69 (dd, *J* = 8.6, 2.5 Hz, 1H), 6.72 (d, *J* = 8.6 Hz, 1H), 6.65 (dd, *J* = 17.6, 10.9 Hz, 1H), 5.64 (d, *J* = 18.4 Hz, 1H), 5.21 (d, *J* = 11.0 Hz, 1H), 3.94 (s, 3H). Spectral data were consistent with the literature.^28^

#### 5-Allyl-2-(2,6-dioxopiperidin-3-yl)isoindoline-1,3-dione (**6a**)

A septum-cap vial was equipped with a PTFE magnetic stir bar, flame-dried under vacuum, and then allowed to cool under a N_2_ atmosphere. The vial was charged with **1a** (337 mg, 1.0 equiv, 1.000 mmol), sodium fluoride (126 mg, 3.0 equiv, 3.0 mmol), **4a** (314 mg, 2.12 equiv, 2.12 mmol), chloro(crotyl)(tri*tert*-butylphosphine)palladium(II) (42 mg, 10 mol %, 0.10 mmol), and tri*tert*-butylphosphonium tetrafluoroborate (29 mg, 10 mol %, 0.10 mmol) under the backflow of nitrogen. Dry DMSO (1.71 mL) and triethylamine (42 μL, 30 mol %, 0.30 mmol) were charged by syringe, and the vial was equipped with an argon-filled balloon. The vial was placed in an aluminum heating block preheated to 90 °C and was stirred at this temperature for 10 min. The reaction was partitioned between ethyl acetate (25 mL) and water (50 mL). The aqueous layer was extracted thrice with 25 mL portions of ethyl acetate, after which the combined organic layers were washed with a 10% w/w aqueous solution of lithium chloride. The organic layers were dried over magnesium sulfate, then filtered and concentrated in vacuo. The material was concentrated in vacuo onto Celite and purified by flash column chromatography (30–50% EtOAc/hexanes). The product-containing fractions were aggregated and concentrated in vacuo, affording **6a** (239 mg, 0.80 mmol, 80% yield) as a fine, amorphous white solid. Crude ^1^H NMR indicated a 20:1 ratio of **6a** to the internal alkene product **6b**. ^1^H NMR of the purified product indicated a >20:1 ratio of **6a** to **6b**. ^1^H NMR (600 MHz, CDCl_3_): δ 7.92 (s, 1H), 7.79 (d, *J* = 7.6 Hz, 1H), 7.73–7.66 (m, 1H), 7.63–7.46 (m, 1H), 5.93 (ddt, *J* = 16.8, 10.1, 6.7 Hz, 1H), 5.24–5.05 (m, 2H), 4.96 (dd, *J* = 12.7, 5.4 Hz, 1H), 3.53 (d, *J* = 6.7 Hz, 2H), 2.95–2.68 (m, 3H), 2.14 (ddtd, *J* = 12.5, 6.2, 3.0, 1.7 Hz, 1H). ^13^C{^1^H} NMR (101 MHz, CDCl_3_): δ 171.4, 168.4, 167.5, 167.4, 148.1, 135.5, 134.8, 132.3, 129.8, 124.1, 124.0, 117.8, 49.4, 40.4, 31.5, 22.7. HRMS (APCI) *m*/*z*: [M + H]^+^ calcd for C_16_H_15_O_4_N_2_, 299.1026; found, 299.10255. FTIR (neat) ν_max_/cm^–1^: 3024, 2361, 1735, 1365, 1217.

#### (*E*)-2-(2,6-Dioxopiperidin-3-yl)-5-(prop-1-en-1-yl)isoindoline-1,3-dione (**6b**)

Compound **6b** was prepared via General Procedure **B-2** using **1a** (169 mg, 1.0 equiv, 0.50 mmol), sodium fluoride (63 mg, 3 equiv, 1.50 mmol), chloro(crotyl)(tri*tert*-butylphosphine)palladium(II) (10 mg, 5 mol %, 25.0 μmol), tri*tert*-butylphosphoniumtetrafluoroborate (7 mg, 5 mol %, 25.0 μmol), **4b** (157 mg, 2.12 equiv, 1.06 mmol), dry DMSO (1.71 mL), and triethylamine (10.5 μL, 15 mol %, 75.0 μmol). The material was purified by flash column chromatography (10–100% MeCN/H_2_O, 0.1% v/v TFA, C_18_), followed by a second column purification (30–50% EtOAc/hexanes, SiO_2_), which afforded **6b** (128 mg, 0.43 mmol, 87% yield) as a fine, amorphous white solid. Crude ^1^H NMR indicated a >20:1 ratio of **6b** to the terminal alkene product **6a**. ^1^H NMR of the purified product indicated a >20:1 ratio of **6b** to **6a**. ^1^H NMR indicated a >20:1 *E*/*Z* of the alkene. ^1^H NMR (400 MHz, DMSO-*d*_6_): δ 11.14 (s, 1H), 7.94 (s, 1H), 7.84 (d, *J* = 0.9 Hz, 2H), 6.75–6.58 (m, 2H), 5.14 (dd, *J* = 12.9, 5.4 Hz, 1H), 2.89 (ddd, *J* = 17.3, 14.1, 5.4 Hz, 1H), 2.64–2.52 (m, 2H), 2.06 (dtd, *J* = 12.7, 6.0, 2.9 Hz, 1H), 1.90 (d, *J* = 5.3 Hz, 3H). ^13^C{^1^H} NMR (101 MHz, DMSO-*d*_6_): δ 173.5, 170.6, 167.6, 144.9, 132.8, 132.4, 131.8, 130.2, 129.6, 124.5, 120.8, 49.6, 31.6, 22.7, 19.3. HRMS (APCI) *m*/*z*: [M + H]^+^ calcd for C_16_H_15_O_4_N_2_, 299.1026; found, 299.1029. FTIR (neat) ν_max_/cm^–1^: 3219, 1774, 1616, 1387, 1261, 1199, 1134, 743.

#### 2-(2,6-Dioxopiperidin-3-yl)-5-(prop-1-en-2-yl)isoindoline-1,3-dione (**6c**)

A septum-cap vial was equipped with a PTFE magnetic stir bar, flame-dried under vacuum, and then allowed to cool under a N_2_ atmosphere. The vial was charged with **1a** (67 mg, 1.0 equiv, 0.20 mmol), sodium fluoride (25 mg, 3.0 equiv, 0.60 mmol), **4c** (63 mg, 2.12 equiv, 0.42 mmol), chloro(crotyl)(tri*tert*-butylphosphine)palladium(II) (8.3 mg, 10 mol %, 0.02 mmol), and tri*tert*-butylphosphonium tetrafluoroborate (6 mg, 10 mol %, 0.02 mmol) under the backflow of nitrogen. Dry DMSO (1.71 mL) and triethylamine (8.4 μL, 30 mol %, 0.06 mmol) were charged by syringe, and the vial was equipped with an argon-filled balloon. The vial was placed in an aluminum heating block preheated to 90 °C and stirred at this temperature for 10 min. The reaction was partitioned between ethyl acetate (10 mL) and water (20 mL). The aqueous layer was extracted thrice with 20 mL portions of ethyl acetate, after which the combined organic layers were washed with a 10% w/w aqueous solution of lithium chloride. The organic layers were dried over magnesium sulfate, then filtered and concentrated in vacuo. The material was concentrated in vacuo onto Celite and purified by flash column chromatography (10–100% MeCN/H_2_O, 0.1% v/v TFA, C_18_), followed by a second column purification (40% EtOAc/hexanes, SiO_2_), which afforded **6c** (47 mg, 0.16 mmol, 79% yield) as a fine, amorphous white solid. ^1^H NMR of the crude reaction mixture showed a 7.5:1 ratio of the isoprenyl product **6c** to the rearranged product (**6b**). ^1^H NMR of the purified reaction mixture showed a 6:1 ratio for **6c** to **6b**. ^1^H NMR (400 MHz, DMSO-*d*_6_): δ 11.44 (s, 1H), 8.35–8.06 (m, 3H), 6.03 (s, 1H), 5.66 (s, 1H), 5.47 (dd, *J* = 13.0, 5.3 Hz, 1H), 3.20 (ddd, *J* = 17.3, 14.0, 5.4 Hz, 1H), 2.95–2.82 (m, 2H), 2.37 (ddd, *J* = 10.8, 5.6, 3.2 Hz, 1H). ^13^C{^1^H} NMR (101 MHz, DMSO-*d*_6_): δ 172.8, 169.8, 167.1, 166.9, 147.0, 141.3, 131.8, 131.5, 129.9, 123.6, 120.1, 116.7, 49.0, 30.9, 22.0, 21.3. HRMS (APCI) *m*/*z*: [M + H]^+^ calcd for C_16_H_15_O_4_N_2_, 299.1026; found, 299.1026. FTIR (neat) ν_max_/cm^–1^: 3214, 3100, 2914, 1772, 1694, 1615, 1375, 1193, 1110.

#### 5-(Cyclohex-1-en-1-yl)-2-(2,6-dioxopiperidin-3-yl)isoindoline-1,3-dione (**6d**)

Compound **6d** was prepared via General Procedure **B-2** using **1a** (67 mg, 1.0 equiv, 0.20 mmol), sodium fluoride (25 mg, 3.0 equiv, 0.60 mmol), chloro(crotyl)(tri*tert*-butylphosphine)palladium(II) (4 mg, 5 mol %, 10.0 μmol), tri*tert*-butylphosphoniumtetrafluoroborate (3 mg, 5 mol %, 10.0 μmol), **4d** (80 mg, 2.12 equiv, 0.42 mmol), dry DMSO (0.70 mL), and triethylamine (4.2 μL, 15 mol %, 30.0 μmol). The material was purified by flash column chromatography (10–100% MeCN/H_2_O, 0.1% v/v TFA, C_18_). The product-containing fractions were aggregated and concentrated in vacuo, affording **6d** as a fine, amorphous tan solid (48 mg, 0.14 mmol, 71% yield). ^1^H NMR (600 MHz, Chloroform-*d*): δ 8.14 (s, 1H), 7.87 (d, *J* = 1.2 Hz, 1H), 7.79 (d, *J* = 7.9 Hz, 1H), 7.72 (dd, *J* = 7.9, 1.5 Hz, 1H), 6.35 (tt, *J* = 4.0, 1.7 Hz, 1H), 4.98 (dd, *J* = 12.6, 5.4 Hz, 1H), 2.93–2.88 (m, 1H), 2.84 (qd, *J* = 12.6, 3.9 Hz, 2H), 2.75 (ddd, *J* = 16.8, 13.5, 5.0 Hz, 2H), 2.43 (tt, *J* = 4.0, 1.9 Hz, 2H), 2.27 (tt, *J* = 6.3, 3.1 Hz, 2H), 2.15 (dtd, *J* = 12.5, 4.9, 2.3 Hz, 2H), 1.81 (ddd, *J* = 12.0, 5.8, 3.1 Hz, 2H), 1.69 (ddd, *J* = 12.1, 6.3, 2.9 Hz, 2H). ^13^C{^1^H} NMR (101 MHz, CDCl_3_): δ 171.3, 168.3, 167.7, 167.4, 149.6, 135.4, 132.2, 130.6, 129.5, 129.3, 123.9, 120.3, 49.4, 31.5, 27.4, 26.2, 22.8, 22.8, 21.8. HRMS (APCI) *m*/*z*: [M + H]^+^ calcd for C_19_H_19_O_4_N_2_, 339.1339; found, 339.1338. FTIR (neat) ν_max_/cm^–1^: 3467, 3207, 3090, 2918, 1772, 1699, 1604.

#### (*E*)-2-(2,6-Dioxopiperidin-3-yl)-5-(4-methylstyryl)isoindoline-1,3-dione (**6e**)

Compound **6e** was prepared via General Procedure **B-2** using **1a** (67 mg, 1.0 equiv, 0.20 mmol), sodium fluoride (25 mg, 3.0 equiv, 0.60 mmol), chloro(crotyl)(tri*tert*-butylphosphine)palladium(II) (4 mg, 5 mol %, 10.0 μmol), tri*tert*-butylphosphoniumtetrafluoroborate (3 mg, 5 mol %, 10.0 μmol), **4e** (94 mg, 2.12 equiv, 0.42 mmol), dry DMSO (0.70 mL), and triethylamine (4.2 μL, 15 mol %, 30.0 μmol). The material was purified by flash column chromatography (10–100% MeCN/H_2_O, 0.1% v/v TFA, C_18_), followed by a second column purification (40% EtOAc/hexanes, SiO_2_). The product-containing fractions were aggregated and concentrated in vacuo, affording **6e** as a fine, amorphous yellow solid (68 mg, 0.18 mmol, 91% yield). ^1^H NMR indicated a >20:1 *E*/*Z* of the alkene. ^1^H NMR (600 MHz, CDCl_3_): δ 8.12 (s, 1H), 8.02 (s, 1H), 7.84 (d, *J* = 7.9 Hz, 1*H*), 7.79 (dd, *J* = 7.8, 1.5 Hz, 1H), 7.45 (d, *J* = 8.1 Hz, 2H), 7.29 (d, *J* = 16.3 Hz, 1H), 7.21 (d, *J* = 7.8 Hz, 2H), 7.13 (d, *J* = 16.2 Hz, 1H), 5.00 (dd, *J* = 12.5, 5.4 Hz, 1H), 3.01–2.80 (m, 3H), 2.80–2.68 (m, 1H), 2.38 (s, 3H), 2.17 (dtd, *J* = 12.6, 5.0, 2.2 Hz, 1H). ^13^C{^1^H} NMR (101 MHz, CDCl_3_): δ 171.0, 168.1, 167.4, 167.2, 144.6, 139.3, 133.4, 132.7, 132.4, 129.8, 129.6, 127.1, 125.5, 124.4, 120.9, 49.5, 31.6, 22.8, 21.5. HRMS (APCI) *m*/*z*: 375.1336 ([M + H]^+^ req 375.1339) calcd for C_22_H_19_O_4_N_2_. FTIR (neat) ν_max_/cm^–1^: 3467, 3207, 3090, 2918, 1772, 1699, 1604.

#### 2-(2,6-Dioxopiperidin-3-yl)-5-phenylisoindoline-1,3-dione (**6f**)

Compound **6f** was prepared via General Procedure **B-2** using **1a** (67 mg, 1.0 equiv, 0.20 mmol), sodium fluoride (25 mg, 3.0 equiv, 0.60 mmol), chloro(crotyl)(tri*tert*-butylphosphine)palladium(II) (4 mg, 5 mol %, 10.0 μmol), tri*tert*-butylphosphoniumtetrafluoroborate (3 mg, 5 mol %, 10.0 μmol), **4f** (94 mg, 2.12 equiv, 0.42 mmol), dry DMSO (0.70 mL), and triethylamine (4.2 μL, 15 mol %, 30.0 μmol). The material was purified by flash column chromatography (10–100% MeCN/H_2_O, 0.1% v/v TFA, C_18_), followed by a second column purification (40% EtOAc/hexanes, SiO_2_). The product-containing fractions were aggregated and concentrated in vacuo, affording **6f** as a fine, amorphous yellow solid (17 mg, 0.05 mmol, 26% yield). ^1^H NMR (600 MHz, DMSO-*d*_6_): δ 11.16 (s, 1H), 8.21–8.14 (m, 2H), 8.01 (d, *J* = 8.2 Hz, 1H), 7.85 (d, *J* = 7.3 Hz, 2H), 7.54 (t, *J* = 7.5 Hz, 2H), 7.49 (t, *J* = 7.3 Hz, 1H), 5.19 (dd, *J* = 12.8, 5.5 Hz, 1H), 2.91 (ddd, *J* = 16.7, 13.8, 5.4 Hz, 1H), 2.68–2.54 (m, 2H), 2.13–2.00 (m, 1H). Spectral data were consistent with the literature.^9c^

#### 2-(2,6-Dioxopiperidin-3-yl)-5-(2-methoxypyrimidin-5-yl)isoindoline-1,3-dione (**6g**)

Compound **6g** was prepared via General Procedure **B-2** using **1a** (67 mg, 1.0 equiv, 0.20 mmol), sodium fluoride (25 mg, 3.0 equiv, 0.60 mmol), chloro(crotyl)(tri*tert*-butylphosphine)palladium(II) (4 mg, 5 mol %, 10.0 μmol), tri*tert*-butylphosphoniumtetrafluoroborate (3 mg, 5 mol %, 10.0 μmol), **4g** (91 mg, 2.12 equiv, 0.42 mmol), dry DMSO (0.70 mL), and triethylamine (4.2 μL, 15 mol %, 30.0 μmol). The material was purified by flash column chromatography (10–100% MeCN/H_2_O, 0.1% v/v TFA, C_18_), followed by a second column purification (70–100% EtOAc/hexanes, SiO_2_). The product-containing fractions were aggregated and concentrated in vacuo, affording **6g** as a fine, amorphous white solid (37 mg, 0.10 mmol, 50% yield). ^1^H NMR (600 MHz, DMSO-*d*_6_): δ 11.16 (s, 1H), 9.12 (s, 2H), 8.33 (d, *J* = 1.6 Hz, 1H), 8.25 (dd, *J* = 7.8, 1.5 Hz, 1H), 8.03 (d, *J* = 7.8 Hz, 1H), 5.20 (dd, *J* = 12.8, 5.4 Hz, 1H), 3.99 (s, 3H), 2.91 (ddd, *J* = 16.9, 13.7, 5.4 Hz, 1H), 2.66–2.54 (m, 2H), 2.14–2.04 (m, 1H). ^13^C{^1^H} NMR (101 MHz, DMSO-*d*_6_): δ 172.8, 169.8, 166.9, 166.8, 165.2, 158.2, 140.6, 132.5, 132.4, 130.3, 125.7, 124.1, 121.3, 55.0, 49.1, 31.0, 22.0. HRMS (APCI) *m*/*z*: [M + H]^+^ calcd for C_18_H_15_O_5_N_4_, 367.1037; found, 367.1035. FTIR (neat) ν_max_/cm^–1^: 3468, 3233, 2918, 1775, 1702, 1595, 1471, 1411, 1393, 1327, 1116, 1028, 910, 730, 610.

#### 2-(2,6-Dioxopiperidin-3-yl)-5-(thiophen-2-yl)isoindoline-1,3-dione (**6h**)

Compound **6h** was prepared via General Procedure **B-2** using **1a** (169 mg, 1.0 equiv, 0.50 mmol), sodium fluoride (63 mg, 3 equiv, 1.50 mmol), chloro(crotyl)(tri*tert*-butylphosphine)palladium(II) (10 mg, 5 mol %, 25.0 μmol), tri*tert*-butylphosphoniumtetrafluoroborate (7 mg, 5 mol %, 25.0 μmol), **4h** (201 mg, 2.12 equiv, 1.06 mmol), dry DMSO (1.71 mL), and triethylamine (10.5 μL, 15 mol %, 75.0 μmol). The material was purified by flash column chromatography (40–70% EtOAc/hexanes, SiO_2_), followed by a second column purification (10–100% MeCN/H_2_O, 0.1% v/v TFA, C_18_), which afforded **6h** (137 mg, 0.40 mmol, 80% yield) as a fine, amorphous yellow solid. ^1^H NMR (600 MHz, DMSO-*d*_6_): δ 11.13 (s, 1H), 8.16 (s, 1H), 8.10 (d, *J* = 7.9 Hz, 1H), 7.91 (d, *J* = 7.8 Hz, 1H), 7.89 (d, *J* = 3.8 Hz, 1H), 7.73 (d, *J* = 5.1 Hz, 1H), 7.22–7.15 (m, 1H), 5.15 (dd, *J* = 12.9, 5.5 Hz, 1H), 2.87 (ddd, *J* = 18.1, 13.7, 5.5 Hz, 1H), 2.61–2.48 (m, 2H), 2.10–1.95 (m, 1H). ^13^C{^1^H} NMR (101 MHz, DMSO): δ 174.1, 171.1, 168.1, 168.0, 142.3, 141.2, 133.9, 132.2, 130.5, 130.4, 129.8, 128.2, 125.7, 120.8, 50.3, 32.2, 23.3. HRMS (APCI) *m*/*z*: [M + H]^+^ calcd for C_17_H_14_O_4_N_2_^32^S, 341.0591; found, 341.0591. FTIR (neat) ν_max_/cm^–1^: 3470, 3191, 3088, 2896, 1770, 1703, 1616.

#### 2-(2,6-Dioxopiperidin-3-yl)-5-(1-methyl-1*H*-pyrazol-5-yl)isoindoline-1,3-dione (**6i**)

Compound **6i** was prepared via General Procedure **B-2** using **1a** (169 mg, 1.0 equiv, 0.50 mmol), sodium fluoride (63 mg, 3 equiv, 1.50 mmol), chloro(crotyl)(tri*tert*-butylphosphine)palladium(II) (10 mg, 5 mol %, 25.0 μmol), tri*tert*-butylphosphoniumtetrafluoroborate (7 mg, 5 mol %, 25.0 μmol), **4i** (199 mg, 2.12 equiv, 1.06 mmol), dry DMSO (1.71 mL), and triethylamine (10.5 μL, 15 mol %, 75.0 μmol). The material was purified by flash column chromatography (50–100% EtOAc/hexanes, SiO_2_). The product-containing fractions were aggregated and concentrated in vacuo, affording **6i** (127 mg, 0.38 mmol, 75% yield) as a fine amorphous yellow solid. ^1^H NMR (400 MHz, DMSO): δ 11.16 (s, 1H), 8.16–7.87 (m, 3H), 7.55 (d, *J* = 1.9 Hz, 1H), 6.66 (d, *J* = 1.9 Hz, 1H), 5.20 (dd, *J* = 13.0, 5.3 Hz, 1H), 3.94 (s, 3H), 2.91 (ddd, *J* = 17.4, 14.0, 5.4 Hz, 1H), 2.66–2.53 (m, 2H), 2.14–2.02 (m, 1H). ^13^C{^1^H} NMR (101 MHz, DMSO): δ 172.8, 169.8, 166.7, 166.7, 140.9, 138.2, 136.3, 134.5, 132.0, 130.4, 123.9, 122.9, 107.3, 49.1, 37.9, 30.9, 22.0. HRMS (APCI) *m*/*z*: [M + H]^+^ calcd for C_17_H_15_O_4_N_4_, 339.1088; found, 339.1086. FTIR (neat) ν_max_/cm^–1^: 3076, 1712, 1379, 1202.

#### Methyl 4-(2-(2,6-Dioxopiperidin-3-yl)-1,3-dioxoisoindolin-5-yl)-1-methyl-1*H*-pyrazole-3-carboxylate (**6j**)

Compound **6j** was prepared via General Procedure **B-2** using **1a** (169 mg, 1.0 equiv, 0.50 mmol), sodium fluoride (63 mg, 3 equiv, 1.50 mmol), chloro(crotyl)(tri*tert*-butylphosphine)palladium(II) (10 mg, 5 mol %, 25.0 μmol), tri*tert*-butylphosphoniumtetrafluoroborate (7 mg, 5 mol %, 25.0 μmol), **4j** (261 mg, 2.12 equiv, 1.06 mmol), dry DMSO (1.71 mL), and triethylamine (10.5 μL, 15 mol %, 75.0 μmol). The material was purified by flash column chromatography (50–100% EtOAc/hexanes à 5% EtOH/EtOAC, SiO_2_). The product-containing fractions were aggregated and concentrated in vacuo, affording **6j** (172 mg, 0.43 mmol, 87% yield) as a fine amorphous yellow solid. ^1^H NMR (600 MHz, DMSO-*d*_6_): δ 11.15 (s, 1H), 8.29 (s, 1H), 8.03 (s, 1H), 7.98–7.82 (m, 2H), 5.17 (dd, *J* = 12.9, 5.4 Hz, 1H), 3.97 (s, 3H), 3.77 (s, 3H), 2.91 (ddd, *J* = 17.3, 14.0, 5.5 Hz, 1H), 2.67–2.53 (m, 2H), 2.16–2.04 (m, 1H). ^13^C{^1^H} NMR (101 MHz, DMSO-*d*_6_): δ 172.8, 169.9, 167.1, 166.9, 162.4, 138.4, 138.2, 134.8, 133.2, 131.4, 129.4, 123.5, 123.2, 123.0, 51.6, 49.1, 31.0, 22.0. HRMS (APCI) *m*/*z*: [M + H]^+^ calcd for C_19_H_17_O_6_N_4_, 397.1143; found, 397.1142. FTIR (neat) ν_max_/cm^–1^: 3195, 2957, 1774, 1698, 1620, 1557, 1698, 1411, 1198, 1377, 639.

#### 5-(3,5-Dimethylisoxazol-4-yl)-2-(2,6-dioxopiperidin-3-yl)isoindoline-1,3-dione (**6k**)

Compound **6k** was prepared via General Procedure **B-2** using **1a** (169 mg, 1.0 equiv, 0.50 mmol), sodium fluoride (63 mg, 3 equiv, 1.50 mmol), chloro(crotyl)(tri*tert*-butylphosphine)palladium(II) (10 mg, 5 mol %, 25.0 μmol), tri*tert*-butylphosphoniumtetrafluoroborate (7 mg, 5 mol %, 25.0 μmol), **4k** (215 mg, 2.12 equiv, 1.06 mmol), dry DMSO (1.71 mL), and triethylamine (10.5 μL, 15 mol %, 75.0 μmol). The material was purified by flash column chromatography (10–100% MeCN/H_2_O, 0.1% v/v TFA, C_18_), followed by a second column purification (50–100% EtOAc/hexanes, SiO_2_). The product-containing fractions were aggregated and concentrated in vacuo, affording **6k** (151 mg, 0.43 mmol, 86% yield) as a fine amorphous yellow solid. ^1^H NMR (600 MHz, DMSO-*d*_6_): δ 11.16 (s, 1H), 8.02 (d, *J* = 7.7 Hz, 1H), 7.96 (s, 1H), 7.90 (dd, *J* = 7.7, 1.5 Hz, 1H), 5.19 (dd, *J* = 12.9, 5.4 Hz, 1H), 2.91 (ddd, *J* = 16.9, 13.9, 5.4 Hz, 1H), 2.67–2.53 (m, 2H), 2.46 (s, 3H), 2.28 (s, 3H), 2.15–2.01 (m, 1H). ^13^C NMR{^1^H} (101 MHz, DMSO-*d*_6_): δ 172.9, 169.9, 166.9, 166.9, 166.6, 158.1, 136.8, 135.2, 132.2, 130.0, 124.0, 123.6, 115.0, 49.1, 31.0, 22.0, 11.5, 10.4. HRMS (APCI) *m*/*z*: [M + H]^+^ calcd for C_18_H_16_O_5_N_3_, 354.1085; found, 354.1084. FTIR (neat) ν_max_/cm^–1^: 3213, 3109, 2917, 1776, 1708, 1626, 1380, 1260, 1195.

#### 2-(2,6-Dioxopiperidin-3-yl)-5-(furan-2-yl)isoindoline-1,3-dione (**6**l)

Compound **6l** was prepared via General Procedure **B-2** using **1a** (67 mg, 1.0 equiv, 0.20 mmol), sodium fluoride (25 mg, 3.0 equiv, 0.60 mmol), chloro(crotyl)(tri*tert*-butylphosphine)palladium(II) (4 mg, 5 mol %, 10.0 μmol), tri*tert*-butylphosphoniumtetrafluoroborate (3 mg, 5 mol %, 10.0 μmol), **4l** (74 mg, 2.12 equiv, 0.42 mmol), dry DMSO (0.70 mL), and triethylamine (4.2 μL, 15 mol %, 30.0 μmol). The material was purified by flash column chromatography (10–100% MeCN/H_2_O, 0.1% v/v TFA, C_18_). The product-containing fractions were aggregated and concentrated in vacuo, affording **6l** as a fine, amorphous tan solid (53 mg, 0.16 mmol, 82% yield). ^1^H NMR (600 MHz, DMSO-*d*_6_): δ 11.15 (s, 1H), 8.21 (s, 1H), 8.16 (dd, *J* = 7.8, 1.5 Hz, 1H), 7.97 (d, *J* = 7.9 Hz, 1H), 7.92 (d, *J* = 1.6 Hz, 1H), 7.44 (d, *J* = 1.4 Hz, 1H), 6.72 (dd, *J* = 3.4, 1.7 Hz, 1H), 5.18 (dd, *J* = 12.9, 5.4 Hz, 1H), 2.90 (ddd, *J* = 17.1, 14.2, 5.4 Hz, 1H), 2.65–2.52 (m, 2H), 2.13–2.03 (m, 1H). ^13^C{^1^H} NMR (101 MHz, DMSO-*d*_6_): δ 172.8, 169.9, 166.8, 166.8, 151.2, 145.0, 136.0, 132.5, 129.0, 128.6, 124.3, 117.8, 112.9, 110.1, 49.1, 30.9, 22.0. HRMS (APCI) *m*/*z*: [M + H]^+^ calcd for C_1__7_H_1__3_O_5_N_2_, 325.0819; found, 325.0816. FTIR (neat) ν_max_/cm^–1^: 3469, 3211, 3143, 3110, 3043, 2858, 1776, 1706, 1619.

## Data Availability

The data underlying this study are available in the published article and its online Supporting Information.

## Supporting Information Available

Optimization studies, additional additive studies, expanded studies of organoboron compounds, compound characterization, including NMR spectra and chromatograms, and computational details (PDF)
