# Supplementary material for: Anhydrous and Stereoretentive Fluoride-Enhanced Suzuki–Miyaura Coupling of Immunomodulatory Imide Drug Derivatives
Source: J Org Chem. 2024 Mar 7;89(7):4595–606. doi: 10.1021/acs.joc.3c02873 (PMC11002932; doi:10.1021/acs.joc.3c02873)
Supplement: Supplementary file 1 — jo3c02873_si_001.pdf [file jo3c02873_si_001.pdf]

Anhydrous and Stereoretentive Fluoride-Enhanced Suzuki-Miyaura Coupling of Immunomodulatory Imide Drug Derivatives.

Supporting Information
(67 Pages)

William F. Tracy,[a] Geraint H. M. Davies,[b] Lauren N. Grant,[c] Jacob M. Ganley,[c] Jesus Moreno,[d,*] Emily C. Cherney[e,*] and Huw M. L. Davies[a,*]

[a] Department of Chemistry, Emory University, Atlanta, Georgia 30322, United States

[b] Small Molecule Drug Discovery, Bristol Myers Squibb, Cambridge, Massachusetts 02140, United States

[c] Chemical Process Development, Bristol Myers Squibb, New Brunswick, New Jersey 08903, United States

[d] Small Molecule Drug Discovery, Bristol Myers Squibb, San Diego, California 92121, United States

[e] Small Molecule Drug Discovery, Bristol Myers Squibb, Princeton, New Jersey 08543, United States

*Corresponding author: hmdavie@emory.edu

Table of Contents

Section 1: Supplemental Reaction Information……………………………………………..……S3

Scheme S1: Detailed optimization study ……………………………….……………..….S3

Scheme S2: Screen of basic additives……………………………..……….....…..….S4

Scheme S3: Screen of organoboron nucleophiles……………………….………...….S4

Scheme S4: Limitations of the substrate scope……………………………...…….….S5

Scheme S5: Attempts at generation of 2a using literature and patent conditions……….S6

Section 2: General Information…………………………………………………………….….S7

Figure S1: Aryl halides used in this study………………………………...…………S7

Figure S2: Potassium trifluoroborates used in this study………………………......…..S7

Section 4: Computational Details……………………………………….….…..………………..S8

Potassium (vinyl)trifluoroborate Association Complexes…………………….....………..S8

Potassium (vinyl)trifluoroborate π-complexes……………………….……………...S14

Transmetalation Structures **III-V**…………………….…………………….…..S20

Potassium (phenyl)trifluoroborate π-complexes…………………….…………….....S26

Potassium (phenyl)trifluoroborate Association Complexes……………………..…..S32

Heterocyclic trifluoroborate π-complexes……………...…………….…………...S38

Section 5: Spectroscopic Data S42

Section 5A: ^{1}H NMR Spectra……………………………………...…………S42

Section 5B: ^{13}C{^{1}H} NMR Spectra……………………………………………..S55

Section 6: Chromatographic Data …………….. S67

Section 7: References……………………………………………………...…..…S69

Section 1. Supplemental Reaction Information

Scheme S1: Detailed reaction optimization

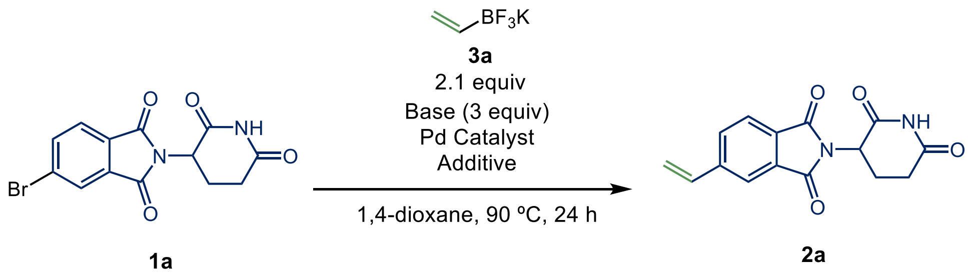

Entry	Base	Catalyst	Additive	% Yield 2a[1]
1	KF	$Pd(dppf)_2Cl_2$ (10 mol%)	none	44
2	CsF	"	"	45
3	LiF	"	"	41
4	AgF	"	"	50
5	NaF	"	"	53
6	NaF	$Pd(PPh_3)_2Cl_2$ (10 mol%)	"	50
7	"	$Pd(amphos)_2Cl_2$ (10 mol%)	"	55
8	"	dppf Pd G4 (10 mol%)	"	19
9	"	$Pd(P^tBu_3)_2$ (10 mol%)	"	82
10	"	P^tBu_3 Pd G4 (5 mol%)	$^iPr_2NH$ (10 mol%)	70
11	"	P^tBu_3 Pd G4 (5 mol%)	Et_3N (10 mol%)	74
12	"	P^tBu_3 Pd(crotyl)Cl (5 mol%)	"	78
13	"	"	Et_3N (15 mol%)<br>$P^tBu_3 \cdot HBF_4$ (5 mol%)	93

[1] Isolated yield of purified product

Scheme S2: Screen of basic additives

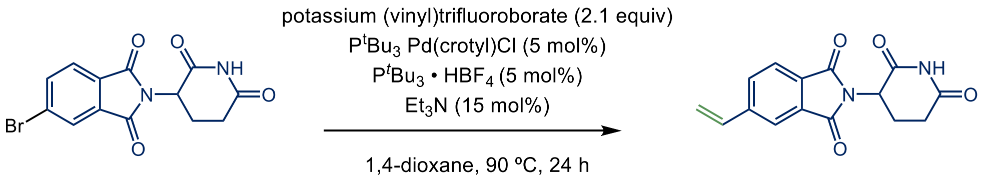

1a **2a**

Entry	Base	% Yield 2a [1]	Entry	Base	% Yield 2a [1]
**1**	NaF	93	**7**	NaOH	49
**2**	Cs_2CO_3	22	**8**	$NaHCO_3$	47
**3**[2]	TBAF	39	**9**	NaOAc	63
**4**	K_3PO_4	63	**10**	NaOTf	65
**5**	TMSOK	n.d.	**11**	NaTFA	74
**6**	^{t}BuOK	n.d.			

[1] Isolated yield of purified product [2] Commerical TBAF (Millipore Sigma, 1.0 M in THF, 216143) contains ca. 5 wt% H_2O

Scheme S3: Screen of organoboron nucleophiles

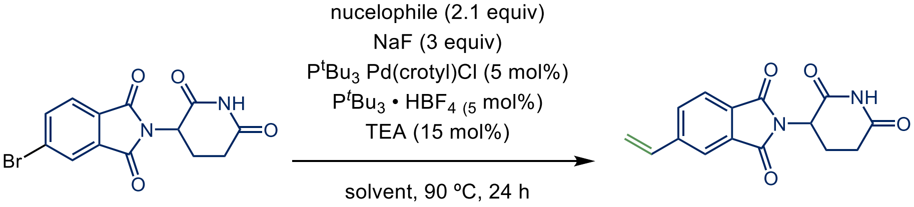

1a **2a**

Entry	Nucleophile	Solvent	% Yield 2a [1]
**1**	potassium vinyltrifluoroborate	1,4-dioxane	93
**2**	vinylboronic acid MIDA ester	"	trace
**3**	vinylboronic acid pinacol ester	"	28
**4**	potassium phenyltrifluoroborate	"	45
**5**	"	DMSO	26
**6**	phenylboronic acid	1,4-dioxane	26
**7** [2]	"	"	29
**8**	"	DMSO	57
**9**	phenylboronic acid pinacol ester	1,4-dioxane	trace
**10**	"	DMSO	trace

[1] Isolated yield of purified product [2] 6 equiv NaF

Scheme S4: Limitations of the substrate scope.

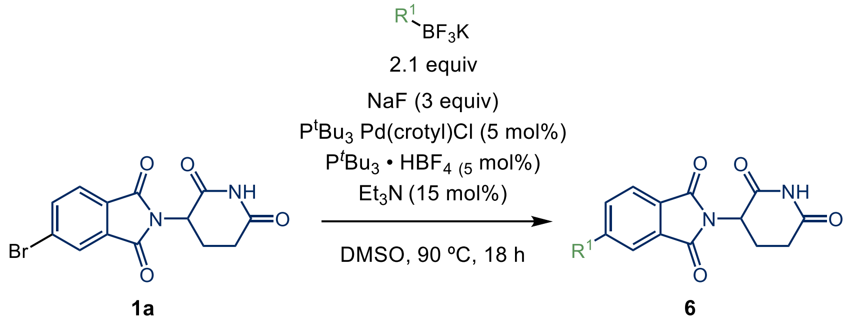

Recovered ArBr

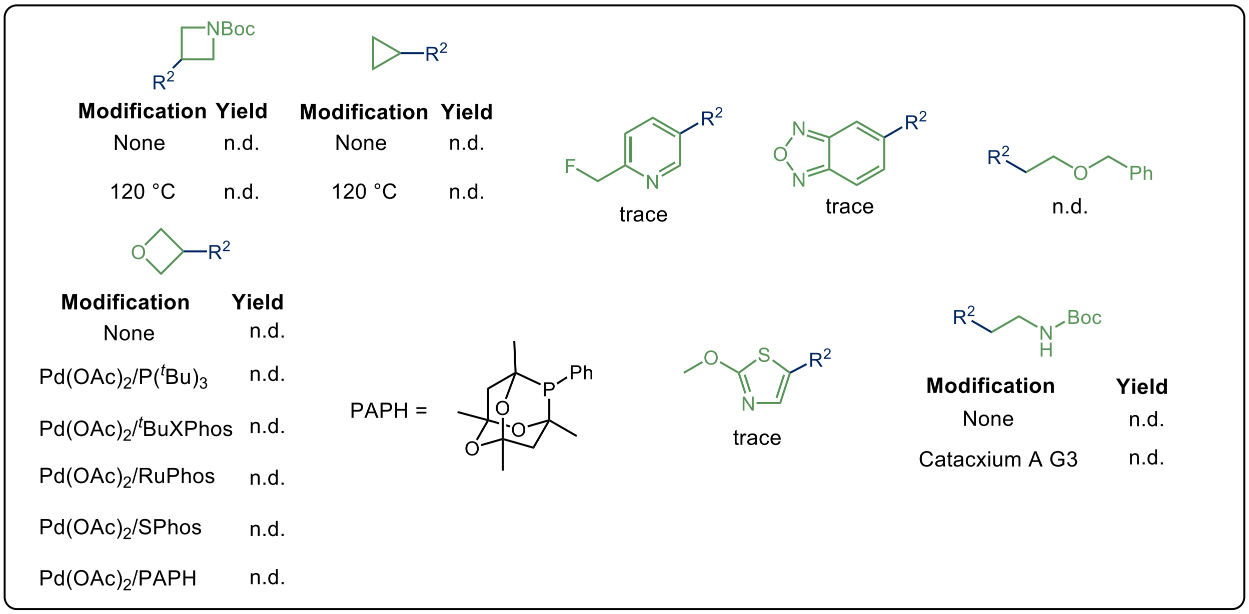

Protodehalogenation as major product

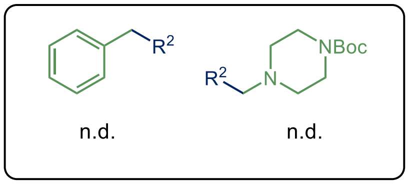

Complex mixture

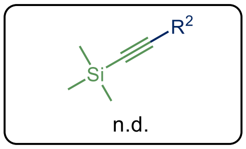

Scheme S5: Attempts at generation of 2a using literature and patent conditions.

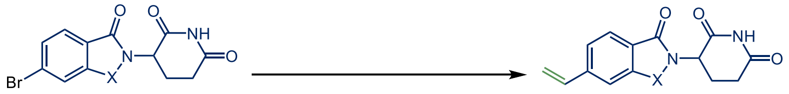

X = CH_2, CO
1

2a *(X = CO)*
2c *(X = CH_2)*

Entry	Conditions	Vinyl source	Result	Reference
**1**	$PdCl_2$(dppf) (10 mol%), Cs_2CO_3 (2 equiv), 1,4-dioxane, 90 °C, 18 h	BF_3K 3.0 equiv	33% yield **2a**	Lu, L., et al. US 0083376 A1, **2021**.
**2**	$PdCl_2$(dppf) (10 mol%), NaOH (1 equiv), *N*-cyclohexyl-*N*-methylcyclohexanamine (1 equiv), THF, 67 °C, 18 h	BPin 1.2 equiv	trace **2a**	Stewart, S.G., et al. *Biorg. Med. Chem.* **2010**, 18, 650-662
**3**	$Pd_2(dba)_3$ (0.5 mol%), PAPh (1.5 mol%), K_2CO_3 (2.5 equiv), 1,4-dioxane:H_2O (4:1), 80 °C, 18h	BPin 1.5 equiv	11% yield **2a**	Sharland, J. C., et al. . *Chem. Sci.* **2021**, 12, 11181-11190.
**4**	$NiCl_2$(dppp) (10 mol%), LiI (3.5 equiv), Zn (2 equiv), dimethyl isosorbide, 65 °C, 8 h	O O 2 equiv	trace **2a**	Su, M., et al. *Org. Lett.* **2022**, 24, 354-358.
**5**	$PdCl_2(amphos)_2$ (2 mol%), 2.34 equiv [(tmeda)Zn(OH)(OTf)]$_3$, 1,4-dioxane, 80 °C, 3 h	BF_3K 1.1 equiv	85% yield **2a** 42% yield **2c**	Niwa, T., et al. *Nature Catalysis* **2021**, 4, 1080-1088
**6**	This Work	BF_3K 2.1 equiv	High yield for all derivatives	

Section 2. General Information

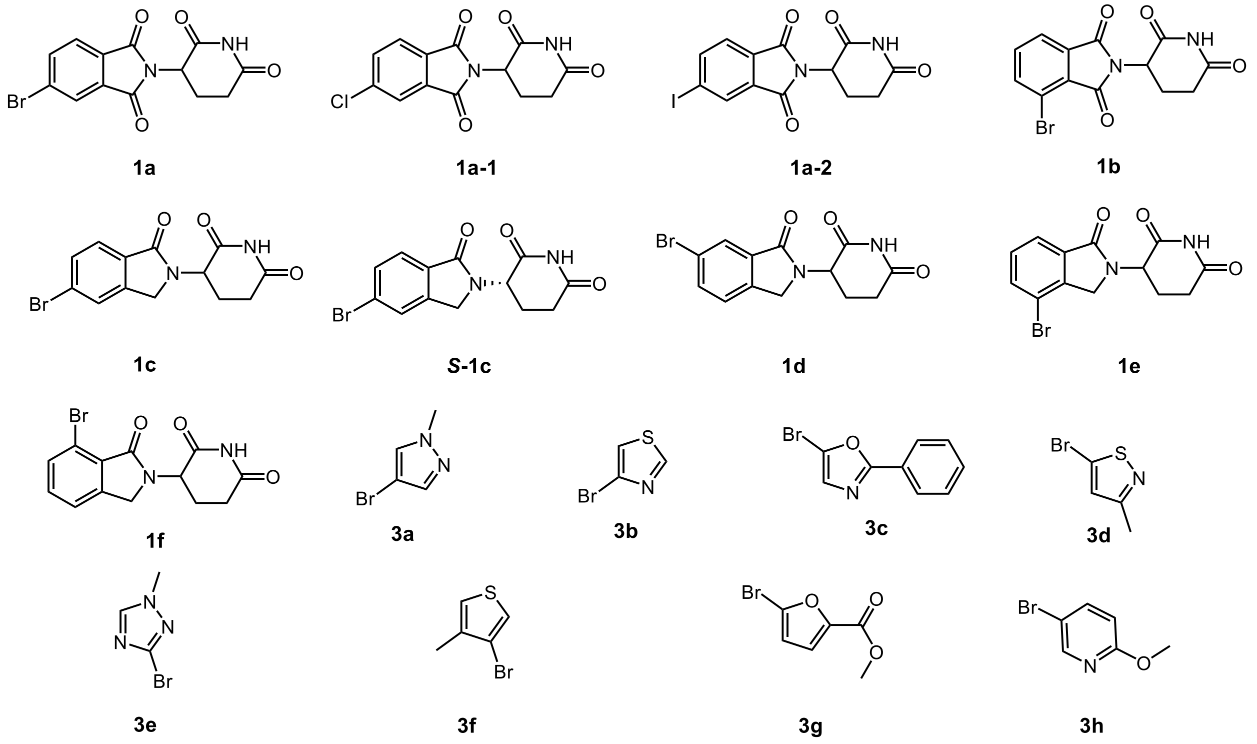

Figure S1: Aryl halides used in this study

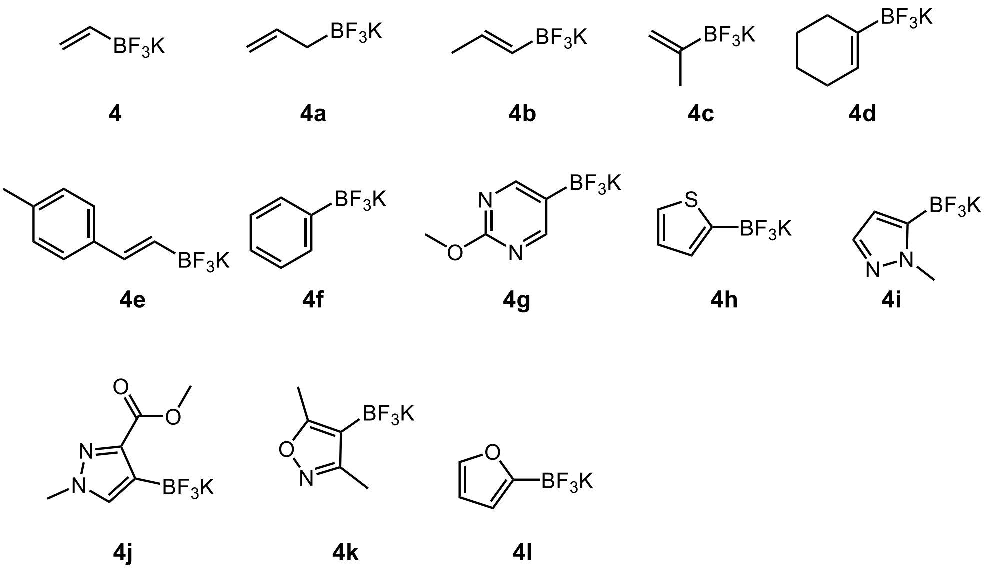

Figure S2: Trifluoroborates used in this study

Section 4: Computation Details

All DFT geometry optimization and frequency calculations were conducted with Gaussian 16, revision C.01.[1] Geometry optimizations were performed in the gas phase with the M06 functional and 6-311G++(d,p) basis sets for all atoms, excluding Pd and Br. Pd was computed with a Stuttgart 1997 (with ECP) basis set and LANL2DZ (with ECP for Br). All stationary points were characterized by frequency calculations to confirm local minima for ground state optimizations (zero imaginary frequencies). All energy values are reported in Hartrees and are the sum of electronic and thermal free energies as computed by Gaussian16.

Potassium (vinyl)trifluoroborate Association Complexes

Three fluoride associated complexes were considered in this study (**S1**, **S2** and **I**). Structure **I** was found to be the most stable and the further calculations began from that structure.:

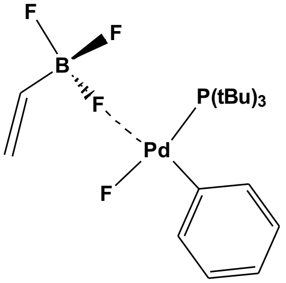

Isomer **S1** arrangement: Borate and phenyl are transoid Fluoride and phosphine are transoid

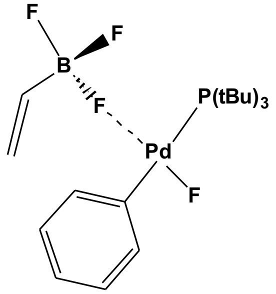

Isomer **S2** arrangement: Borate and phenyl are cisoid Phenyl and phosphine are transoid

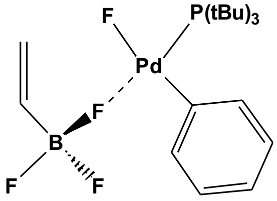

Isomer **I** arrangement: Borate and phenyl are transoid Borate and phosphine are transoid

Cartesian Coordinates:

$((tBu)_3P)Pd(Ph)(F)(BF_3(CH{=}CH_2))$ association anion isomer **I**

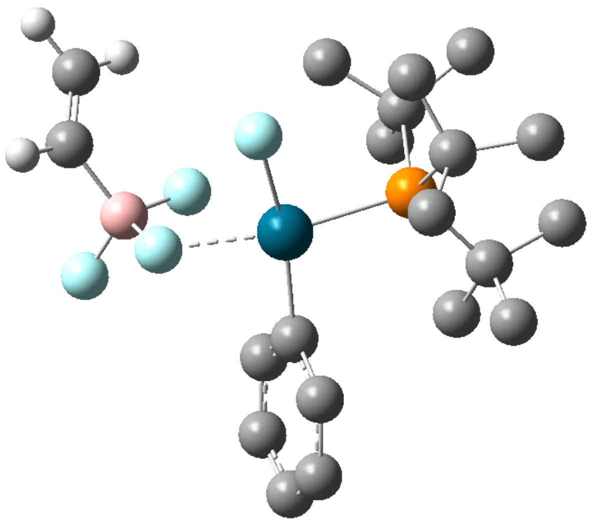

Cartesian coordinates for association anion isomer **I**

Sum of electronic and thermal free energies: -1675.663058

Pd	-0.59213100	-0.15188900	-0.74325200
F	-2.76256200	-0.39422500	-1.01893300
B	-3.45091700	-0.95243900	0.17145600
F	-4.45365600	-0.05164400	0.49386100
F	-2.48961100	-0.98135500	1.21051000
C	-3.98692700	-2.41703700	-0.18735100
C	-3.70076500	-3.52155000	0.50293400
C	-1.00385200	1.73833800	-0.27781800
C	-0.64547000	2.74756800	-1.17886100
C	-1.05884400	4.06661600	-0.98202300
C	-1.84687100	4.39292700	0.11717400
C	-2.24139200	3.38589700	0.99714800
C	-1.83079700	2.06990200	0.80115800
P	1.55400000	-0.21499700	0.10836100
C	2.40853100	1.37317700	0.82467800
C	3.64962500	1.08635700	1.67908400
C	1.44852900	2.20106600	1.69377600
C	2.80903000	2.30878100	-0.32194300
C	2.72568100	-0.89751700	-1.27103600
C	2.42066500	-2.37343500	-1.55685300
C	4.22948700	-0.78505500	-1.00770800
C	2.37011900	-0.13797400	-2.55715600
C	1.45641200	-1.50708500	1.53593600
C	2.79677000	-2.14156100	1.91842200
C	0.47328000	-2.62092500	1.14492100
C	0.85413500	-0.85039600	2.78185500
H	-4.64998900	-2.52904300	-1.05452000
H	-4.08365000	-4.51276000	0.24331100
H	-3.04243700	-3.48154400	1.37399600

H	-0.03670000	2.50326700	-2.05377800
H	-0.76393700	4.83942100	-1.69422000
H	-2.16761200	5.42255000	0.27837600
H	-2.88758400	3.62534700	1.84290100
H	-2.17267500	1.28256900	1.47266700
H	4.38892000	0.44187200	1.19374000
H	4.14540500	2.04577900	1.89846100
H	3.38548200	0.63935300	2.64550900
H	0.69964200	2.71810600	1.08981200
H	2.04671300	2.96621400	2.21518000
H	0.91633200	1.62436000	2.45350400
H	3.62849000	1.92792300	-0.94086500
H	1.95210900	2.54086900	-0.96639600
H	3.14941100	3.26006700	0.11756300
H	2.76752900	-3.04106900	-0.75835400
H	1.34429600	-2.51792200	-1.72819500
H	2.97263000	-2.65444000	-2.46898100
H	4.59598100	0.24494000	-0.92911100
H	4.75554700	-1.24694700	-1.85854600
H	4.53851300	-1.33186300	-0.10679600
H	1.32024300	-0.31470900	-2.82619100
H	2.53146500	0.94378900	-2.48793400
H	3.00351900	-0.51660500	-3.37580000
H	3.55218500	-1.41498200	2.23835000
H	2.62405700	-2.82441000	2.76561500
H	3.22212200	-2.74294200	1.10682500
H	-0.55170200	-2.23929700	1.05929900
H	0.49374100	-3.37381800	1.95041900
H	0.70521500	-3.11412000	0.19940000
H	1.53316500	-0.14416600	3.27646600
H	-0.09601000	-0.34877600	2.55322800
H	0.63030100	-1.64680200	3.50851300
F	-0.43108100	-1.99107800	-1.62595000

$((tBu)_3P)Pd(Ph)(F)(BF_3(CH{=}CH_2))$ association anion isomer **S2**

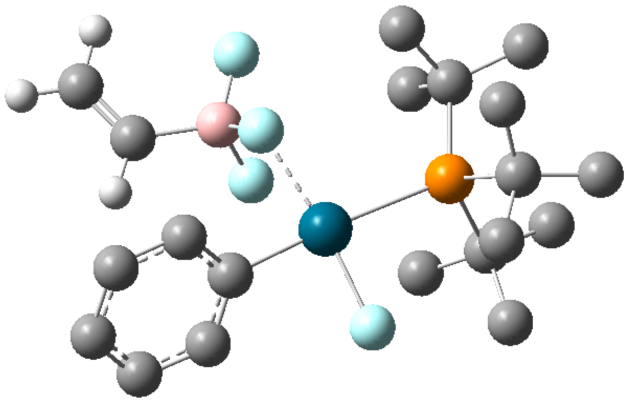

Cartesian coordinates for association anion isomer **S2**

Sum of electronic and thermal free energies: -1675.665091

Pd	0.59642600	-0.64176100	-0.16066600
F	1.31645500	1.28015400	-0.78337500
B	1.81471800	2.22924900	0.28880500
F	1.33008600	1.68775900	1.48596700
F	1.19062500	3.43361500	0.01241400
C	3.40744100	2.27775200	0.21986600
C	4.10331000	3.37613400	-0.07654700
C	2.50883500	-1.19968100	-0.17459600
C	3.32089500	-0.89719900	-1.26706400
C	4.67465600	-1.23347700	-1.24868200
C	5.22721100	-1.85491900	-0.13224900
C	4.41589700	-2.15300900	0.96048300
C	3.05789000	-1.83961400	0.93525300
P	-1.86194500	-0.06777500	-0.06100300
C	-2.45854600	-0.63031600	1.68248100
C	-3.81285100	-0.09247300	2.14491300
C	-1.36940500	-0.20005400	2.67736200
C	-2.49912800	-2.16159800	1.75739100
C	-2.83710100	-1.07536900	-1.37889700
C	-2.67832700	-0.41237200	-2.75034000
C	-4.33115700	-1.26307900	-1.11131000
C	-2.16916500	-2.44968600	-1.51674300
C	-2.33903800	1.78748500	-0.27633200
C	-3.82545100	2.08948800	-0.47451500
C	-1.56412300	2.35393300	-1.47512000
C	-1.83862100	2.56799800	0.94520800
H	3.96646000	1.35803500	0.43464200
H	5.19569200	3.40663600	-0.11509900
H	3.59282400	4.31645600	-0.29764200
H	2.90384900	-0.37285600	-2.12768800
H	5.30315800	-0.99133900	-2.10718300
H	6.28860300	-2.10460000	-0.11218500
H	4.84260300	-2.63837700	1.83998200
H	2.41782200	-2.09007800	1.78130400
H	-4.63082400	-0.37010400	1.46663300
H	-4.04488300	-0.52854300	3.13075800
H	-3.82422200	0.99724800	2.26701000
H	-0.40340700	-0.65836700	2.42460000
H	-1.65987900	-0.54476100	3.68370000
H	-1.21298300	0.88110900	2.72577200
H	-3.33599500	-2.59753600	1.19655700
H	-1.55203400	-2.59247900	1.40643200
H	-2.64334400	-2.44390200	2.81333400

H	-3.26109200	0.51046000	-2.85516800
H	-1.62555600	-0.19249500	-2.97624800
H	-3.04188900	-1.11360300	-3.51871500
H	-4.52487800	-1.85521200	-0.20909200
H	-4.78041900	-1.80953800	-1.95756900
H	-4.86863600	-0.31147400	-1.01448700
H	-1.12174700	-2.34894400	-1.82778000
H	-2.16185200	-3.03444900	-0.59523200
H	-2.71229500	-3.02196800	-2.28772400
H	-4.45676100	1.71646500	0.34130300
H	-3.95668400	3.18339000	-0.51764300
H	-4.21443000	1.68606500	-1.41850200
H	-0.48925000	2.17260700	-1.38260600
H	-1.71036900	3.44568200	-1.49391600
H	-1.91083500	1.96504200	-2.43700600
H	-2.42984100	2.37119700	1.84787800
H	-0.77919800	2.37814400	1.15723300
H	-1.93000400	3.64340600	0.72639600
F	0.18871600	-2.50814800	0.42124400

(($(tBu)_3$P)Pd(Ph)(F)($BF_3(CH=CH_2)$)) association anion isomer **S1**

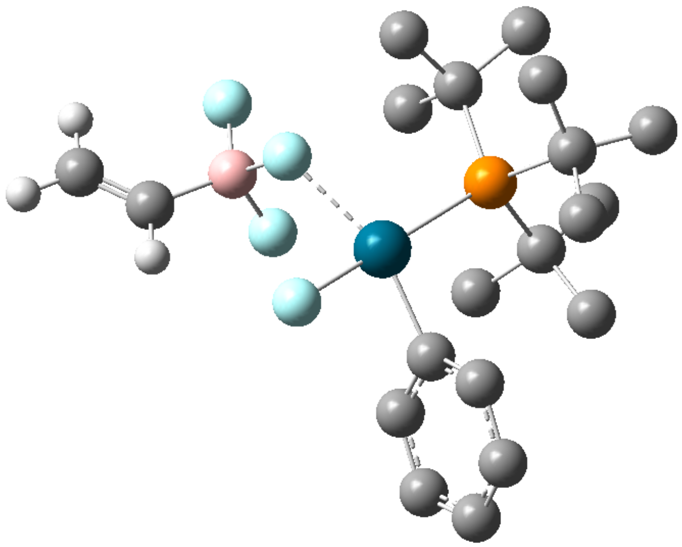

Cartesian coordinates for association anion isomer **S1**

Sum of electronic and thermal free energies: -1675.655906

Pd	-0.41710300	0.80715700	-0.61554400
F	-2.47078700	-0.05299300	-0.92032800
B	-3.35443900	0.15031700	0.25804400
F	-2.48078100	0.50668300	1.31334500
F	-3.92117800	-1.08984300	0.53047400
C	-4.40357600	1.30068100	-0.08190600

C	-5.72735800	1.15402200	-0.01475800
C	0.97933600	2.15761400	-0.17624100
C	2.01990500	2.60134900	-0.99263600
C	2.84742500	3.65114100	-0.58937400
C	2.63248300	4.29575500	0.62343700
C	1.54857300	3.91042000	1.40920600
C	0.72010600	2.86795900	1.00347400
P	0.76438500	-1.19195000	0.00431600
C	1.78866400	-0.96717400	1.63173400
C	2.27063200	-2.24975100	2.31612300
C	0.92017600	-0.19588900	2.63325400
C	3.02296900	-0.09273200	1.37514200
C	2.00686800	-1.72331900	-1.37643700
C	1.23791100	-2.31953600	-2.55939000
C	3.09299300	-2.71904200	-0.96250200
C	2.68165800	-0.46932300	-1.93264500
C	-0.39966300	-2.72177900	0.28719700
C	0.29432000	-4.08509200	0.38137400
C	-1.43382800	-2.83247400	-0.84270700
C	-1.19555800	-2.48667200	1.57678100
H	-3.99747700	2.26958900	-0.39291500
H	-6.43803600	1.95252500	-0.24825800
H	-6.16837300	0.19910600	0.28285500
H	2.19725700	2.14063900	-1.96195000
H	3.66301000	3.96936700	-1.24098000
H	3.28425000	5.11007600	0.94050000
H	1.33135800	4.43763400	2.33924200
H	-0.16137600	2.61678700	1.59587300
H	2.87925000	-2.88914000	1.66546400
H	2.90185200	-1.96350500	3.17291900
H	1.44543200	-2.84847700	2.71856100
H	0.66289100	0.79705100	2.25537100
H	1.49859800	-0.06303800	3.56219600
H	-0.01265600	-0.70300600	2.88932300
H	3.79818700	-0.60251700	0.78971700
H	2.77374400	0.85487100	0.88378300
H	3.47044000	0.15480800	2.35085900
H	0.88716200	-3.34002600	-2.37088200
H	0.37866500	-1.69734900	-2.84554600
H	1.91741100	-2.36624700	-3.42540700
H	3.80127900	-2.29475800	-0.24102300
H	3.67472200	-2.99350900	-1.85787800
H	2.69028800	-3.64630000	-0.54002000
H	1.93388500	0.18275600	-2.40064500
H	3.21648100	0.11973600	-1.18194100
H	3.40693100	-0.77123100	-2.70616200

H	1.07026000	-4.14670900	1.15095100
H	-0.47498200	-4.83186500	0.63558100
H	0.73157100	-4.39969400	-0.57500400
H	-1.99105500	-1.90603700	-0.99001800
H	-2.16199000	-3.60480000	-0.54982900
H	-0.99976600	-3.14845200	-1.79517900
H	-0.58590300	-2.63056400	2.47785800
H	-1.66710100	-1.49649100	1.60525900
H	-2.01167900	-3.22345100	1.61771300
F	-1.33565700	2.44767700	-1.24627900

Potassium (vinyl)trifluoroborate *π*-Association Complexes

(**Structure II**)

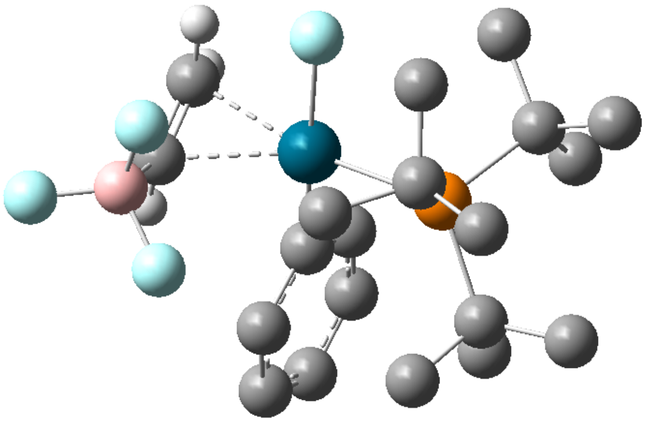

Cartesian coordinates for **Structure II**

Sum of electronic and thermal free energies: -1675.678683

Pd	0.74733400	-0.28870000	-0.80233400
C	2.64418700	-0.82708100	-1.88653200
C	3.04702700	-0.69786000	-0.57895100
F	4.40566300	-2.08378000	0.91901600
F	2.48379600	-1.16945600	1.78393700
B	3.06959400	-1.78510500	0.64334500
F	2.35797800	-2.94215000	0.32753000
C	1.36672000	1.54683700	-0.27741000
C	1.19798800	2.56616100	-1.22233400
C	1.59603100	3.87627700	-0.95058500
C	2.18821100	4.18859600	0.26931300
C	2.40050000	3.17333800	1.20157400
C	2.00184000	1.86597300	0.93152500
P	-1.51169900	-0.08147900	0.11097300
C	-2.75490500	0.22018500	-1.32682400

C	-4.20181300	-0.19212800	-1.04526900
C	-2.25386800	-0.53178500	-2.56513600
C	-2.75422300	1.70281500	-1.71069000
C	-1.89107400	1.24691600	1.47339700
C	-1.10843500	0.86677400	2.73664700
C	-3.37369100	1.41752900	1.82673800
C	-1.37661800	2.64343300	1.09251500
C	-1.89372200	-1.80901700	0.90836500
C	-3.09040000	-1.85668400	1.86251400
C	-0.62833300	-2.24748800	1.66075000
C	-2.13204700	-2.87295600	-0.16996500
H	2.84848900	-0.04205900	-2.62309900
H	2.25777400	-1.77223600	-2.26883300
H	0.73923000	2.33671400	-2.18850200
H	1.44210200	4.65547600	-1.69926000
H	2.49851200	5.21103400	0.48616000
H	2.88873400	3.40003300	2.15083200
H	2.20523900	1.06618100	1.64637100
H	-4.63207400	0.31921200	-0.17627500
H	-4.81883200	0.07413600	-1.91942000
H	-4.30937200	-1.27173500	-0.89382100
H	-1.27150900	-0.15840100	-2.88250700
H	-2.97561300	-0.36414700	-3.38225300
H	-2.13014600	-1.60546800	-2.41358000
H	-3.28584700	2.33341400	-0.98761700
H	-1.73583800	2.09539600	-1.83907700
H	-3.27498400	1.80967000	-2.67575000
H	-0.04642800	0.69057600	2.51797500
H	-1.16443100	1.70835700	3.44545000
H	-1.50584100	-0.01568900	3.24899800
H	-3.92603600	1.92605900	1.02533100
H	-3.44326600	2.06419000	2.71676000
H	-3.89404000	0.48506300	2.06097200
H	-0.28770700	2.67641500	1.03593400
H	-1.77698700	3.03235800	0.15371700
H	-1.68835900	3.33682200	1.89075200
H	-4.03319000	-1.56453000	1.37998300
H	-3.20948800	-2.90086500	2.19264800
H	-2.96080300	-1.25360000	2.76878100
H	0.22897100	-2.39913300	0.99250300
H	-0.84249500	-3.21420500	2.14538100
H	-0.31135600	-1.55293300	2.44429800
H	-3.09236900	-2.75436500	-0.68868600
H	-1.30509700	-2.87516000	-0.89075000
H	-2.16272300	-3.85063100	0.33726400
H	3.53136200	0.25731700	-0.34678200

F	0.14082200	-2.04176500	-1.71000400

(($(tBu)_3P$)Pd(Ph)(F)(BF_3(CH=CH_2)) rearrangement π-complex **S3**

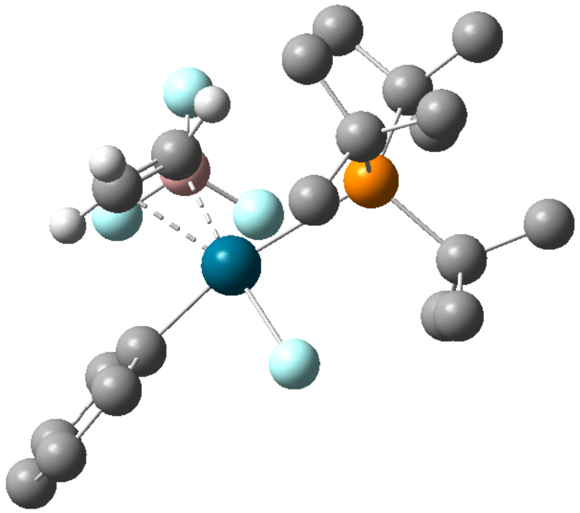

Cartesian coordinates for π-complex **S3**
Sum of electronic and thermal free energies: -1675.670679

Pd	-0.92412700	-0.26311300	0.17628500
C	-1.72995800	1.03644200	1.81468700
C	-0.79417000	1.75943900	1.10756800
F	-0.42638600	4.03339300	0.24483900
F	-0.56553800	2.36276600	-1.31685700
B	-1.08621700	2.83909800	-0.08730500
F	-2.45992000	3.05443600	-0.22371000
C	-2.90070500	-0.62451500	0.07072300
C	-3.39684900	-1.80588500	0.61832300
C	-4.73277900	-2.15334000	0.42709000
C	-5.57238200	-1.32982900	-0.31950900
C	-5.06980900	-0.15103700	-0.86275400
C	-3.73764300	0.21399100	-0.66143800
P	1.73757900	-0.27535300	-0.02147400
C	2.29390100	-1.22009800	1.56448900
C	3.66276100	-1.90199600	1.52439300
C	1.22307300	-2.27801600	1.86719500
C	2.28396900	-0.25670200	2.75518400
C	2.85761900	1.31494100	-0.16867500
C	2.66315500	1.92164300	-1.56470200
C	4.34941000	1.07963200	0.09511100
C	2.42185600	2.43897200	0.78586200

C	2.20436000	-1.37239900	-1.55293500
C	3.66647700	-1.34981700	-2.00567600
C	1.29531800	-0.89350900	-2.69665000
C	1.84477000	-2.84648000	-1.32179200
H	-1.48313200	0.50343800	2.73974100
H	-2.79376600	1.18280300	1.63889000
H	-2.73018000	-2.46789400	1.17271000
H	-5.11602700	-3.08066200	0.85657000
H	-6.61622700	-1.60525400	-0.47394500
H	-5.72104200	0.50397200	-1.44353200
H	-3.36016700	1.16279300	-1.04372500
H	4.48440000	-1.19561400	1.35972000
H	3.84483600	-2.39496200	2.49422300
H	3.72427300	-2.67823100	0.75364100
H	0.22738400	-1.82232000	1.97771700
H	1.48240100	-2.78191500	2.81338300
H	1.13450100	-3.04074100	1.08912200
H	3.11210900	0.46212200	2.72930000
H	1.33937500	0.30117400	2.82876400
H	2.39218100	-0.84237900	3.68253900
H	1.59958200	2.11369600	-1.75973800
H	3.18001400	2.89462400	-1.58606600
H	3.08119200	1.31867100	-2.37746300
H	4.55060100	0.91333900	1.16225300
H	4.90002700	1.99131200	-0.18982900
H	4.78412100	0.24799100	-0.46623200
H	1.47799700	2.90582900	0.48736000
H	2.35252900	2.13716000	1.83583400
H	3.19038100	3.22764500	0.73316800
H	4.35264400	-1.72029900	-1.23128500
H	3.76161400	-2.02862300	-2.86885600
H	4.01820300	-0.36683400	-2.33649500
H	0.25092600	-1.11729100	-2.44854000
H	1.56314700	-1.44596900	-3.61282900
H	1.38136400	0.17567600	-2.91399300
H	2.51495100	-3.34883700	-0.61248700
H	0.79918900	-2.94343000	-1.01112600
H	1.95963800	-3.36696800	-2.28714000
H	0.20309400	1.74522800	1.55026800
F	-0.87694700	-2.00502400	-0.86553000

((tBu)$_3$P)Pd(Ph)(F)(BF$_3$(CH=CH$_2$)) rearrangement isomer **S4**

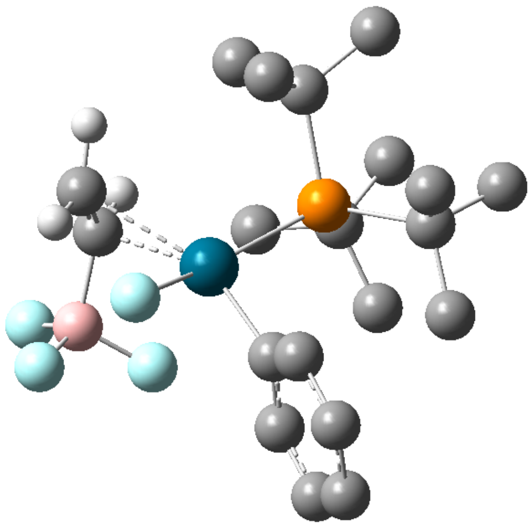

Cartesian coordinates for rearrangement isomer **S4**

Sum of electronic and thermal free energies: -1675.649115

Pd	-0.88050600	0.09648900	0.73314900
C	-1.50381000	2.27439600	1.75889800
C	-1.49278700	2.51368300	0.42418400
F	-2.97022200	3.43188800	-1.29901900
F	-2.13642500	1.33342300	-1.69299100
B	-2.66152500	2.21263700	-0.68594000
F	-3.77899800	1.63367800	-0.11315200
C	-1.25166800	-1.77163200	0.15302300
C	-1.22545500	-2.86139200	1.02145500
C	-1.63268000	-4.11782900	0.57695400
C	-2.09783400	-4.28573400	-0.72616500
C	-2.18370300	-3.18105900	-1.56980600
C	-1.76916000	-1.92241400	-1.13317400
P	1.39518600	0.12904600	-0.08986500
C	2.33280900	1.09729100	1.31771500
C	3.85535100	0.94755100	1.38680200
C	1.75480900	0.66006000	2.67289600
C	2.04669400	2.59695300	1.18319400
C	1.77989600	1.02788300	-1.76486500
C	1.39178100	0.09121500	-2.91360100
C	3.22443000	1.49018800	-1.97585400
C	0.87376900	2.25270800	-1.91956000
C	2.30030000	-1.59642500	-0.20926400

C	3.72615800	-1.54960900	-0.77436000
C	1.53125400	-2.61295900	-1.07255000
C	2.35655100	-2.22162800	1.18905000
H	-0.72751600	2.65710200	2.43195200
H	-2.33918900	1.75672900	2.22927800
H	-0.90684900	-2.72090200	2.05491500
H	-1.59805300	-4.96953000	1.25857500
H	-2.41704400	-5.26896100	-1.07307000
H	-2.58599600	-3.29387900	-2.57771900
H	-1.87457800	-1.04844300	-1.77829300
H	4.35882300	1.24551800	0.45883300
H	4.22761700	1.61201100	2.18360900
H	4.17889100	-0.06654500	1.64735700
H	0.66143300	0.75725900	2.70843600
H	2.18421300	1.30211500	3.45932300
H	1.99871800	-0.37590200	2.92751300
H	2.57466600	3.05517000	0.33830100
H	0.97925600	2.81314500	1.08461400
H	2.40401000	3.10021400	2.09563700
H	0.37643100	-0.30955200	-2.78516200
H	1.39505200	0.67200400	-3.84884300
H	2.09295000	-0.74079900	-3.04864400
H	3.51616600	2.28033500	-1.27296100
H	3.29949600	1.92443200	-2.98604400
H	3.96512900	0.68689400	-1.91418900
H	-0.18942500	1.98788300	-1.87535900
H	1.07781200	3.03488100	-1.18195800
H	1.06926100	2.69147000	-2.91140500
H	4.37786400	-0.80867500	-0.30330500
H	4.18791200	-2.53748500	-0.61460000
H	3.73068400	-1.37393100	-1.85749200
H	0.65320200	-3.00233600	-0.55580200
H	2.20814900	-3.46207100	-1.26223100
H	1.20146600	-2.23430200	-2.04179800
H	3.09469600	-1.74847200	1.84704200
H	1.37567200	-2.20330300	1.68117900
H	2.64977500	-3.27824600	1.08305200
H	-0.64507200	3.10312800	0.05491700
F	-2.42243900	-0.36141100	1.95486700

Transmetalation structures **III-V**

Structure **III**

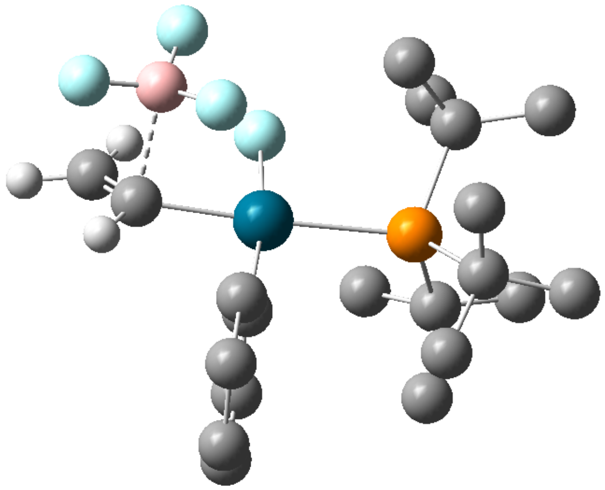

Cartesian coordinates for Structure III

Sum of electronic and thermal free energies: -1675.651047

Pd	0.79572400	-0.32407800	-0.53135300
C	3.05715600	-1.55743900	-1.93477300
C	2.80180800	-0.63653600	-0.98687800
F	4.41931300	-1.73419100	0.75730400
F	2.54144900	-1.01549100	1.80500000
B	3.07079300	-1.77010500	0.81163800
F	2.49099000	-2.95900800	0.61284900
C	1.41774000	1.55721800	-0.20171500
C	1.23795300	2.51852600	-1.21033800
C	1.63523100	3.84449800	-1.04308500
C	2.25006900	4.24966900	0.13913100
C	2.48498800	3.30202500	1.13292100
C	2.08439900	1.97763600	0.96039300
P	-1.58066000	-0.07512000	0.07083100
C	-2.62937600	0.30883500	-1.49414600
C	-4.12173400	-0.01960500	-1.42070300
C	-1.99053700	-0.46360800	-2.65549000
C	-2.49997500	1.79259100	-1.85053700
C	-2.05305100	1.23890600	1.41376200
C	-1.42593100	0.79284200	2.74009600
C	-3.54985200	1.49650800	1.61404300
C	-1.40924100	2.60014600	1.11318100
C	-2.16357800	-1.79259600	0.76489600
C	-3.45483200	-1.79880300	1.58536400
C	-1.00284700	-2.33356000	1.61587600
C	-2.33974400	-2.81575900	-0.36551200
H	4.00410300	-1.60010100	-2.48707900

H	2.31063000	-2.32515000	-2.14996000
H	0.77109100	2.22285700	-2.15422100
H	1.46546900	4.56610500	-1.84503900
H	2.55794100	5.28680900	0.27695300
H	2.98803000	3.59625800	2.05646800
H	2.29134300	1.24817100	1.74360000
H	-4.63396500	0.52241800	-0.61595300
H	-4.59821600	0.27869200	-2.36989000
H	-4.31735500	-1.08908100	-1.28649700
H	-0.93095600	-0.19515100	-2.77243300
H	-2.52704800	-0.20969000	-3.58533600
H	-2.02101900	-1.54818900	-2.52542000
H	-3.09423900	2.43952900	-1.19280200
H	-1.45694800	2.13649200	-1.83074700
H	-2.88076100	1.93910300	-2.87443300
H	-0.35327700	0.58289900	2.62113400
H	-1.52439900	1.61355300	3.46879200
H	-1.90457100	-0.08955200	3.17885300
H	-3.98859800	2.01979700	0.75372900
H	-3.67833700	2.15985300	2.48568700
H	-4.14068900	0.59498500	1.80002100
H	-0.31788000	2.54271300	1.09758400
H	-1.73450100	3.05001400	0.17165200
H	-1.69895900	3.29194000	1.92147600
H	-4.32023600	-1.43196100	1.01589900
H	-3.67317600	-2.84228900	1.86547400
H	-3.38907900	-1.22922200	2.51996500
H	-0.13530000	-2.53308800	0.97381800
H	-1.32209100	-3.28485200	2.07395800
H	-0.69105400	-1.66479900	2.42540800
H	-3.22327300	-2.63033700	-0.98898500
H	-1.42672200	-2.85753900	-0.97290000
H	-2.48655700	-3.80209300	0.10540300
H	3.56404700	0.13789500	-0.82656000
F	0.35906100	-2.24475900	-1.20347800

Structure **IV**

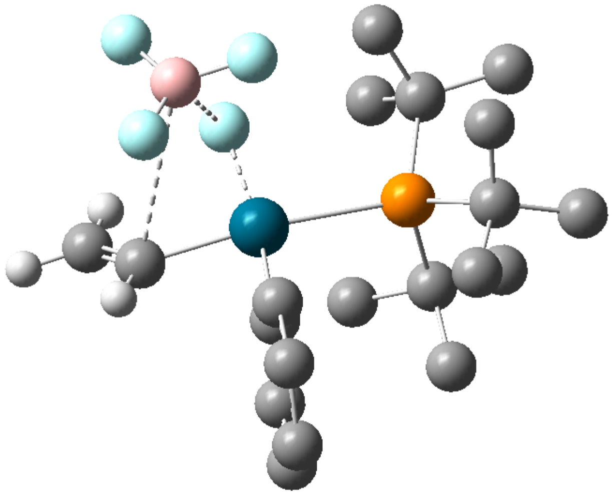

Cartesian coordinates for Structure **IV**

Sum of electronic and thermal free energies: -1675.655691

Pd	0.91598300	-0.36937300	-0.53224600
C	2.80719600	-1.56305000	-2.44547800
C	2.69313600	-0.67917800	-1.44960200
F	3.26545300	-1.81167400	1.22767000
F	1.17452600	-2.30619700	1.95667100
B	2.18256800	-2.56607000	1.13752500
F	2.26698300	-3.73609200	0.54784100
C	1.74982900	1.41090100	-0.09459500
C	1.81746400	2.49478800	-0.98475200
C	2.39385000	3.71360000	-0.62639700
C	2.96002300	3.88106300	0.63430400
C	2.96017300	2.80300000	1.51741500
C	2.37454900	1.59201700	1.15267400
P	-1.52580100	0.11245200	0.05552900
C	-2.18692100	1.09411400	-1.47302400
C	-3.70018300	1.08145000	-1.69918600
C	-1.48863400	0.51868200	-2.71463300
C	-1.75097900	2.55996400	-1.38810600
C	-1.96469200	1.15636100	1.62294300
C	-1.76686500	0.26956700	2.85692500
C	-3.36366800	1.77401300	1.67987700
C	-0.94048200	2.28674300	1.77947000
C	-2.58386500	-1.50809000	0.16375600
C	-4.02351300	-1.36412200	0.66096200
C	-1.83627500	-2.49557900	1.07107100

C	-2.60588800	-2.19289600	-1.20958400
H	3.75009900	-1.71202400	-2.98580600
H	1.97061300	-2.19988300	-2.73641100
H	1.41287300	2.38252200	-1.99442600
H	2.40985800	4.53631600	-1.34471900
H	3.41211600	4.83212800	0.91839500
H	3.42270900	2.90529300	2.50147000
H	2.38818900	0.76155300	1.86413500
H	-4.25750800	1.49628500	-0.84933800
H	-3.93175600	1.70746500	-2.57750100
H	-4.09312300	0.08005100	-1.90747900
H	-0.39468900	0.58782100	-2.62466100
H	-1.80613600	1.09622500	-3.59922500
H	-1.72302500	-0.53381000	-2.89782000
H	-2.31690900	3.13138800	-0.64153600
H	-0.68041300	2.66547200	-1.17327800
H	-1.94142300	3.03426200	-2.36503700
H	-0.79145200	-0.23810200	2.83451200
H	-1.79046400	0.90709000	3.75570900
H	-2.55079100	-0.48739800	2.97565600
H	-3.50479600	2.55042300	0.91717600
H	-3.49528300	2.26385100	2.65961400
H	-4.16998700	1.04073900	1.57089100
H	0.07758400	1.89645700	1.87808300
H	-0.93070900	3.00254500	0.95274300
H	-1.18199900	2.84710400	2.69822800
H	-4.62313600	-0.65910000	0.07121400
H	-4.51276100	-2.34956600	0.58581300
H	-4.08184800	-1.06555000	1.71575800
H	-0.85833700	-2.71318000	0.62639500
H	-2.42297400	-3.42887200	1.11651500
H	-1.69986100	-2.14613100	2.09858600
H	-3.20903300	-1.66479100	-1.95781100
H	-1.57768400	-2.34046300	-1.56680000
H	-3.06148600	-3.18860000	-1.07931600
H	3.57152100	-0.08557500	-1.16634900
F	0.37808800	-2.34351400	-0.91383500

Structure **V**

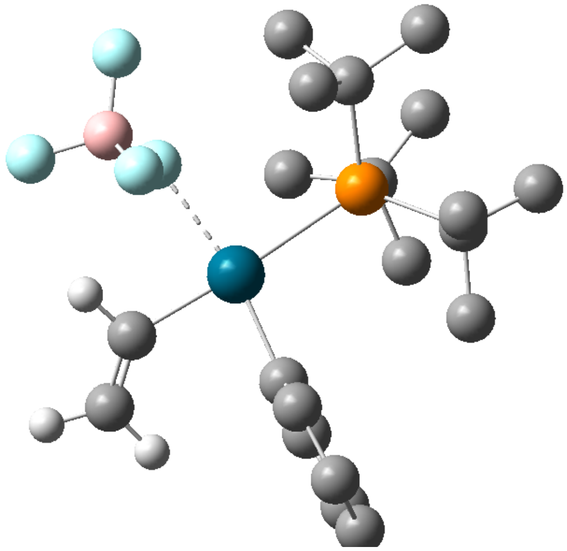

Cartesian coordinates for Structure **V**

Sum of electronic and thermal free energies: 1675.710866

Pd	0.49256600	1.01039000	0.20786300
C	2.42440400	3.20089300	1.23702900
C	1.30978800	2.80899700	0.61761000
F	-1.27604400	2.79932500	-1.56386000
F	-3.33454500	2.75743500	-0.57379200
B	-1.99240900	3.02489900	-0.39126500
F	-1.76441800	4.28631700	0.10458900
C	2.38362800	0.46829600	-0.11883200
C	3.21969800	-0.01845700	0.89577400
C	4.50742200	-0.47546900	0.62081000
C	5.00692400	-0.43374700	-0.67862200
C	4.20671100	0.08787400	-1.69271700
C	2.91656700	0.53747200	-1.41464600
P	-0.71063200	-1.25100200	0.02744200
C	-0.93654600	-1.80880600	1.86002800
C	-1.99978500	-2.87341200	2.13557200
C	-1.25912100	-0.55140500	2.67962800
C	0.39717300	-2.32940000	2.40375100
C	0.15791400	-2.71285700	-0.90336500
C	0.06949700	-2.44444000	-2.40982400
C	-0.37954300	-4.11669200	-0.61199000
C	1.66146400	-2.72769700	-0.59866000
C	-2.48069900	-1.09622100	-0.74872600
C	-3.18832900	-2.40123000	-1.12182900
C	-2.34682400	-0.22842700	-2.00845300
C	-3.41705200	-0.33404500	0.19709500
H	2.60064000	4.25531200	1.47703300
H	3.21785000	2.51115900	1.53327800
H	2.85702300	-0.03700300	1.92683800

H	5.12744700	-0.86415000	1.43105600
H	6.01430700	-0.78983900	-0.89597900
H	4.59158800	0.15040600	-2.71231000
H	2.30707200	0.95022700	-2.22056200
H	-1.81250400	-3.81029400	1.59678300
H	-1.99425400	-3.10971300	3.21283700
H	-3.01248700	-2.53625900	1.88810400
H	-0.47012900	0.20816000	2.56994000
H	-1.32289000	-0.83106400	3.74437100
H	-2.20452400	-0.07836500	2.40105500
H	0.65790800	-3.32210000	2.01612100
H	1.22522300	-1.63974800	2.19168200
H	0.31418700	-2.42273400	3.49868600
H	0.40427300	-1.42639400	-2.65550600
H	0.74072000	-3.14632500	-2.93015600
H	-0.93444000	-2.59103400	-2.82312200
H	-0.14230600	-4.43985100	0.41007400
H	0.11451200	-4.83073600	-1.29195500
H	-1.45938000	-4.21884200	-0.75902900
H	2.15328200	-1.81566500	-0.94876900
H	1.90438600	-2.84832300	0.45993000
H	2.10721700	-3.58036600	-1.13716300
H	-3.32090900	-3.07499300	-0.26475000
H	-4.19437700	-2.14973100	-1.49520200
H	-2.68501000	-2.95653500	-1.92232800
H	-1.90919300	0.75437600	-1.79767600
H	-3.35501500	-0.05087300	-2.41597600
H	-1.75542100	-0.70366500	-2.79806100
H	-3.72220900	-0.93034500	1.06574700
H	-2.99328800	0.61573100	0.53008100
H	-4.33253800	-0.08649100	-0.36222100
H	0.56948100	3.57125700	0.34007200
F	-1.49638400	2.06026100	0.59776200

Potassium (phenyl)trifluoroborate Association Complexes

Structure **VI**

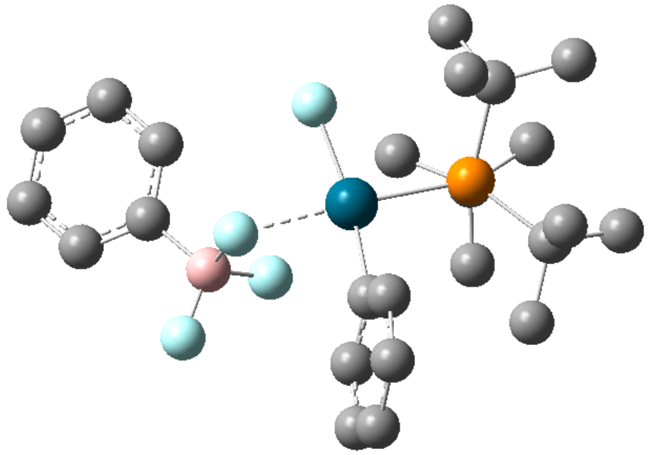

Cartesian coordinates for Structure **VI**

Sum of electronic and thermal free energies: -1829.165213

Pd	-0.06870800	0.18198300	-0.62196500
F	-2.11504500	0.96627200	-0.87878400
B	-2.92726100	0.91360800	0.35654200
F	-3.31601000	2.20667100	0.65595200
F	-2.04454000	0.43895600	1.36081200
C	0.45468700	2.08035500	-0.35919800
C	1.12761400	2.75457700	-1.38343100
C	1.36121300	4.12873100	-1.30228800
C	0.91395200	4.84810000	-0.19843600
C	0.20504100	4.18931500	0.80507300
C	-0.02921900	2.81914100	0.72517300
P	1.90179300	-0.77239000	0.12431800
C	3.44965000	0.30950800	0.57383400
C	4.51904700	-0.43288600	1.38558200
C	3.06707000	1.56022400	1.38139500
C	4.09512400	0.84923600	-0.70768500
C	2.48431700	-2.03748900	-1.21905800
C	1.52772400	-3.23445100	-1.29400700
C	3.89952900	-2.59805800	-1.05672300
C	2.36650400	-1.31885700	-2.57108800
C	1.39647100	-1.73666300	1.71348400
C	2.34104800	-2.87781700	2.10116900
C	-0.01789700	-2.30488400	1.52840000
C	1.29495600	-0.76557000	2.89369300
H	1.47150300	2.20331800	-2.26269800
H	1.89267200	4.63663000	-2.10890600

H	1.09889300	5.92032200	-0.12892200
H	-0.18188400	4.75076400	1.65648100
H	-0.61906600	2.31756400	1.49199200
H	4.83653200	-1.38408800	0.94783100
H	5.41069900	0.21149300	1.44809400
H	4.19288800	-0.62025700	2.41616500
H	2.56055800	2.30366200	0.76216000
H	4.00039700	2.01330900	1.75357200
H	2.43040700	1.36242500	2.24656000
H	4.58162500	0.08078100	-1.31768000
H	3.36750300	1.38902600	-1.32620000
H	4.87285200	1.57386400	-0.41808100
H	1.62803700	-3.91265700	-0.43748000
H	0.48693700	-2.89521800	-1.38573800
H	1.79569900	-3.81487900	-2.19203900
H	4.69244600	-1.84565900	-1.13634800
H	4.06809000	-3.32603500	-1.86626000
H	4.02833500	-3.13838300	-0.10934700
H	1.32487600	-1.02597100	-2.75846400
H	2.99546200	-0.42489200	-2.65100600
H	2.67609300	-2.01498700	-3.36734200
H	3.37432000	-2.55488900	2.27046400
H	1.97957900	-3.31731600	3.04448400
H	2.35081800	-3.68403400	1.35890900
H	-0.76433200	-1.50518000	1.44891500
H	-0.25199800	-2.90668000	2.42184000
H	-0.13409000	-2.93281000	0.64411400
H	2.26881300	-0.41258800	3.25550400
H	0.65928200	0.09752900	2.65330600
H	0.81285000	-1.29743400	3.72844400
F	-0.86032800	-1.60772300	-1.25749700
C	-4.15671600	-0.09219000	0.12622200
C	-5.46238900	0.25219600	0.48578300
C	-3.94482600	-1.36953200	-0.41239100
C	-6.52489200	-0.63502700	0.32381300
H	-5.64535700	1.24554600	0.89953700
C	-5.00039200	-2.26242000	-0.57829200
H	-2.93155100	-1.64597400	-0.72050100
C	-6.29497100	-1.90008200	-0.20922300
H	-7.53601600	-0.33930400	0.61137700
H	-4.81443200	-3.25037800	-1.00402900
H	-7.12201000	-2.60035400	-0.34093500

((tBu)$_3$P)Pd(Ph)(F)(BF$_3$(Ph)) association anion **S5**

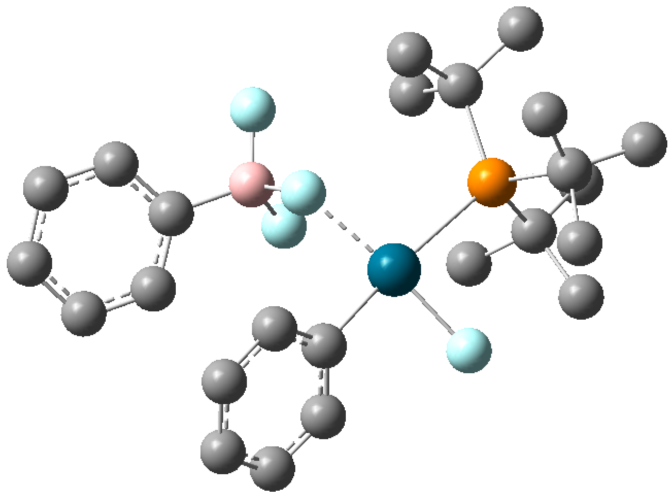

Cartesian coordinates for association anion isomer **S5**

Sum of electronic and thermal free energies: -1829.160612

Pd	-0.05916500	-0.86761200	-0.19349600
F	1.06400700	0.89407000	-0.73781100
B	1.68408100	1.75207700	0.33890200
F	1.18978100	1.23094400	1.53441600
F	1.18955500	3.02725800	0.10754300
C	1.67708300	-1.85182600	-0.24829900
C	2.59914300	-1.60651200	-1.26490000
C	3.82346200	-2.27505100	-1.27612300
C	4.14123900	-3.17537500	-0.26369100
C	3.22119500	-3.41707600	0.75427900
C	1.98653700	-2.76994400	0.75512600
P	-2.36486900	0.18148700	-0.05523600
C	-3.07160300	-0.32589500	1.66492000
C	-4.29345200	0.45178500	2.15524100
C	-1.92518500	-0.16502600	2.67504900
C	-3.42247300	-1.81881700	1.67355600
C	-3.51507600	-0.55529400	-1.41099200
C	-3.21873000	0.12167900	-2.75252300
C	-5.01762500	-0.44736500	-1.14609900
C	-3.14123300	-2.02959300	-1.61239700
C	-2.46239700	2.10037000	-0.18952400
C	-3.85444200	2.70924400	-0.36631000
C	-1.58761600	2.54525100	-1.37133900
C	-1.82228300	2.70531400	1.06597500
H	2.37970100	-0.86974800	-2.03781200
H	4.54293500	-2.06721100	-2.06934300
H	5.10534300	-3.68514700	-0.26546200
H	3.46186000	-4.12274200	1.55142600
H	1.25215500	-2.98289200	1.53106100

H	-5.14786800	0.37172000	1.46990700
H	-4.61169100	0.02919100	3.12267300
H	-4.08860700	1.51566200	2.32436800
H	-1.05589900	-0.77186200	2.38602900
H	-2.27535700	-0.51398800	3.66056300
H	-1.57916000	0.86603000	2.79009000
H	-4.32462500	-2.05050600	1.09295500
H	-2.58001800	-2.41791300	1.30446800
H	-3.63243700	-2.11008400	2.71588200
H	-3.60800000	1.14470700	-2.81528600
H	-2.14190100	0.13971600	-2.97105400
H	-3.70825600	-0.45981400	-3.55023300
H	-5.33307000	-1.03037600	-0.27273300
H	-5.56353000	-0.85005900	-2.01578000
H	-5.35137600	0.58775400	-1.00178400
H	-2.09383200	-2.13237300	-1.92199300
H	-3.26175400	-2.64502400	-0.71905100
H	-3.78517400	-2.44190000	-2.40747000
H	-4.54949500	2.44606600	0.44060900
H	-3.75627100	3.80745700	-0.37002000
H	-4.31788000	2.42908000	-1.32123600
H	-0.58703600	2.10572400	-1.32589700
H	-1.46226300	3.63805200	-1.31730200
H	-2.03504600	2.31815700	-2.34359300
H	-2.45771300	2.60415500	1.95424200
H	-0.83337900	2.28287400	1.27911200
H	-1.67341600	3.78244800	0.89373400
F	-0.87086200	-2.62058000	0.30399200
C	3.28101700	1.67050600	0.22269400
C	4.00978300	2.67129800	-0.42844600
C	4.00345700	0.60102900	0.76477500
C	5.39632600	2.61054300	-0.54580500
H	3.46970500	3.52265000	-0.84558800
C	5.38883600	0.52577100	0.65275900
H	3.46307500	-0.19707700	1.27748200
C	6.09169100	1.53238700	-0.00478100
H	5.93814700	3.40704700	-1.05908000
H	5.92048400	-0.32882100	1.07390000
H	7.17764900	1.47683800	-0.09505600

((tBu)$_3$P)Pd(Ph)(F)(BF$_3$(Ph)) association anion isomer **S6**

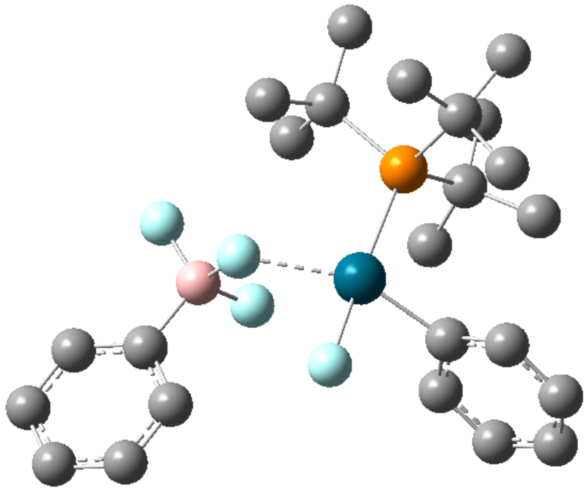

Cartesian coordinates for association anion isomer **S6**

Sum of electronic and thermal free energies: -1829.155054

Pd	-0.08145400	0.57931500	-0.61811800
F	-1.78858200	-0.91449100	-0.90416600
B	-2.58328000	-1.13663400	0.31937200
F	-1.88436200	-0.45139400	1.34141800
F	-2.54666400	-2.50567700	0.57820000
C	0.72932100	2.32203100	-0.10301200
C	1.54605100	3.15018900	-0.87162800
C	1.91712200	4.41299200	-0.40511900
C	1.45529900	4.87906000	0.82050000
C	0.57823600	4.08696500	1.55776400
C	0.20322600	2.82998800	1.09110500
P	1.73447300	-0.86970000	-0.01405400
C	2.65430200	-0.32964500	1.60004700
C	3.57937600	-1.37553200	2.22889100
C	1.59520600	0.05816900	2.64007500
C	3.49159700	0.93178400	1.35441900
C	3.04124100	-0.89233500	-1.43650600
C	2.49536400	-1.69346000	-2.62274600
C	4.42225300	-1.44763800	-1.07977800
C	3.21421800	0.53273900	-1.96311200
C	1.19483100	-2.71605000	0.24515500
C	2.32561500	-3.75010200	0.27729900
C	0.22932700	-3.14904200	-0.86738300
C	0.41508900	-2.80821400	1.56257000
H	1.89949100	2.82860300	-1.84876100
H	2.56838800	5.03829700	-1.01803000
H	1.75181200	5.86262100	1.18503800

H	0.16420500	4.45563700	2.49708400
H	-0.53007300	2.24326600	1.64716300
H	4.35388600	-1.73903500	1.54282600
H	4.09490700	-0.90717100	3.08279800
H	3.03265800	-2.23876500	2.62461300
H	1.01251300	0.92210100	2.30882400
H	2.11253000	0.34002500	3.57173200
H	0.89187600	-0.74362100	2.87650400
H	4.38388600	0.74416900	0.74445400
H	2.91190700	1.74085800	0.89566700
H	3.84442100	1.29786000	2.33165700
H	2.53410200	-2.77651700	-2.46240600
H	1.46333400	-1.40910600	-2.87024700
H	3.11974700	-1.47550400	-3.50382100
H	4.95828400	-0.81530600	-0.36202500
H	5.03319700	-1.47696200	-1.99692700
H	4.39025000	-2.46712600	-0.67955800
H	2.26855000	0.89616400	-2.38457700
H	3.54220900	1.24912800	-1.20407400
H	3.96856600	0.52305700	-2.76688800
H	3.09592600	-3.55475500	1.03073900
H	1.87512000	-4.72631500	0.51843900
H	2.81719300	-3.86489900	-0.69713500
H	-0.60460900	-2.45696300	-0.99470500
H	-0.20329900	-4.11817900	-0.57548700
H	0.72565400	-3.29663100	-1.83045400
H	1.06981600	-2.74941000	2.44110900
H	-0.37372100	-2.05064900	1.63889100
H	-0.08789400	-3.78580000	1.59785300
F	-1.46339700	1.82199600	-1.33524800
C	-4.06910000	-0.57245800	0.11463100
C	-4.28242700	0.70447500	-0.42306300
C	-5.19119300	-1.31344900	0.49598400
C	-5.56948500	1.21664000	-0.56435600
H	-3.41366900	1.28686100	-0.74403400
C	-6.48285600	-0.80848100	0.35867300
H	-5.04309200	-2.31319900	0.90846700
C	-6.67515200	0.46360600	-0.17246700
H	-5.71348500	2.21246100	-0.98736800
H	-7.34243300	-1.40852400	0.66452400
H	-7.68368500	0.86623800	-0.28399600

Potassium (phenyl)trifluoroborate π-Association Complexes

Structure **VIII**

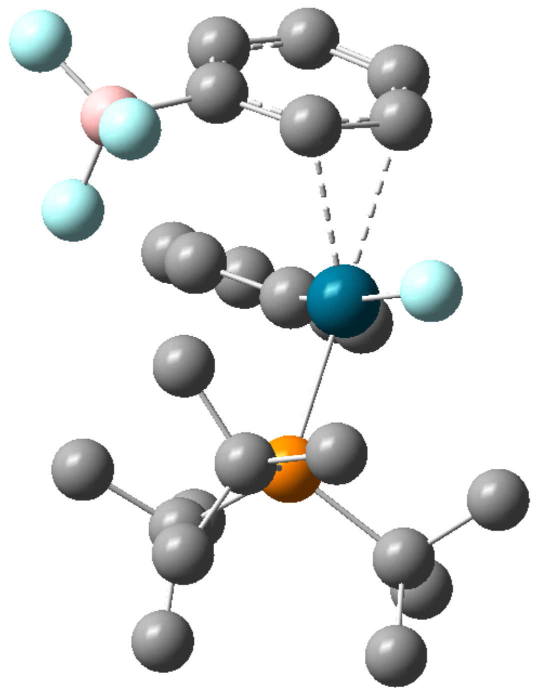

Cartesian coordinates for Structure **VIII**

Sum of electronic and thermal free energies: -1829.165066

*note that the C atoms are not those involved in C-C bond formation (not interacting with borate)

Pd	-0.21581200	0.04850800	1.00569400
F	-4.03004000	-2.45200100	-1.72893800
F	-1.91340600	-1.59495900	-1.96037000
B	-2.84761700	-2.18256300	-1.04112300
F	-2.27458400	-3.35380500	-0.53300500
C	-0.89928500	1.57346300	-0.08105900
C	-0.68528100	2.83800200	0.48003400
C	-1.24246300	3.97989200	-0.09463300
C	-2.01448100	3.87310500	-1.24878700
C	-2.22799000	2.61801900	-1.81291600
C	-1.67539200	1.47275300	-1.23883900
P	1.86613500	-0.08458400	-0.12478300
C	3.22585200	0.73633700	0.95911900
C	4.64747500	0.21560500	0.73379800
C	2.84214500	0.53514000	2.42946000
C	3.23696400	2.25042600	0.73035800
C	2.06169900	0.59806900	-1.92629500
C	1.17503000	-0.23993900	-2.85697300
C	3.50535600	0.58077300	-2.44706500
C	1.57379100	2.04729500	-2.08026100

C	2.19595500	-1.99495400	-0.21026900
C	3.30033900	-2.43833600	-1.17376800
C	0.87465000	-2.66929000	-0.60978200
C	2.55384700	-2.54984600	1.17423400
H	-0.09093900	2.93533300	1.39345900
H	-1.07240700	4.95562500	0.36370000
H	-2.44852900	4.76375400	-1.70409100
H	-2.83496100	2.52103400	-2.71444500
H	-1.86058300	0.49558000	-1.68669900
H	4.98905500	0.33897900	-0.30062000
H	5.33610100	0.78946900	1.37547100
H	4.75887900	-0.83935100	1.00679300
H	1.89424800	1.03986600	2.66040700
H	3.63468600	0.97505600	3.05744700
H	2.70347700	-0.50978400	2.71410600
H	3.68902300	2.53537700	-0.22748800
H	2.22953100	2.68555900	0.78715700
H	3.84389500	2.71365500	1.52465300
H	0.14665900	-0.34594500	-2.48675600
H	1.12497200	0.27001000	-3.83198300
H	1.57039900	-1.24499300	-3.04033400
H	4.11934800	1.35750900	-1.97164600
H	3.48151000	0.81194700	-3.52413200
H	4.01752900	-0.37797000	-2.33308600
H	0.49127100	2.13245300	-1.97463000
H	2.03893300	2.75597900	-1.39108300
H	1.83267200	2.36896800	-3.10206000
H	4.27764200	-1.99581900	-0.93733900
H	3.40914300	-3.52933100	-1.07050400
H	3.07167700	-2.24404400	-2.22761400
H	0.11391900	-2.56679700	0.17173700
H	1.06476800	-3.74683400	-0.73784500
H	0.41913900	-2.30131000	-1.53255700
H	3.56077200	-2.26577800	1.50713400
H	1.80603500	-2.25188100	1.92052400
H	2.54393800	-3.64868500	1.09450400
F	0.34445200	-1.23995000	2.53608700
C	-3.10461500	-1.10843500	0.15532300
C	-2.32002300	-1.12787300	1.32726900
C	-4.00787200	-0.05795200	0.03153400
C	-2.37896000	-0.09446000	2.28422600
H	-1.71250000	-2.00789900	1.53755200
C	-4.10611800	0.96426600	0.98603500
H	-4.64518900	-0.02489100	-0.85507400
C	-3.28350200	0.96496400	2.09883700
H	-1.79778600	-0.18158900	3.20242700

H	-4.81546100	1.78009400	0.83606000
H	-3.33797100	1.77114400	2.83084300

((tBu)$_3$P)Pd(Ph)(F)(BF_3(Ph)) rearrangement π-complex **S7**

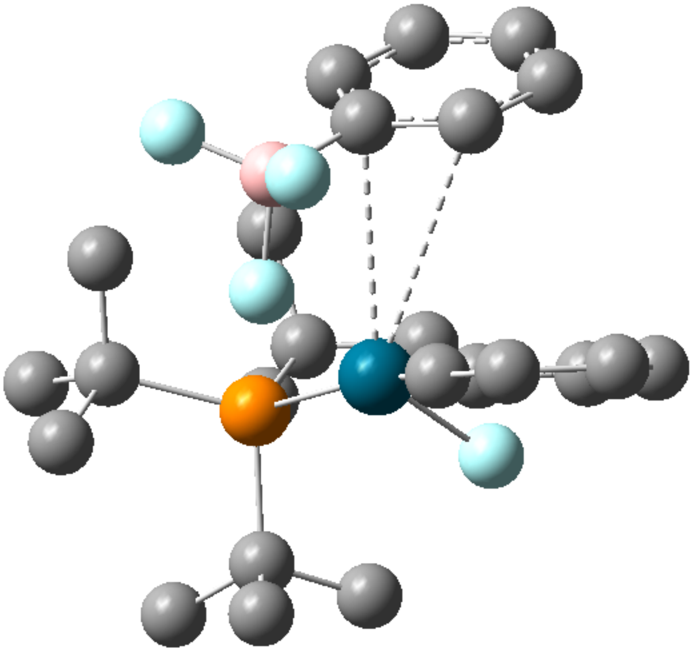

Cartesian coordinates for π-complex **S7**

Sum of electronic and thermal free energies: -1829.157902

Pd	-0.42986000	-0.74199700	-0.01346400
F	-1.23512000	2.62550000	-2.44978700
F	-0.93968600	0.46171400	-1.72740100
B	-1.88666600	1.66589200	-1.69228400
F	-3.04953600	1.23516400	-2.29039400
C	-2.28494200	-1.49997500	-0.01548400
C	-2.88537500	-1.88832300	1.18232700
C	-4.19820600	-2.35386300	1.18507900
C	-4.90992400	-2.46255700	-0.00843000
C	-4.29874700	-2.10247400	-1.20534800
C	-2.98935800	-1.61881100	-1.21280400
P	2.05852300	-0.17555300	-0.00178500
C	2.64577000	0.72385300	1.59364000
C	4.12276600	0.55762500	1.95504000
C	1.76542700	0.22520200	2.74725700
C	2.35799900	2.22382800	1.48637600
C	2.72392400	0.82305500	-1.50921400
C	2.52210100	-0.02031100	-2.77309100
C	4.18182300	1.28146600	-1.44292600
C	1.84271600	2.06221800	-1.70967300
C	2.91268300	-1.91311400	-0.04388900

C	4.38371700	-1.93984300	-0.46107600
C	2.10384400	-2.79320400	-1.01021500
C	2.79185000	-2.59817400	1.32333800
H	-2.31503100	-1.81921100	2.10716300
H	-4.66796500	-2.63858400	2.12849200
H	-5.93794000	-2.82712600	-0.00457000
H	-4.84669700	-2.18028800	-2.14558300
H	-2.54129500	-1.29183700	-2.14892600
H	4.79407600	0.91431400	1.16314000
H	4.33566600	1.15496100	2.85726500
H	4.39250000	-0.47873100	2.18682500
H	0.71231700	0.47972200	2.56762500
H	2.09058200	0.72721600	3.67386100
H	1.80565100	-0.85455800	2.90522200
H	3.04623200	2.74886900	0.81267600
H	1.32436300	2.41911700	1.17136100
H	2.48240100	2.66880800	2.48709300
H	1.48544900	-0.37460100	-2.85733200
H	2.72176700	0.61416300	-3.65093200
H	3.19990500	-0.87971800	-2.83601500
H	4.34764000	2.01458700	-0.64297200
H	4.43621500	1.78348500	-2.39120600
H	4.89521500	0.46125000	-1.30527900
H	0.78497600	1.80446100	-1.80857200
H	1.94731800	2.80392300	-0.91320800
H	2.14799000	2.55433800	-2.64733100
H	5.02048000	-1.32618600	0.18974100
H	4.74847700	-2.97781200	-0.38792800
H	4.54318700	-1.61897800	-1.49799100
H	1.05955100	-2.88347600	-0.68106900
H	2.54699000	-3.80277800	-1.01762700
H	2.10195600	-2.42795200	-2.04194900
H	3.43593100	-2.15009700	2.08949900
H	1.74979600	-2.59338700	1.66630200
H	3.11833000	-3.64475100	1.20638000
F	-0.10543100	-1.91662800	1.56689200
C	-2.06344400	2.09377700	-0.15555600
C	-3.08453500	1.56495200	0.64253200
C	-1.17430600	2.98606400	0.45398200
C	-3.18892500	1.87472300	1.99513000
H	-3.80013800	0.87611300	0.19090600
C	-1.26800000	3.30930500	1.80622800
H	-0.39135100	3.44043300	-0.15809300
C	-2.27405900	2.74348000	2.58553600
H	-3.98358100	1.42689400	2.59366700
H	-0.55279000	4.00242400	2.25416500

H	-2.34998000	2.98554500	3.64636700

Structure **VII**

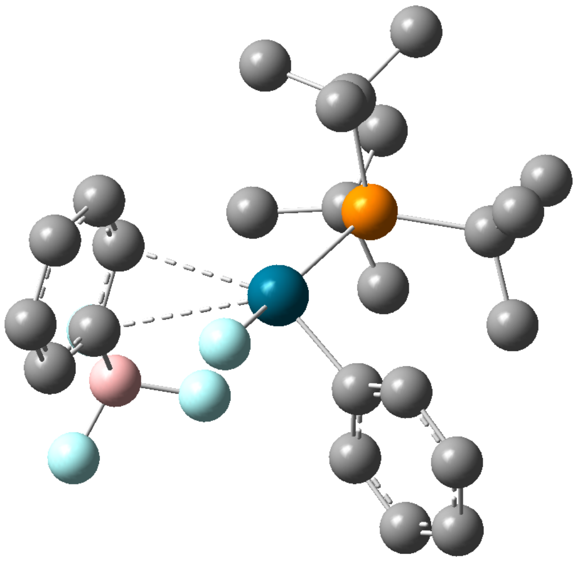

Cartesian coordinates for Structure **VII**

Sum of electronic and thermal free energies: -1829.138831

Pd	-0.44816300	0.39896200	-0.62853400
F	-2.85245400	-1.86120600	2.44566900
F	-1.76979600	0.12923300	2.14207000
B	-2.98746400	-0.58677100	1.86749200
F	-4.04973800	0.10465100	2.44103500
C	0.08892800	2.25099400	-0.18543100
C	0.60151700	3.12128900	-1.14354600
C	0.87724000	4.44307300	-0.79609200
C	0.60672000	4.90132900	0.49159400
C	0.03246400	4.03778100	1.42166500
C	-0.23806600	2.71021800	1.08833100
P	1.58193400	-0.69364200	0.05136000
C	1.87089400	-1.92050300	-1.42970200
C	3.28127200	-2.49303600	-1.59362300
C	1.48411900	-1.20180800	-2.73203100
C	0.92001900	-3.11534500	-1.30678700
C	1.56542900	-1.74096800	1.67619000
C	1.68967400	-0.78739300	2.86905200
C	2.62976600	-2.83461900	1.79738400
C	0.19112400	-2.39418300	1.83630000
C	3.18249200	0.40839100	0.14030800
C	4.43784800	-0.30816700	0.65250100
C	2.97111600	1.63524300	1.04500600
C	3.49267500	0.98205300	-1.24651200
H	0.76496800	2.77194800	-2.16350900

H	1.29170700	5.12187700	-1.54310200
H	0.82046900	5.93575400	0.76161000
H	-0.21782000	4.39887700	2.42002600
H	-0.71630500	2.03713600	1.80102900
H	3.62928700	-3.02888200	-0.70194200
H	3.26509800	-3.21972300	-2.42225000
H	4.02810500	-1.73452000	-1.85370700
H	0.44138000	-0.85259900	-2.71647500
H	1.59506400	-1.91087400	-3.56898600
H	2.11230400	-0.33247700	-2.95079500
H	1.20687000	-3.80968900	-0.50865000
H	-0.11821300	-2.80938600	-1.14402000
H	0.95082600	-3.67979300	-2.25252400
H	0.94225100	0.01646000	2.81651300
H	1.48012300	-1.35818400	3.78655100
H	2.69050400	-0.35332000	2.97940500
H	2.49436600	-3.63278700	1.05662600
H	2.52305400	-3.30473100	2.78817300
H	3.65918100	-2.46939100	1.72179100
H	-0.60945100	-1.65012000	1.87206400
H	-0.03579500	-3.13683000	1.06487400
H	0.17258400	-2.92414200	2.80155500
H	4.66595900	-1.24267800	0.13090100
H	5.29865800	0.36448900	0.50728700
H	4.38105400	-0.51834600	1.72753000
H	2.36008200	2.39569500	0.55553100
H	3.95997300	2.07984100	1.24320000
H	2.50900700	1.41535700	2.00984100
H	3.88737500	0.23938200	-1.94942300
H	2.61428000	1.46541600	-1.69151400
H	4.26594400	1.75836400	-1.13035600
F	-1.74992200	1.40495700	-1.75507000
C	-3.18642700	-0.73394200	0.25944200
C	-4.20953900	-0.07423600	-0.42285900
C	-2.35638400	-1.56947400	-0.51369000
C	-4.39726800	-0.22551500	-1.79400700
H	-4.87546300	0.57301000	0.14795300
C	-2.53654900	-1.73188400	-1.89227300
H	-1.63347800	-2.19486900	0.01057800
C	-3.56217800	-1.05272600	-2.53814600
H	-5.20533300	0.31170700	-2.29336600
H	-1.89072700	-2.40975300	-2.45680600
H	-3.70822700	-1.16410400	-3.61271100

Heterocyclic trifluoroborate π-complexes

Structure **IX**

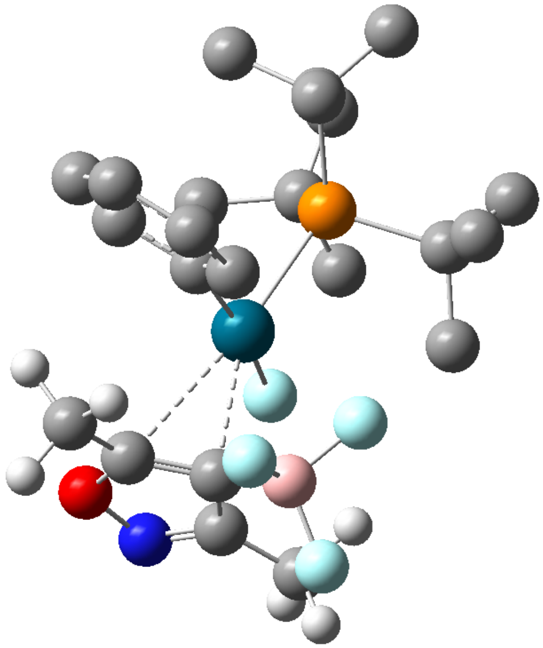

Cartesian coordinates for structure **IX**

Sum of electronic and thermal free energies: -1921.566936

Pd	-0.40517700	-0.20265000	-0.38872700
C	-2.72452700	-0.72710900	-0.04158000
C	-3.05856300	0.01989400	-1.15939300
C	-0.61008000	1.77364400	-0.17933600
C	-1.02952300	2.40661900	0.99529600
C	-1.24424600	3.78300200	1.01968900
C	-1.03688200	4.55732900	-0.12115700
C	-0.62078800	3.93921800	-1.29709500
C	-0.41211300	2.55946000	-1.32250900
P	1.95522800	-0.23241300	0.04129700
C	2.13635500	-1.18216300	1.70604800
C	3.51846400	-1.79256300	1.95409400
C	1.08075200	-2.29578900	1.75970300
C	1.80419200	-0.24841400	2.87400400
C	2.98211200	1.41175800	0.20116200
C	3.12291100	2.06288700	-1.18049400
C	4.37694800	1.23552500	0.81588700
C	2.26127500	2.46553700	1.05715900
C	2.81812500	-1.26552500	-1.35891200
C	4.34204700	-1.14466500	-1.45305300
C	2.17744600	-0.80171800	-2.67502100
C	2.51133100	-2.76516000	-1.24308300
H	-1.22055300	1.81084500	1.88535500

H	-1.58523800	4.25477000	1.94228900
H	-1.20449500	5.63435200	-0.09463400
H	-0.46188400	4.52945300	-2.20134200
H	-0.10740500	2.08168700	-2.25900400
H	4.32680700	-1.05361800	1.95795600
H	3.51008600	-2.26964900	2.94721300
H	3.77271300	-2.57266500	1.22819800
H	0.07042700	-1.87099100	1.81311000
H	1.25522300	-2.87494700	2.68149400
H	1.10672000	-2.97692900	0.90669800
H	2.57941400	0.50516500	3.06162500
H	0.83186800	0.24331600	2.73659900
H	1.72404000	-0.86274500	3.78440200
H	2.14284200	2.21435900	-1.65046700
H	3.57149300	3.05989800	-1.04304900
H	3.76687500	1.51179100	-1.87335000
H	4.32035500	1.03995400	1.89419000
H	4.92658400	2.18320600	0.69549000
H	4.98149300	0.44893500	0.35606600
H	1.42011700	2.91226200	0.52524300
H	1.88613000	2.09418600	2.01343300
H	2.98404500	3.26925400	1.27315900
H	4.84409300	-1.50140800	-0.54347700
H	4.67720500	-1.79419500	-2.27742900
H	4.70697900	-0.13684900	-1.67734600
H	1.10338100	-1.02879800	-2.67086600
H	2.64282100	-1.35218500	-3.50882200
H	2.30763700	0.26898900	-2.87246200
H	3.02418200	-3.24378100	-0.40000400
H	1.43085000	-2.93013000	-1.17491200
H	2.88926000	-3.24778000	-2.15937800
F	-0.25530500	-2.18085000	-0.92907600
B	-2.90789300	-0.37501300	1.55870200
F	-3.44304700	0.90938200	1.70619600
F	-3.77518700	-1.32148200	2.10595800
F	-1.67126900	-0.44213700	2.25954900
C	-3.45942200	1.43571700	-1.34999400
H	-4.51104700	1.45843900	-1.66845400
H	-3.35984700	1.98169500	-0.40893900
H	-2.86120500	1.93331900	-2.12324700
O	-3.26966800	-0.76502200	-2.23146000
N	-3.14253100	-2.10990700	-1.84014500
C	-2.82354800	-2.07368000	-0.57774100
C	-2.66590900	-3.33577800	0.18683400
H	-1.72081700	-3.31213100	0.73986500
H	-3.48065000	-3.43269900	0.91351600

H	-2.66114500	-4.19406400	-0.49422800

Structure **X**

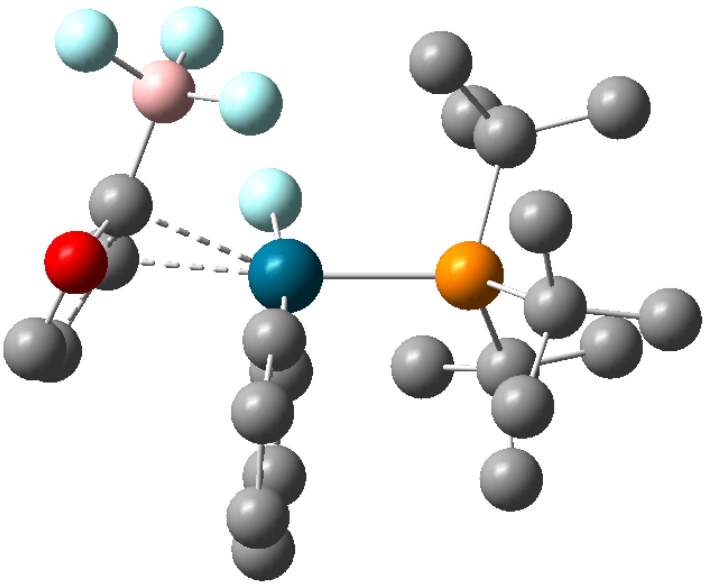

Cartesian coordinates for Structure **X**

Sum of electronic and thermal free energies: -1827.018056

Pd	-0.46783000	-0.29516200	0.68011000
C	-2.52581500	-0.91962400	1.58621800
C	-2.87597000	-1.01677100	0.23660000
F	-3.80208200	-2.54022500	-1.46247300
F	-1.88976200	-1.41180700	-2.02465800
B	-2.58317100	-2.07536900	-0.98406700
F	-1.80164600	-3.11970700	-0.49115500
C	-1.12344000	1.45559500	-0.02632800
C	-1.04773000	2.55269100	0.84108000
C	-1.56709600	3.79567000	0.48092200
C	-2.18204800	3.96194500	-0.75711300
C	-2.28356100	2.87106900	-1.61846200
C	-1.75896200	1.62952900	-1.26128600
P	1.80208600	-0.00898500	-0.05108800
C	2.86658500	0.57591300	1.44093100
C	4.35967000	0.24959500	1.35528700
C	2.28273000	-0.05909000	2.70848400
C	2.73176300	2.09009700	1.62590600
C	2.20720300	1.16786500	-1.53899400
C	1.57959400	0.56657500	-2.80262600
C	3.70293600	1.41234300	-1.77672100
C	1.56646900	2.55699400	-1.39654500
C	2.39672700	-1.78303800	-0.57242200

C	3.68691800	-1.84306000	-1.39484700
C	1.25746800	-2.42571700	-1.37612300
C	2.59984800	-2.67862300	0.65675700
H	-1.95863800	-1.67470100	2.12531400
H	-0.58807000	2.43295900	1.82637400
H	-1.49318600	4.63602800	1.17362900
H	-2.58891300	4.93199700	-1.04455700
H	-2.78078700	2.98391800	-2.58322300
H	-1.87187900	0.77291300	-1.92590000
H	4.84234200	0.68815200	0.47381900
H	4.86106100	0.66826100	2.24351200
H	4.55825100	-0.82731900	1.35269000
H	1.25407500	0.28496200	2.88181200
H	2.90469700	0.24524500	3.56692200
H	2.23091800	-1.14926400	2.67482500
H	3.29501300	2.66455300	0.87996200
H	1.68417400	2.41928800	1.60466400
H	3.14294400	2.35377500	2.61360500
H	0.52089300	0.31514700	-2.65079400
H	1.63038700	1.31853000	-3.60602500
H	2.09762300	-0.32833900	-3.16361400
H	4.13412500	2.06416100	-1.00525100
H	3.81592200	1.94226900	-2.73652500
H	4.30887700	0.50460400	-1.83387100
H	0.47820700	2.51540100	-1.46544500
H	1.82675500	3.08292200	-0.47547000
H	1.92994200	3.17109700	-2.23671800
H	4.54906000	-1.40671400	-0.87234900
H	3.92015200	-2.90586600	-1.56623600
H	3.60407000	-1.37689900	-2.38355400
H	0.34794200	-2.56276100	-0.77865300
H	1.59458300	-3.42471500	-1.69782600
H	0.97714100	-1.87077300	-2.27582700
H	3.48344700	-2.40865200	1.24938500
H	1.69950800	-2.67586400	1.28462800
H	2.76634500	-3.70328900	0.28730200
F	0.06722400	-1.95785400	1.80301500
C	-3.29503300	0.16537000	2.14393600
H	-3.27717000	0.52555000	3.16598000
O	-3.78723400	-0.03426300	-0.02406800
C	-4.02128100	0.66201900	1.11944100
H	-4.70265100	1.49886000	1.02442200

Section 5: Spectroscopic Data

^{1}H NMR Spectra

^{1}H NMR (500 MHz) spectrum for 2a

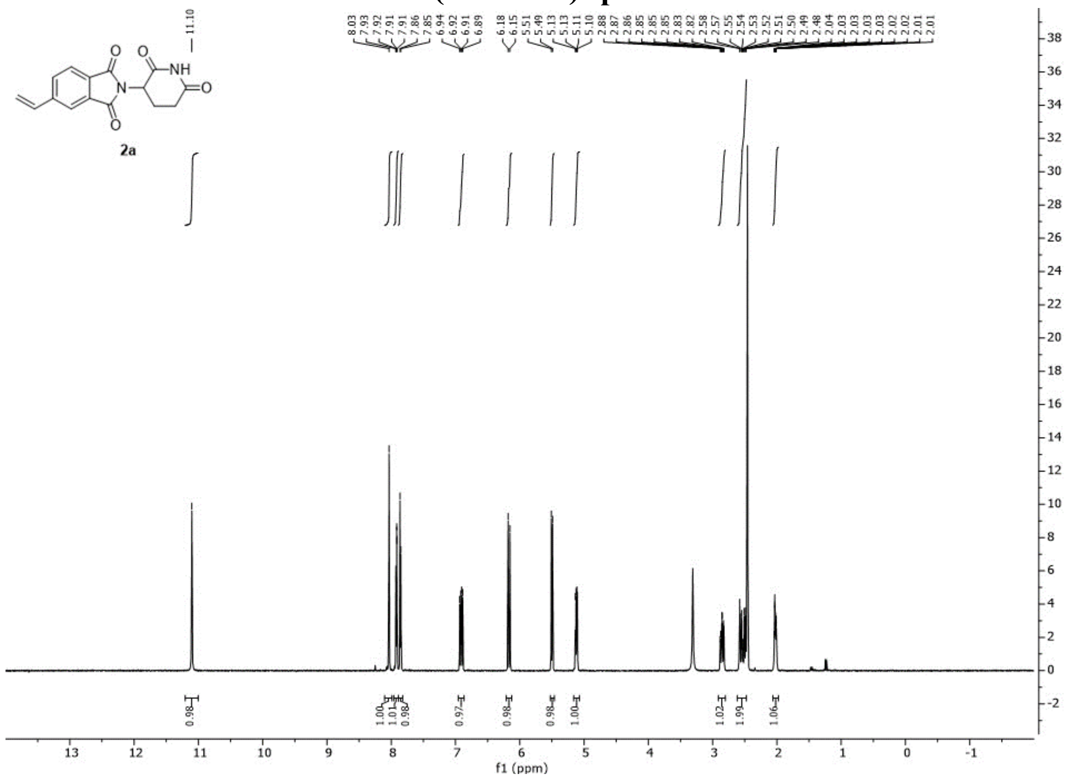

^{1}H NMR (500 MHz) spectrum for 2b

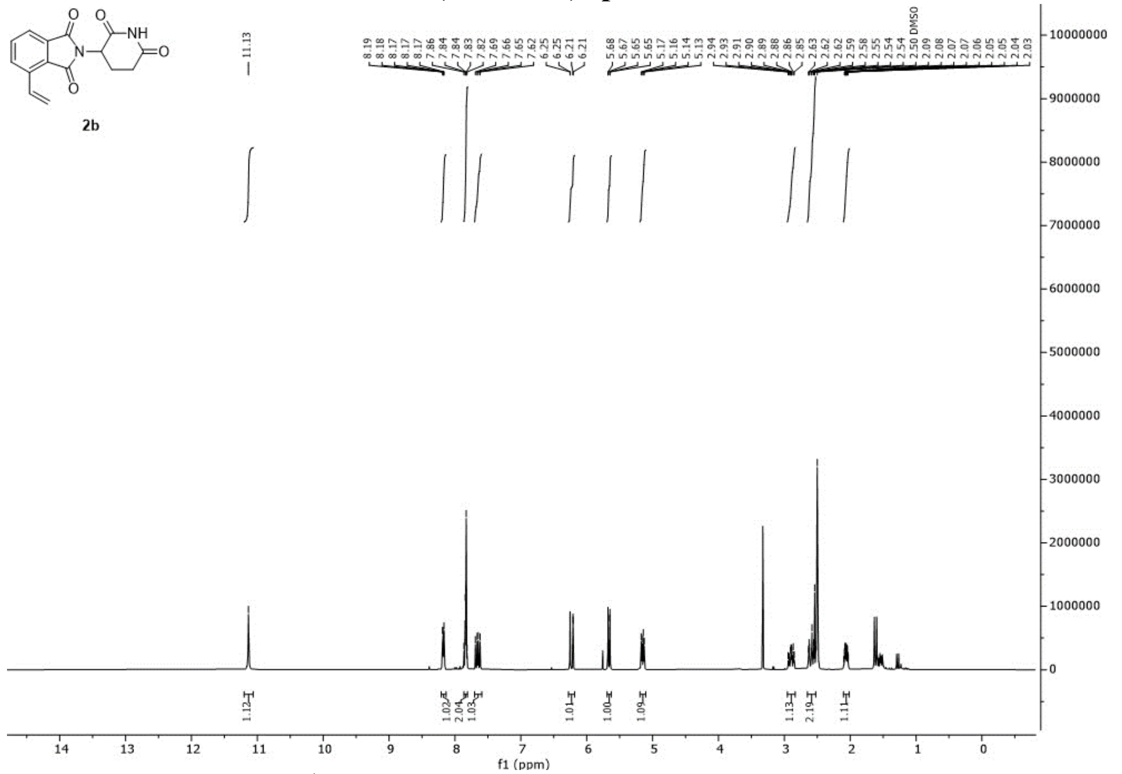

^{1}H NMR (400 MHz) spectrum for 2c

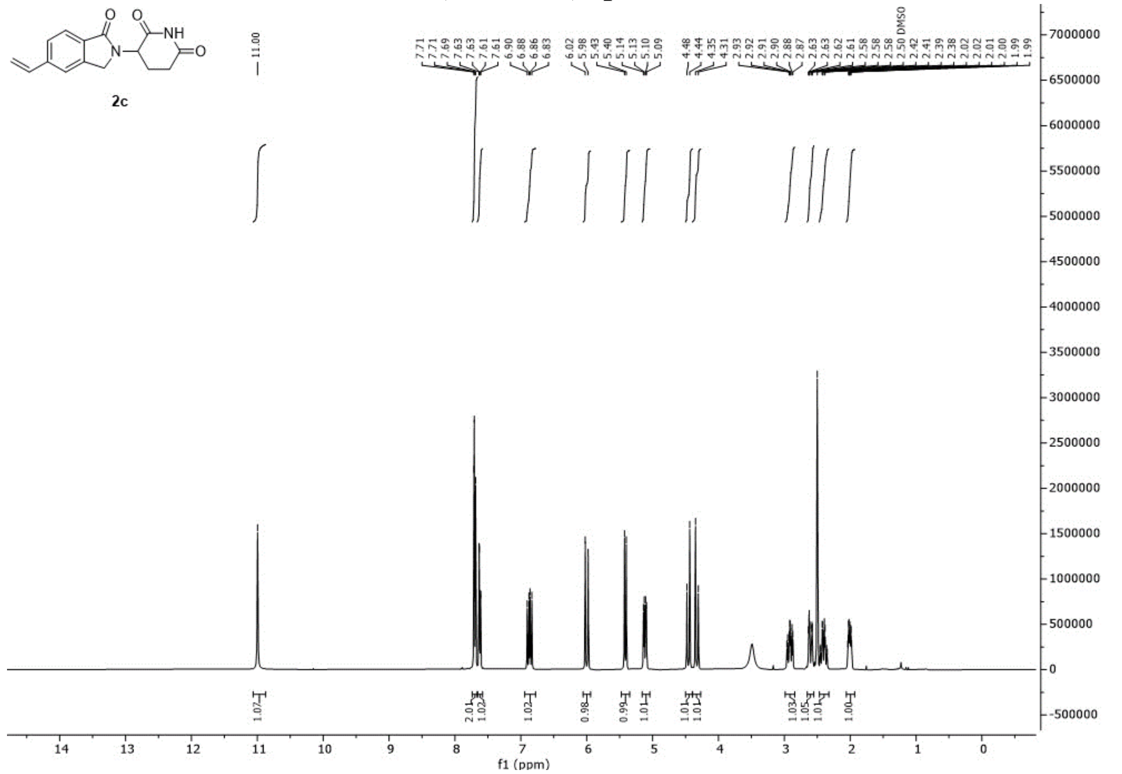

^{1}H NMR (600 MHz) spectrum for 2d

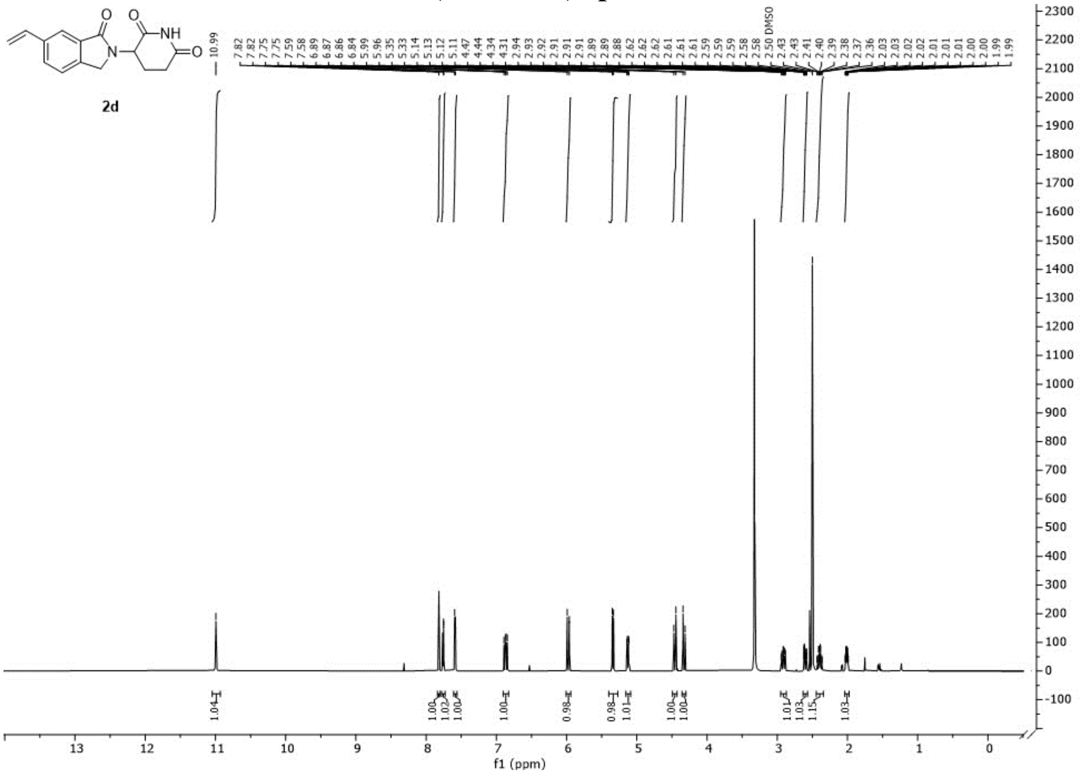

^{1}H NMR (400 MHz) spectrum for 2e

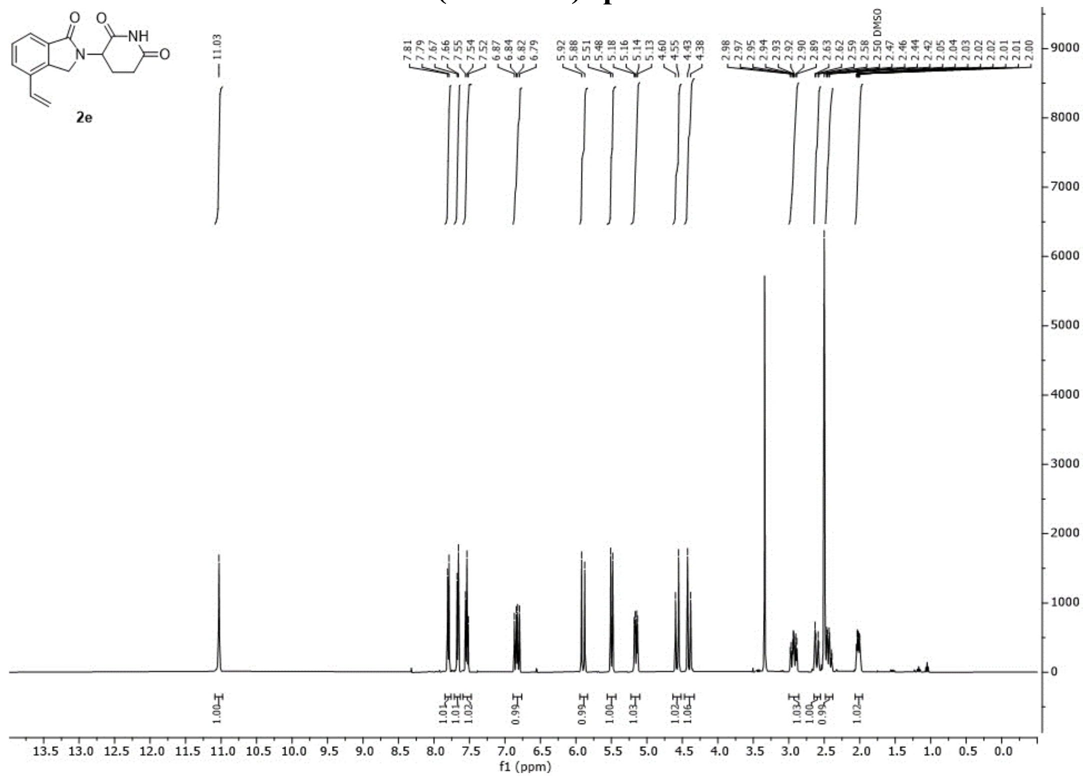

^{1}H NMR (500 MHz) spectrum for 2f

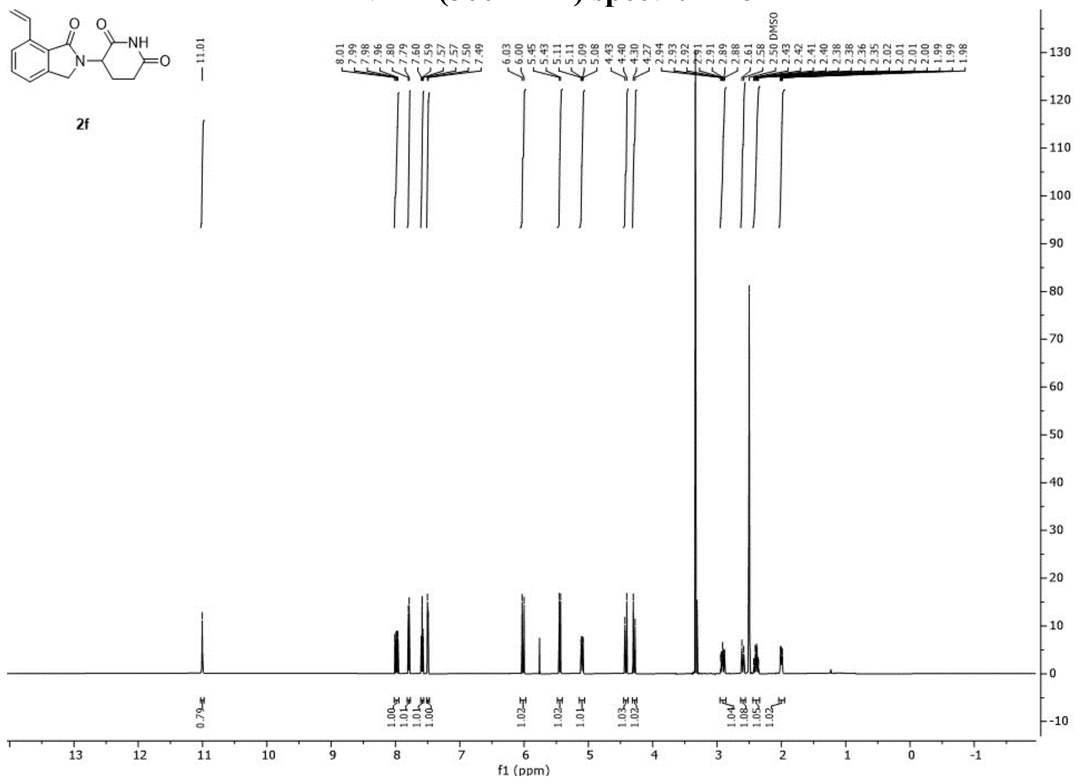

^{1}H NMR (400 MHz) spectrum for 5a

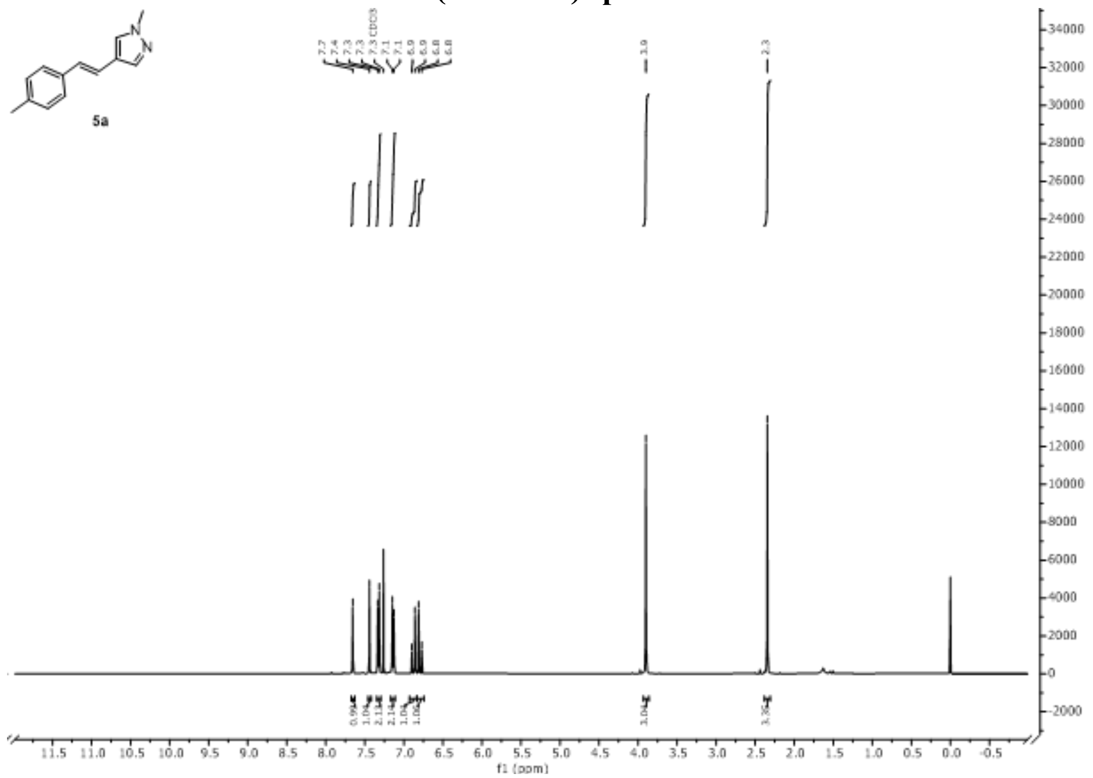

^{1}H NMR (600 MHz) spectrum for 5b

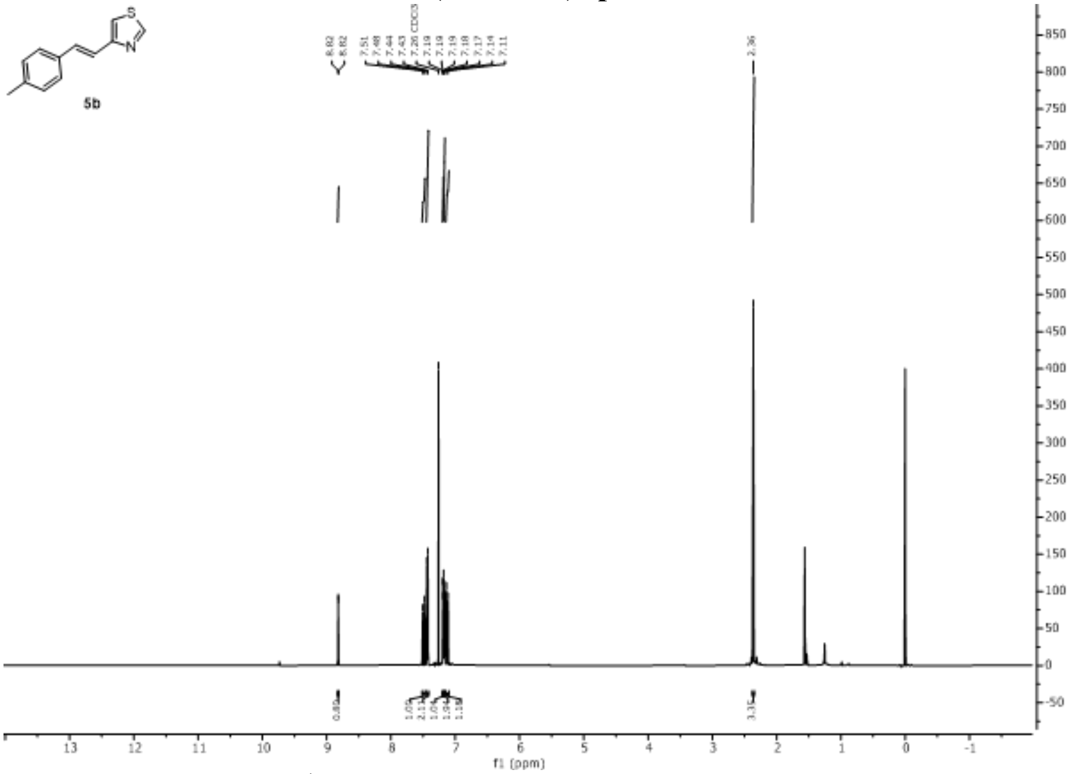

^{1}H NMR (400 MHz) spectrum for 5c

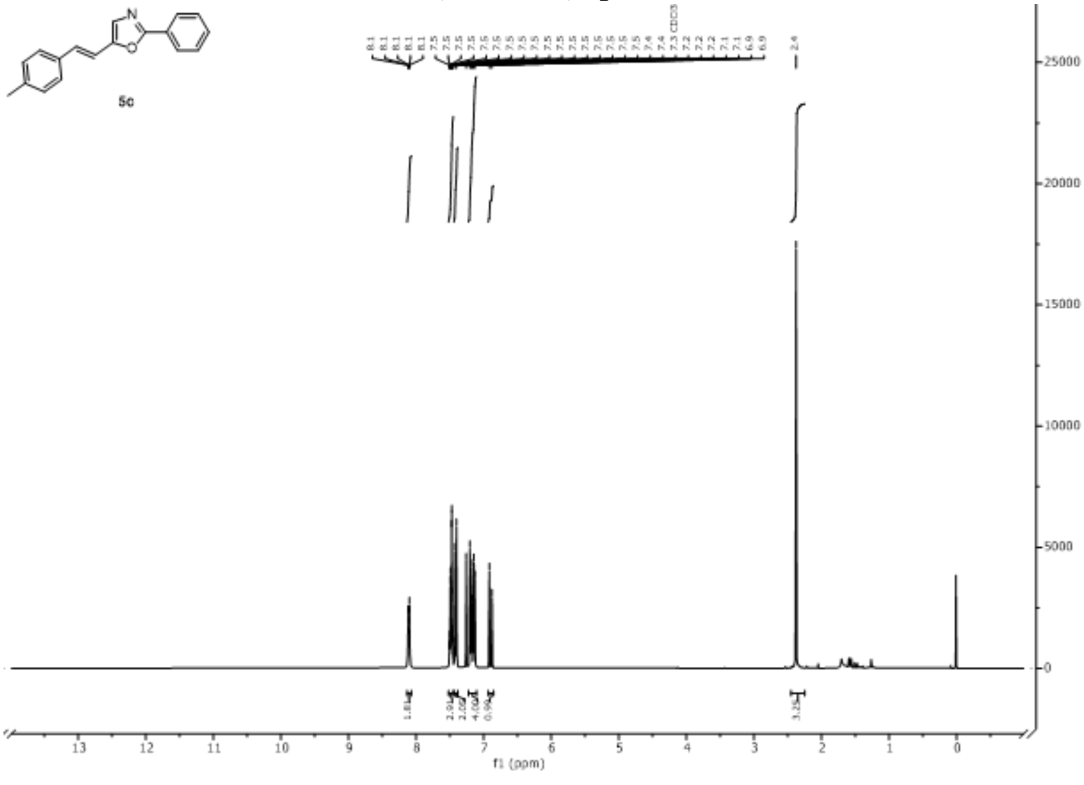

^{1}H NMR (400 MHz) spectrum for 5d

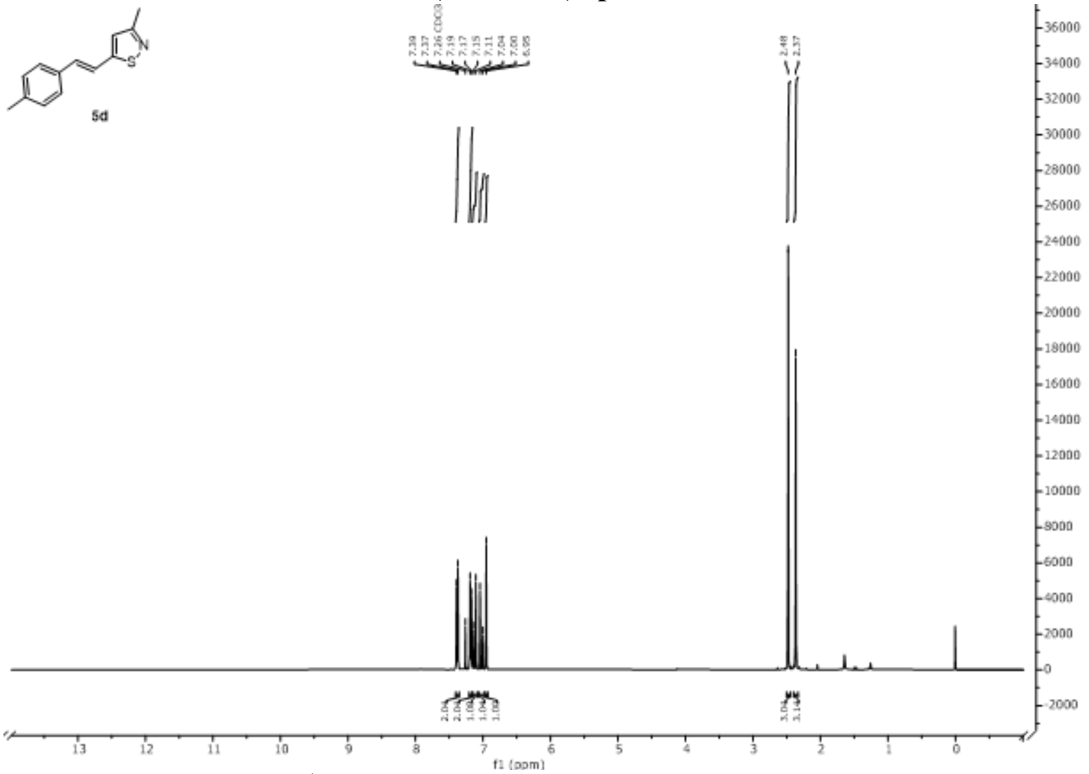

^{1}H NMR (400 MHz) spectrum for 5f

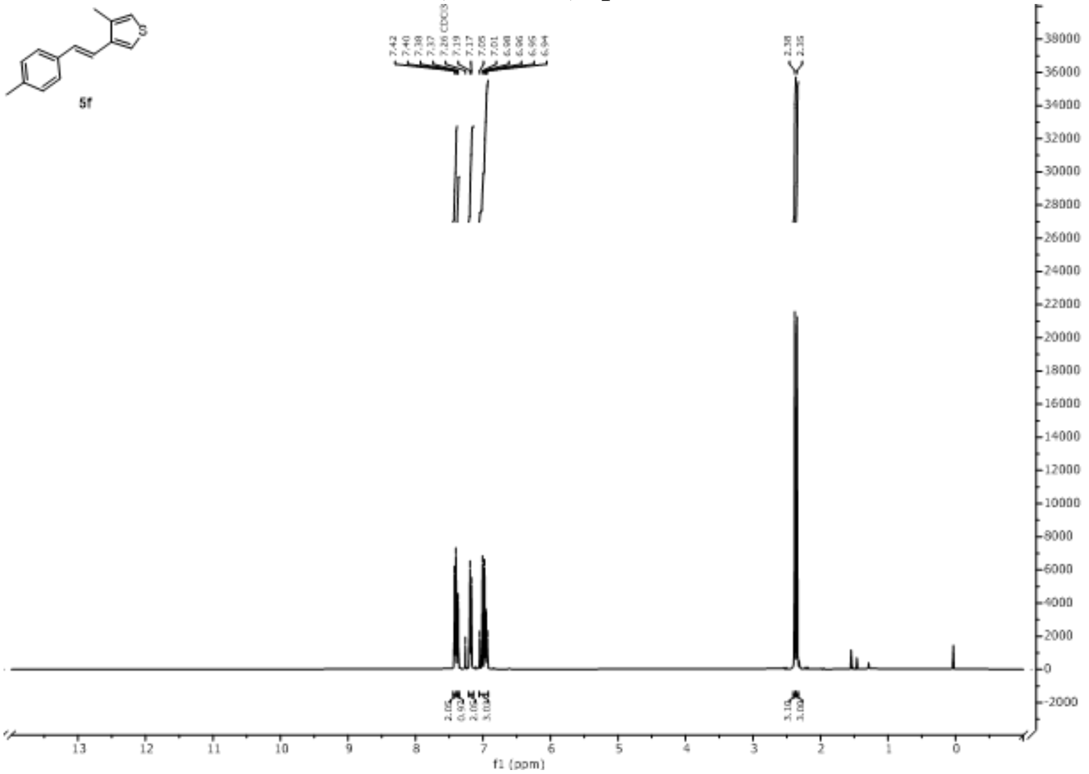

^{1}H NMR (400 MHz) spectrum for 5g

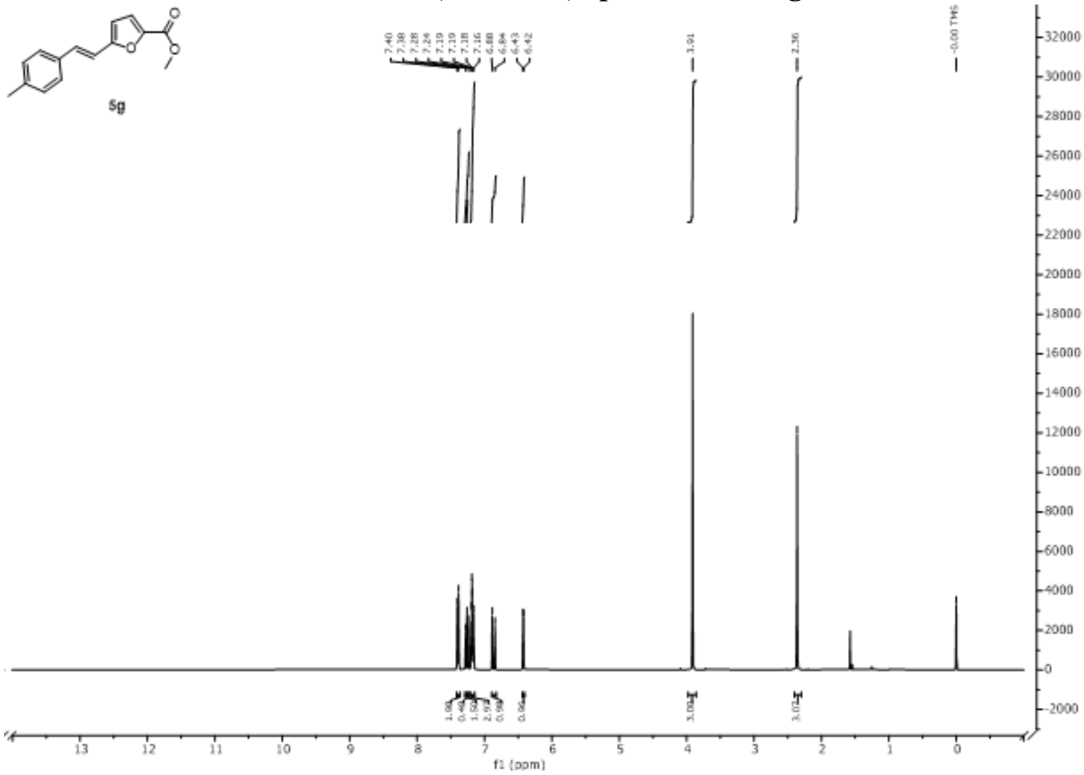

^{1}H NMR (600 MHz) spectrum for 5h

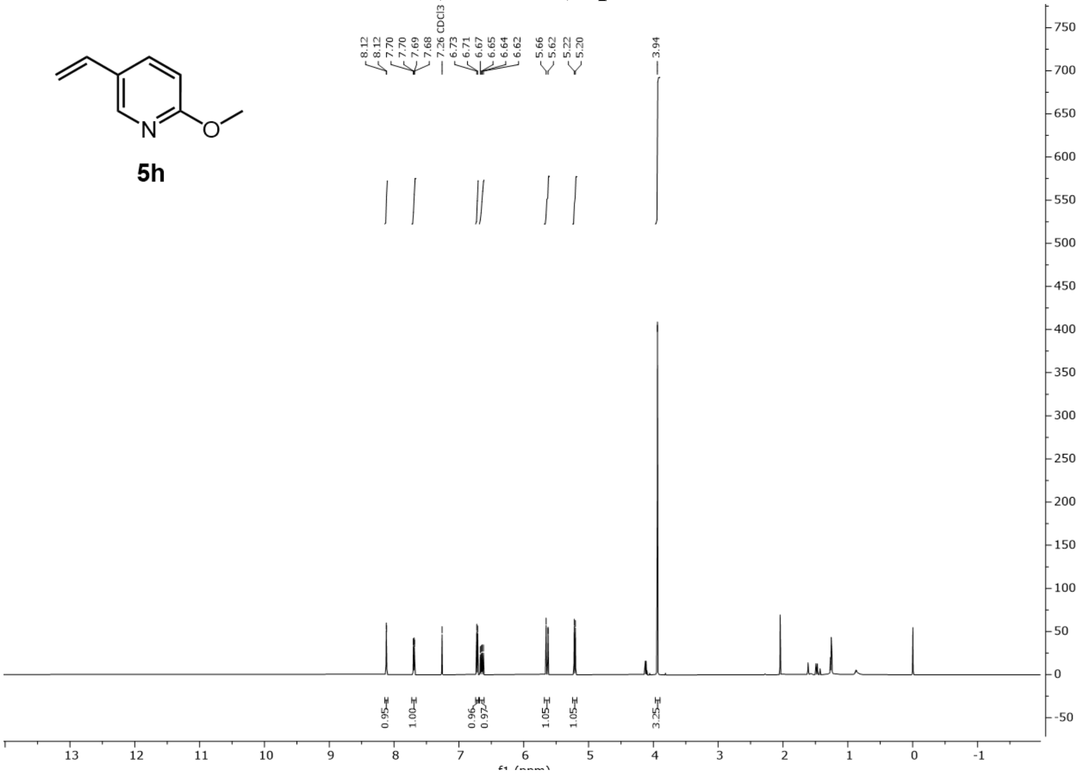

^{1}H NMR (600 MHz) spectrum for 6a

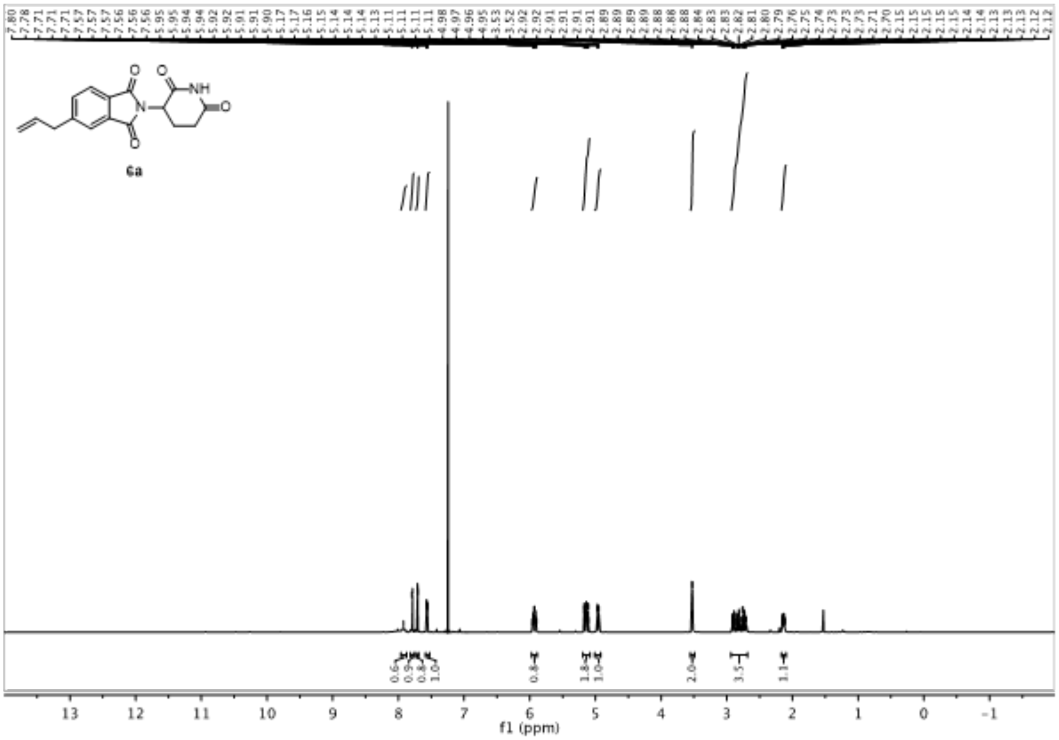

^{1}H NMR (400 MHz) spectrum for 6b

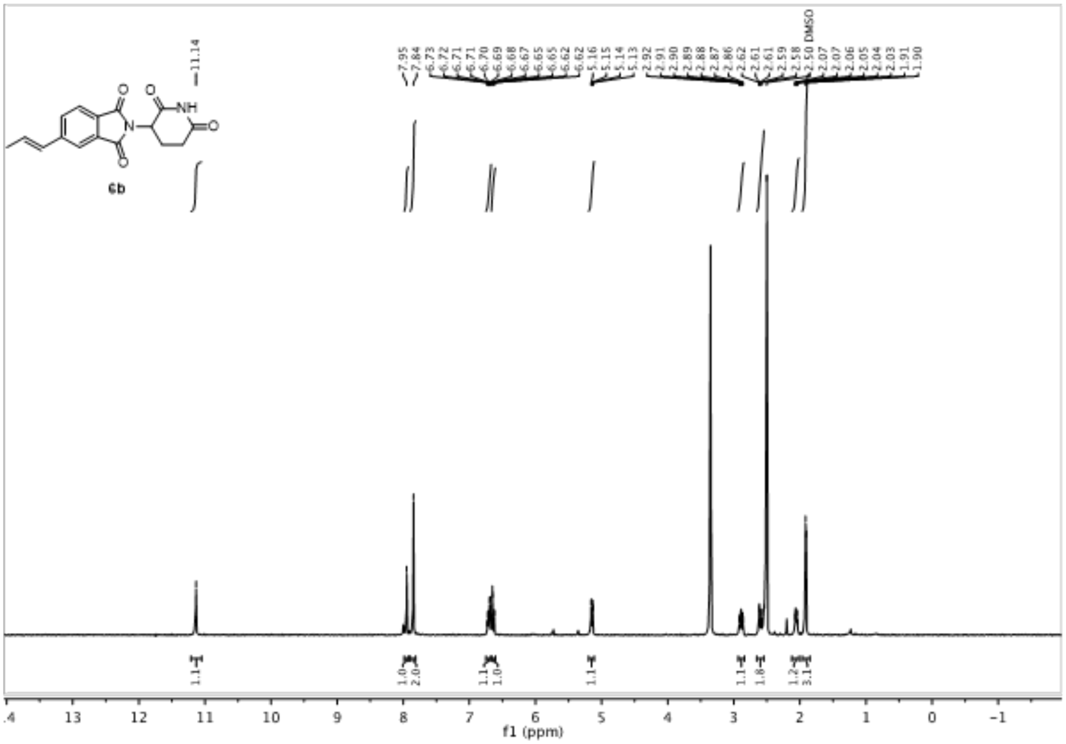

^{1}H NMR (400 MHz) spectrum for 6c

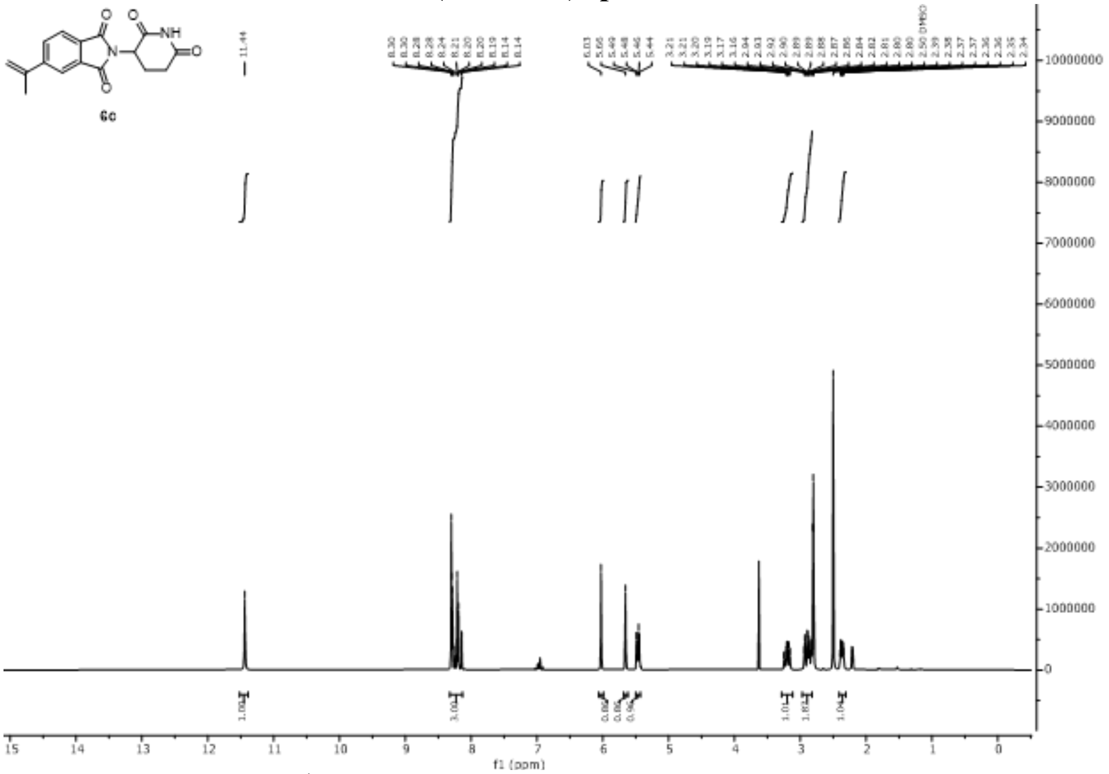

^{1}H NMR (600 MHz) spectrum for 6d

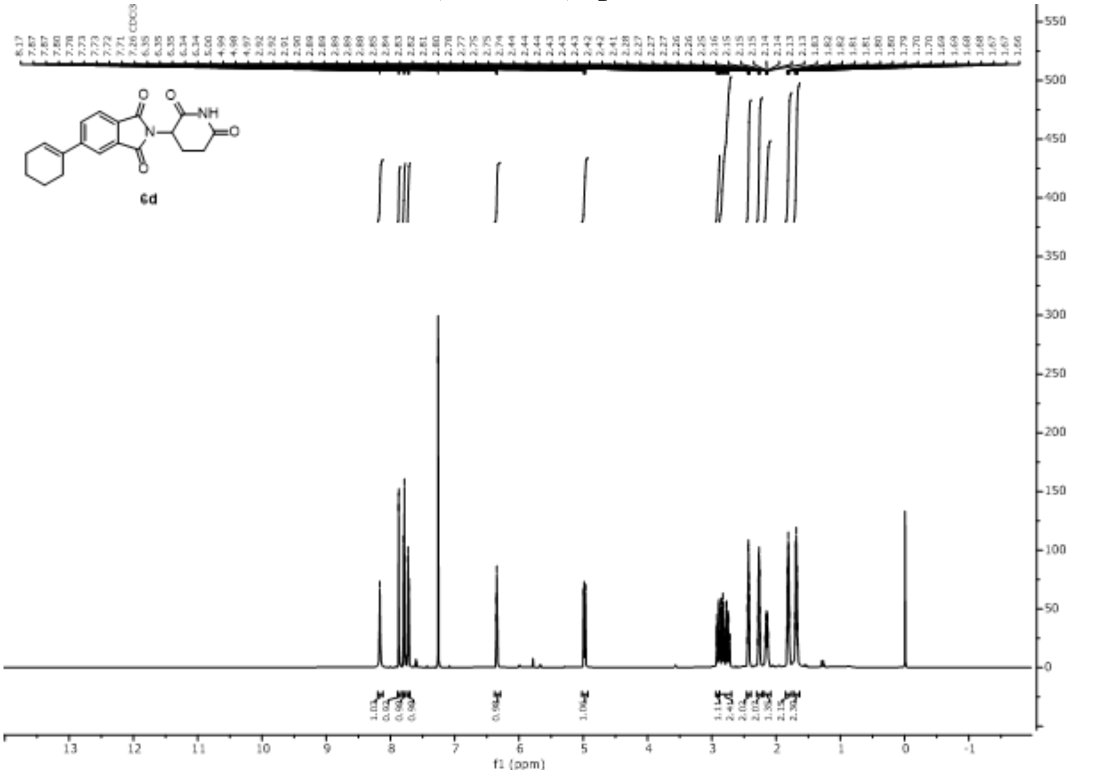

^{1}H NMR (600 MHz) spectrum for 6e

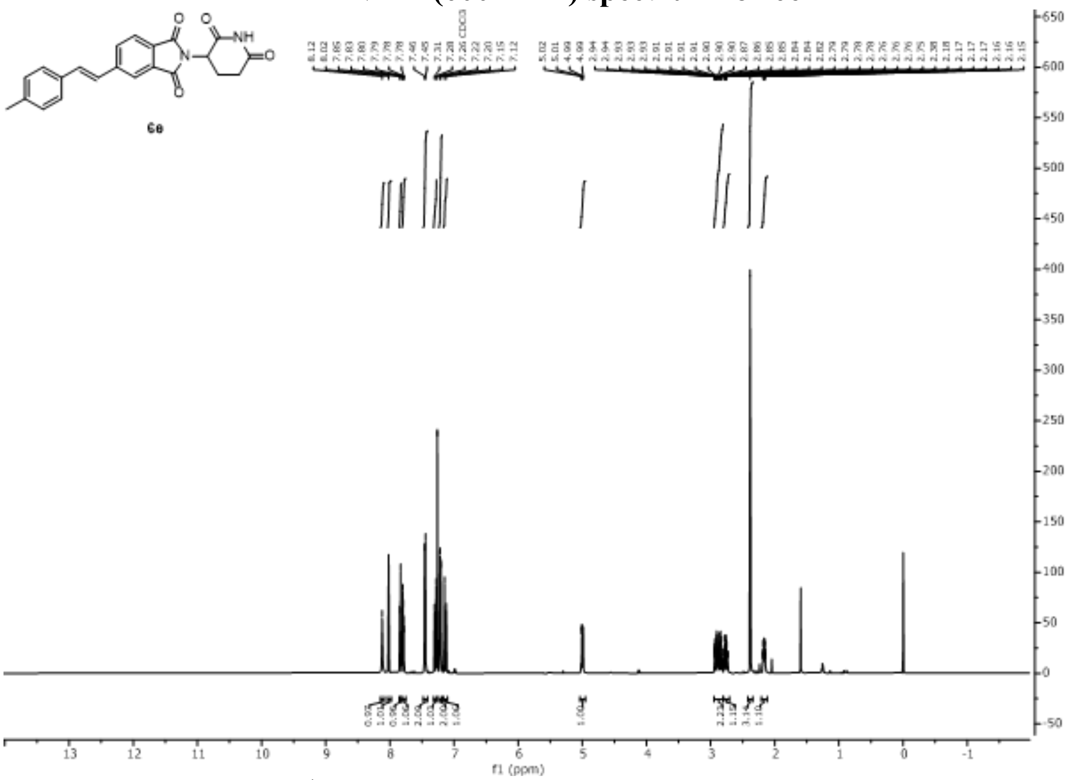

^{1}H NMR (600 MHz) spectrum for 6f

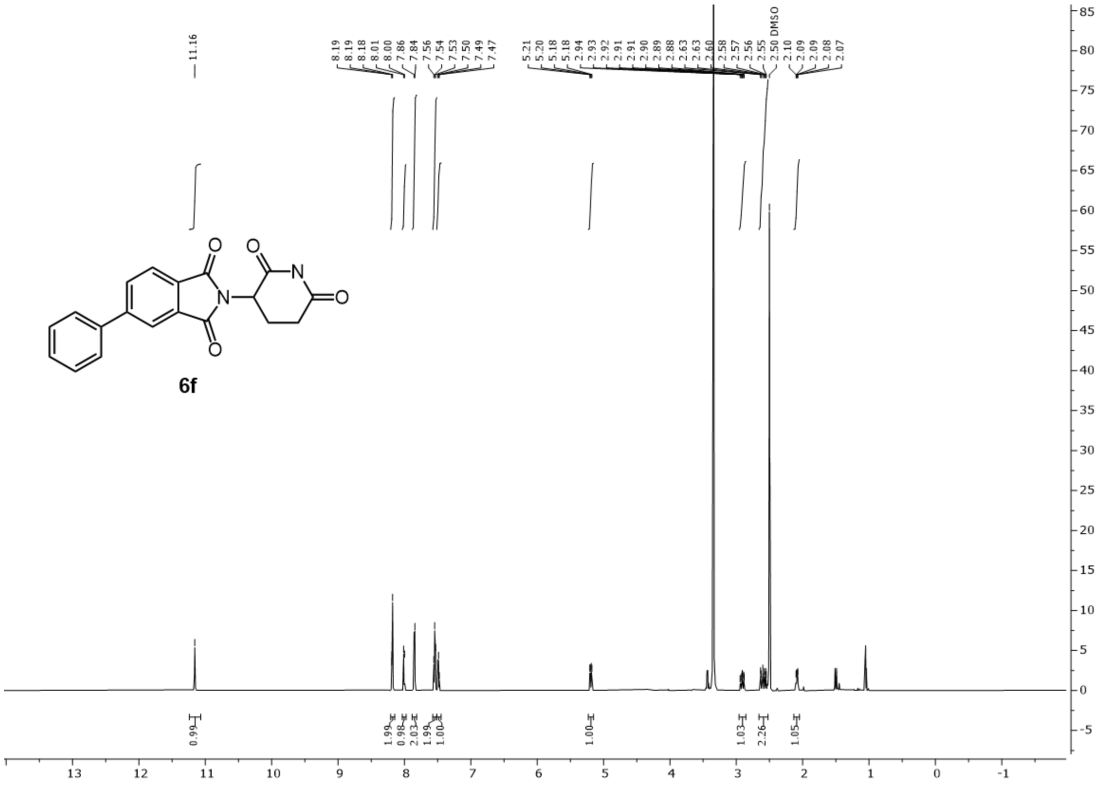

^{1}H NMR (600 MHz) spectrum for 6g

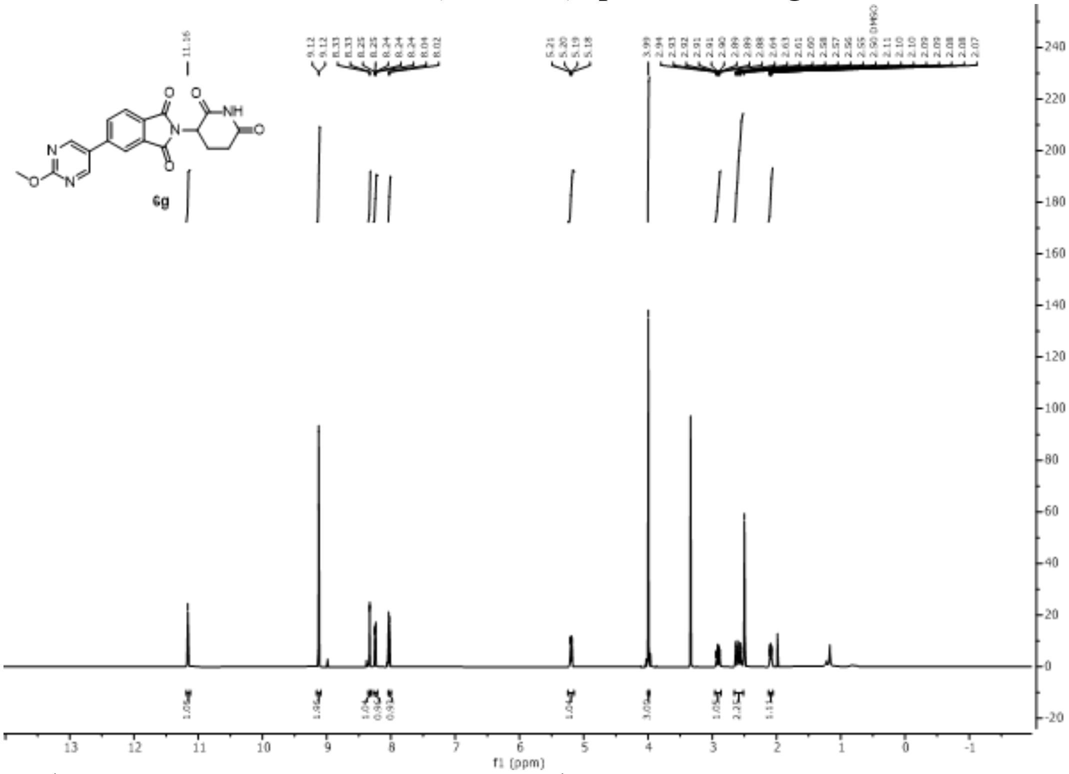

^{1}H NMR (600 MHz) spectrum for 6a ^{1}H NMR (600 MHz) spectrum for 6h

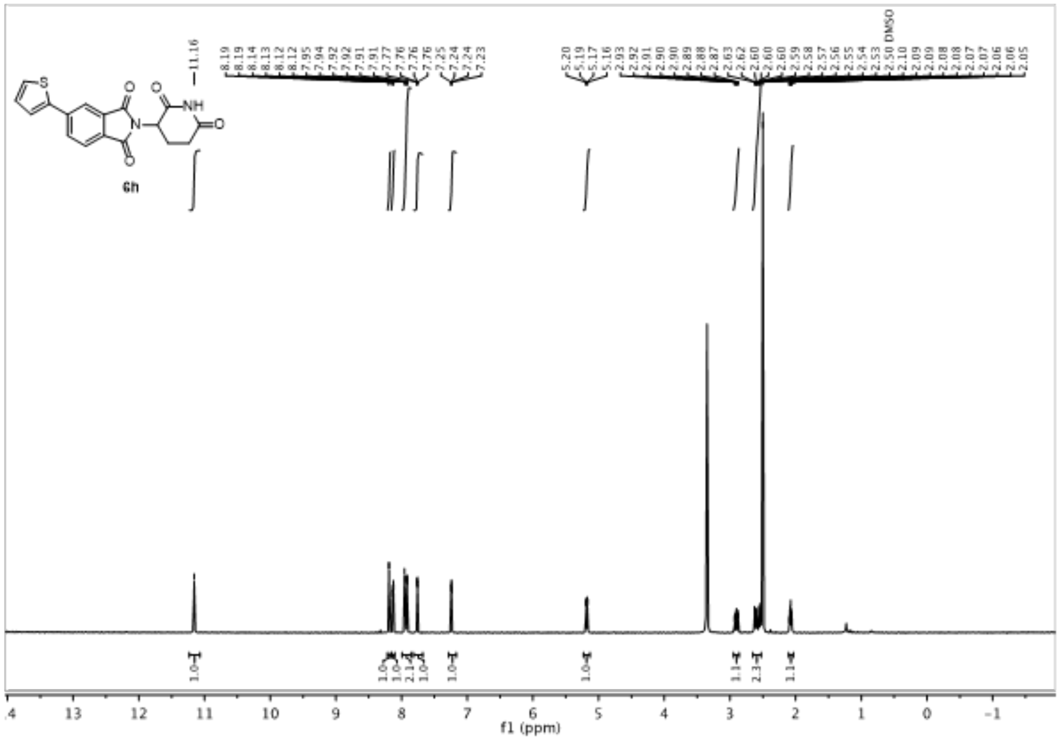

^{1}H NMR (400 MHz) spectrum for 6i

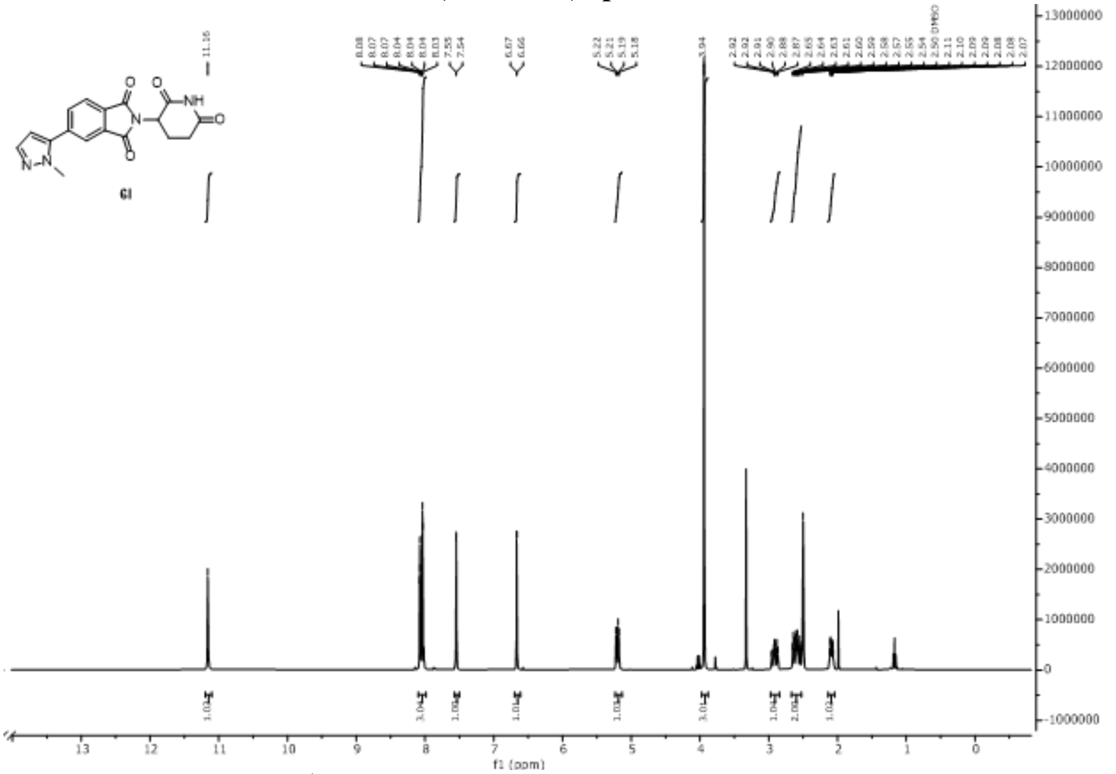

^{1}H NMR (600 MHz) spectrum for 6j

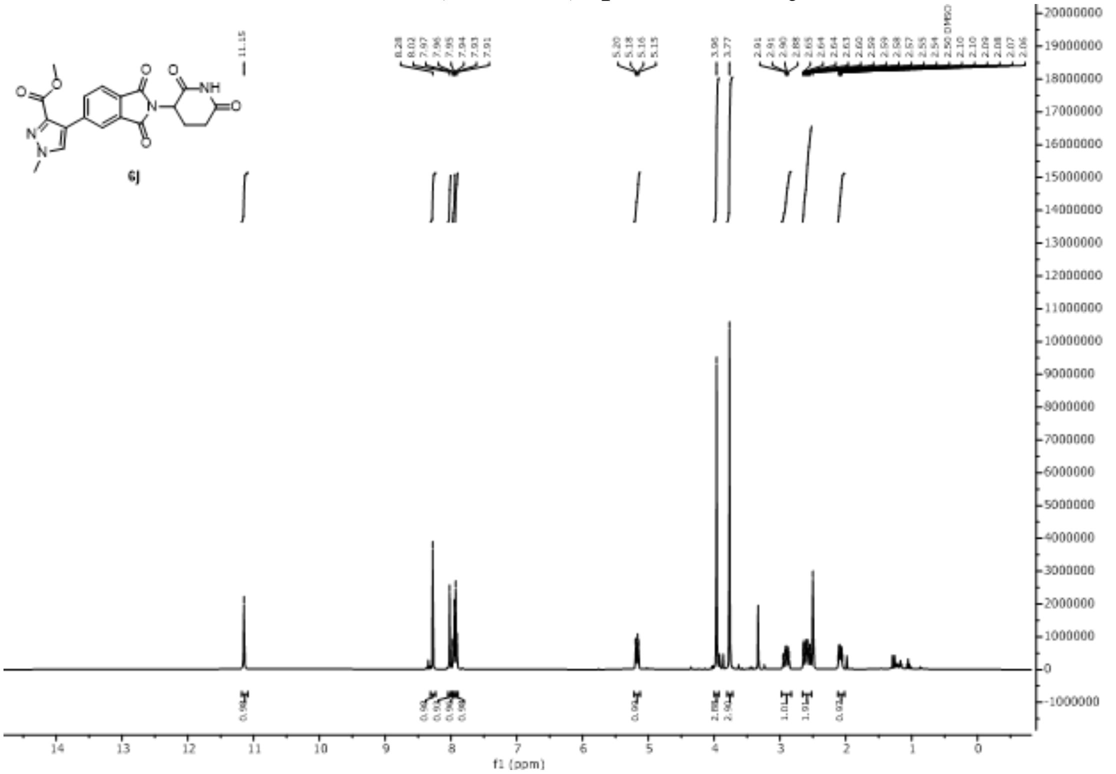

^{1}H NMR (600 MHz) spectrum for 6k

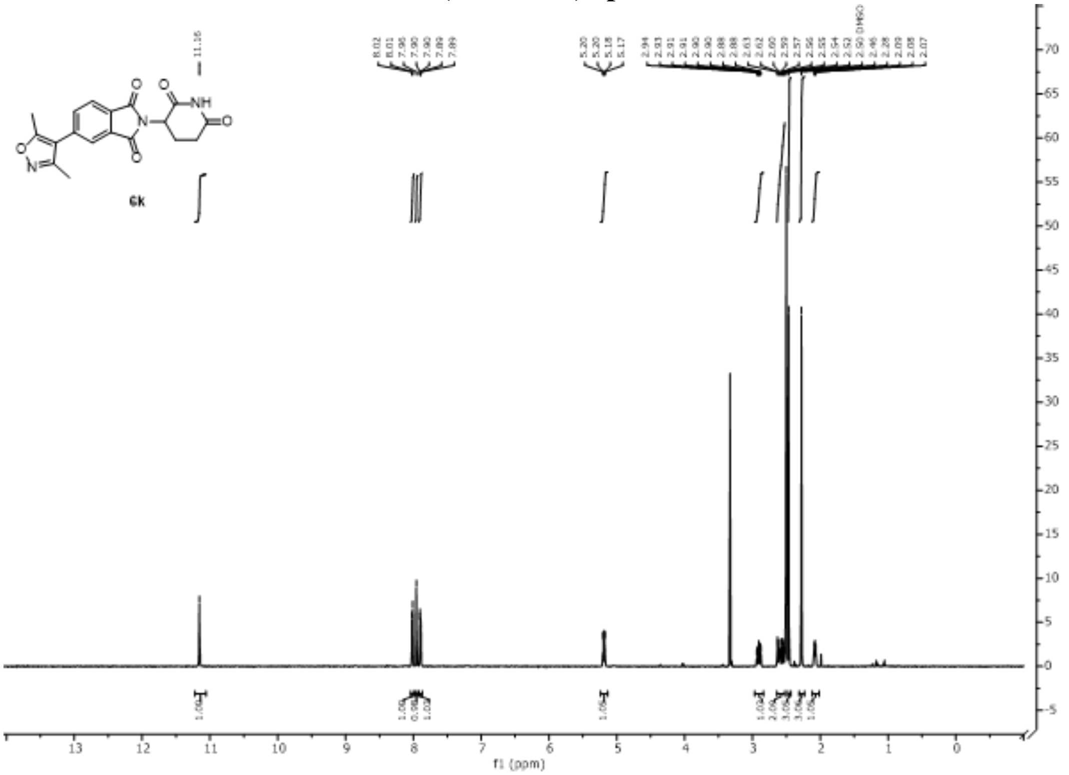

\

^{1}H NMR (600 MHz) spectrum for 6l

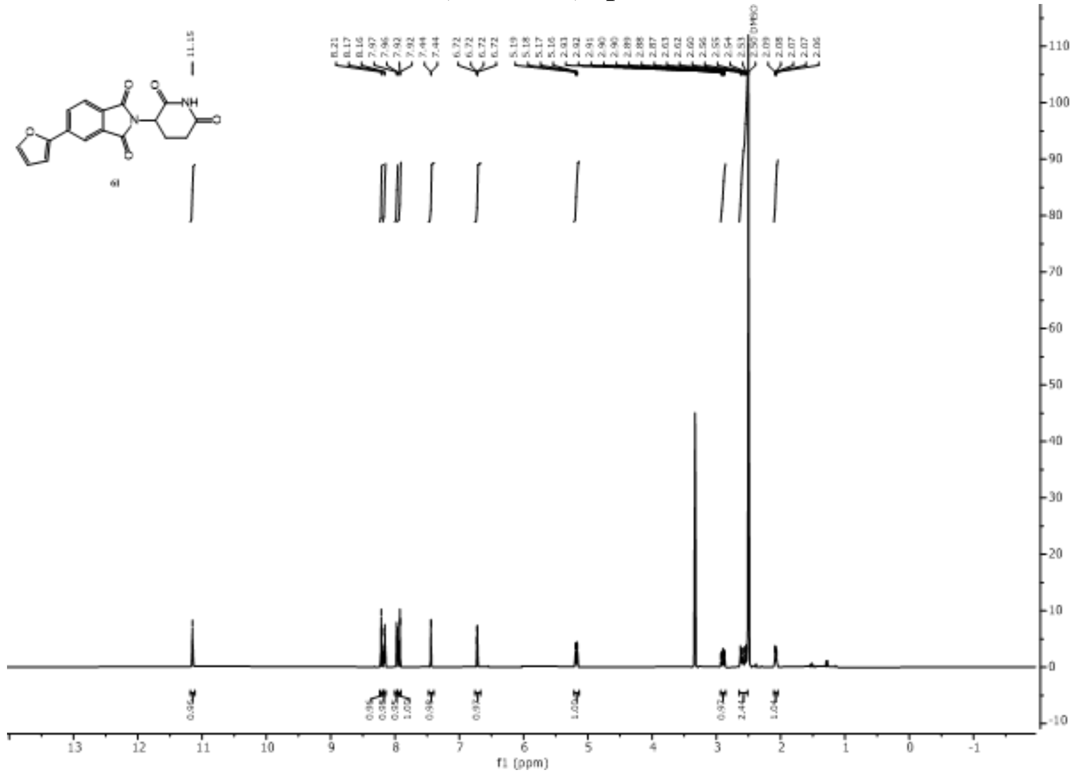

$^{13}C\{^1H\}$ NMR Spectra

$^{13}C\{^1H\}$ NMR (101 MHz) spectrum for 2a

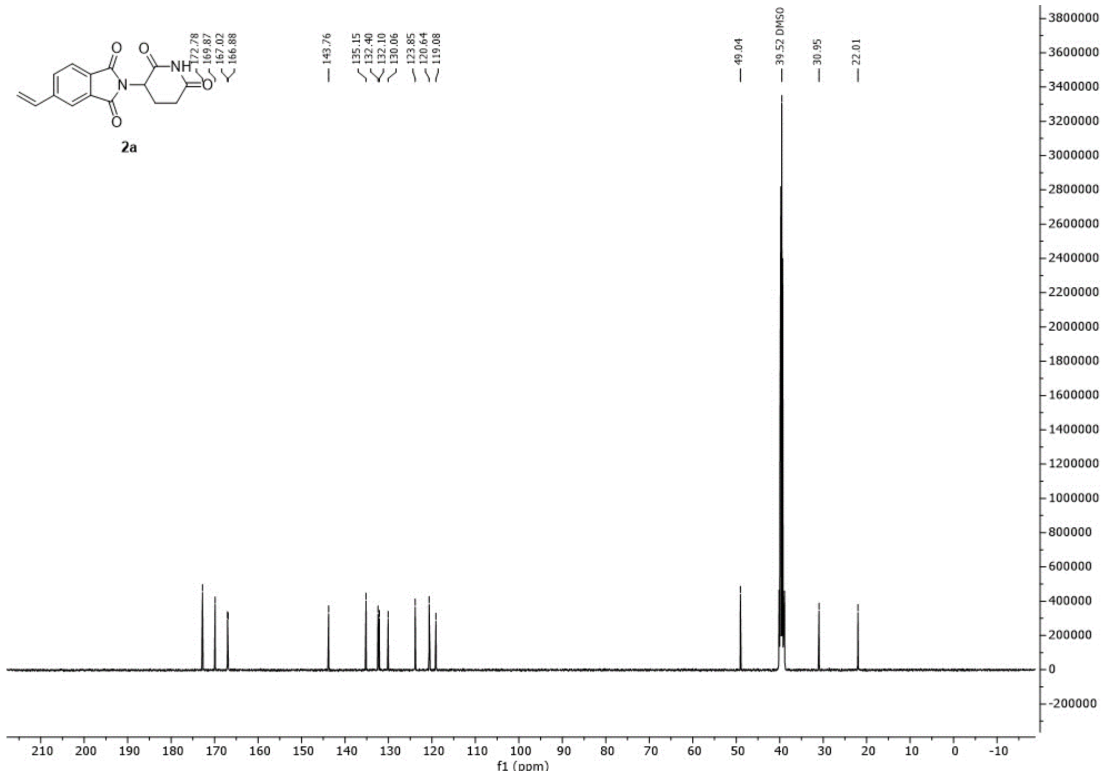

$^{13}C\{^1H\}$ NMR (101 MHz) spectrum for 2b

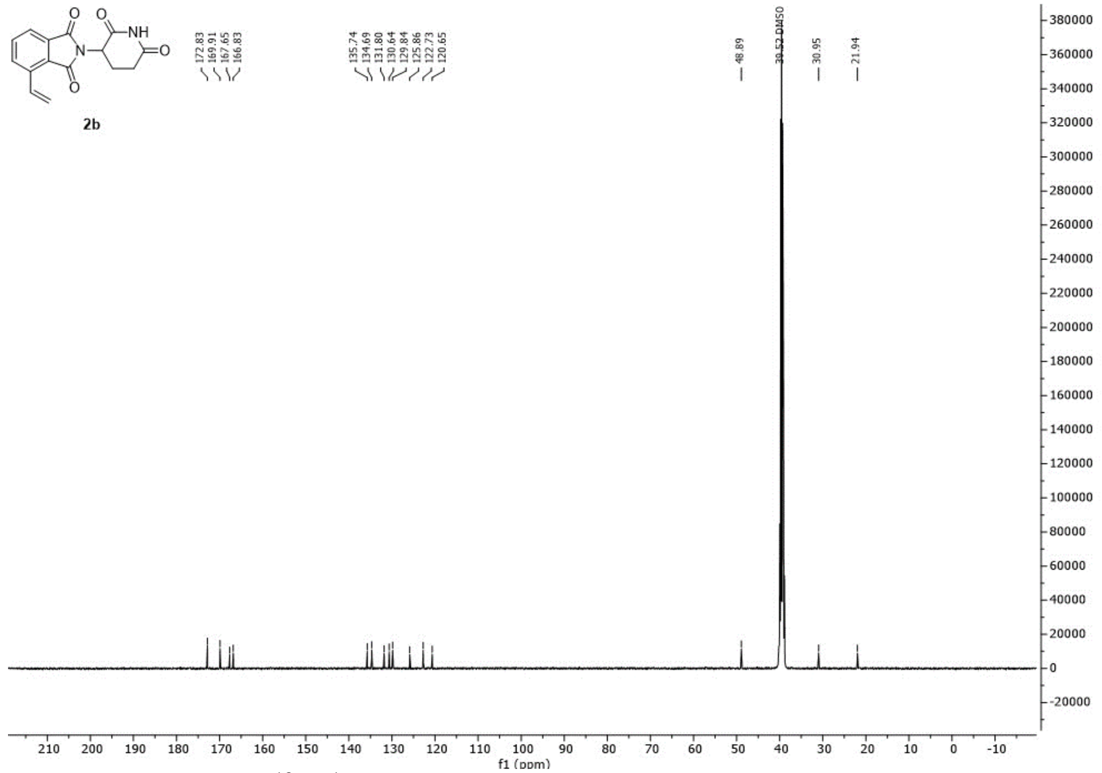

$^{13}C\{^1H\}$ NMR (101 MHz) spectrum for 2c

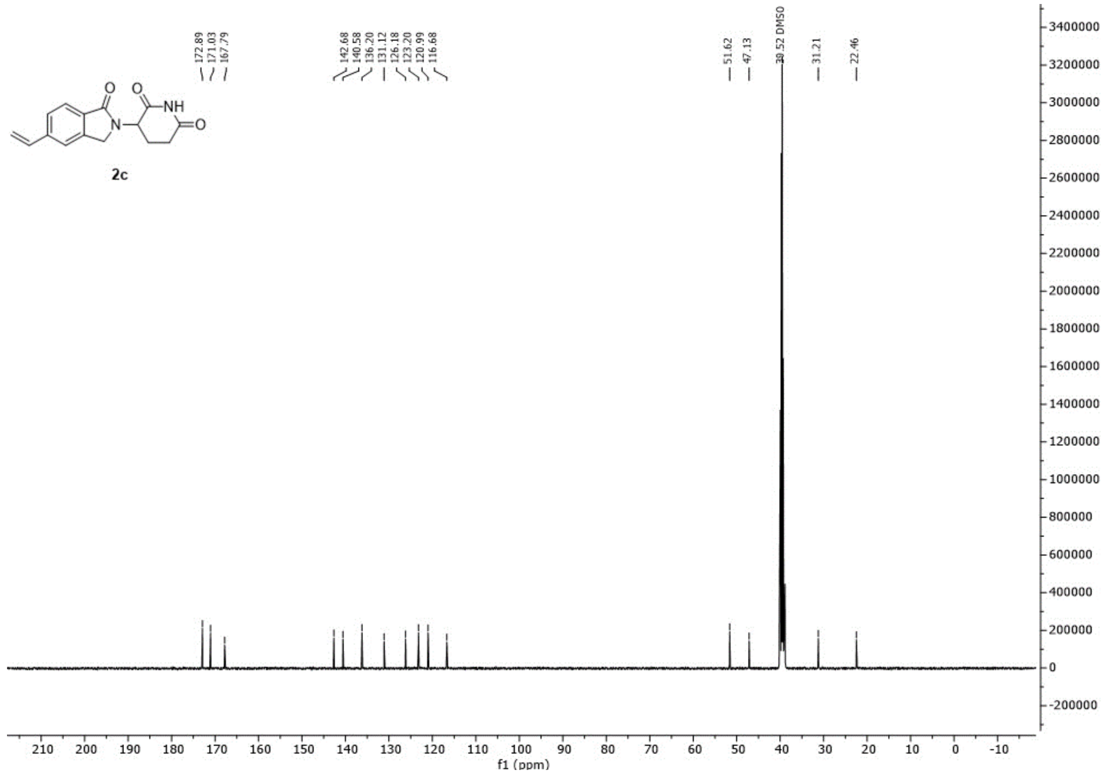

$^{13}C\{^1H\}$ NMR (151 MHz) spectrum for 2d

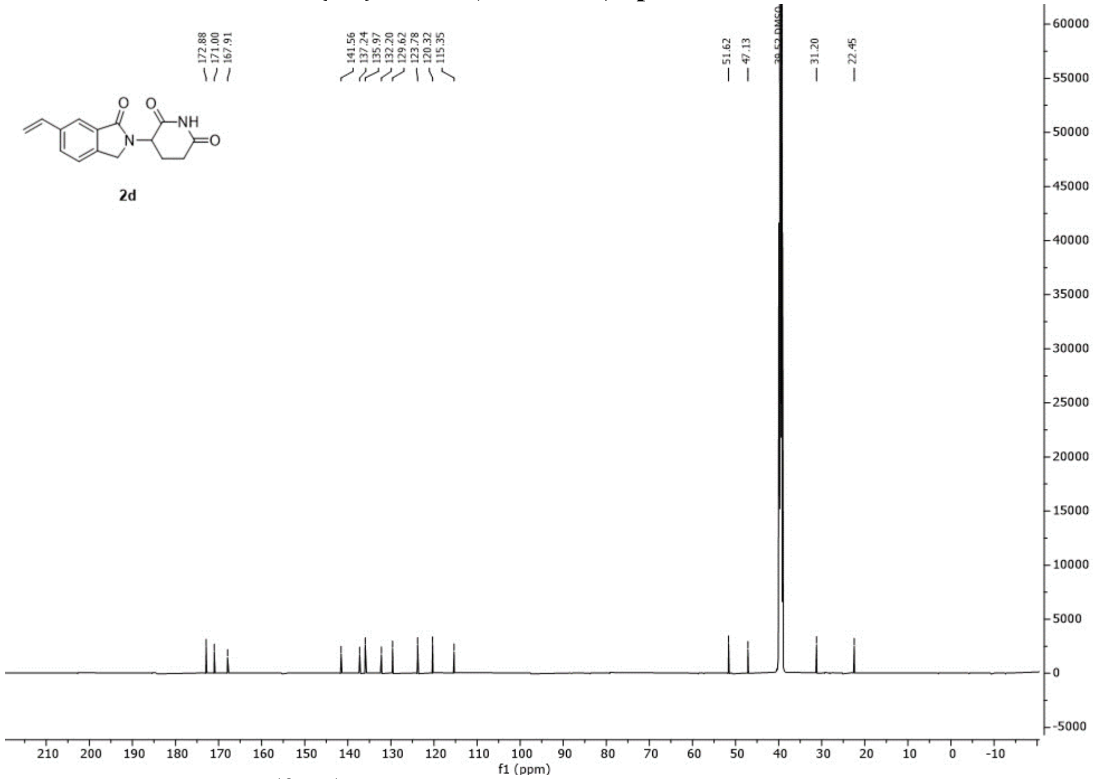

$^{13}C\{^1H\}$ NMR (101 MHz) spectrum for 2e

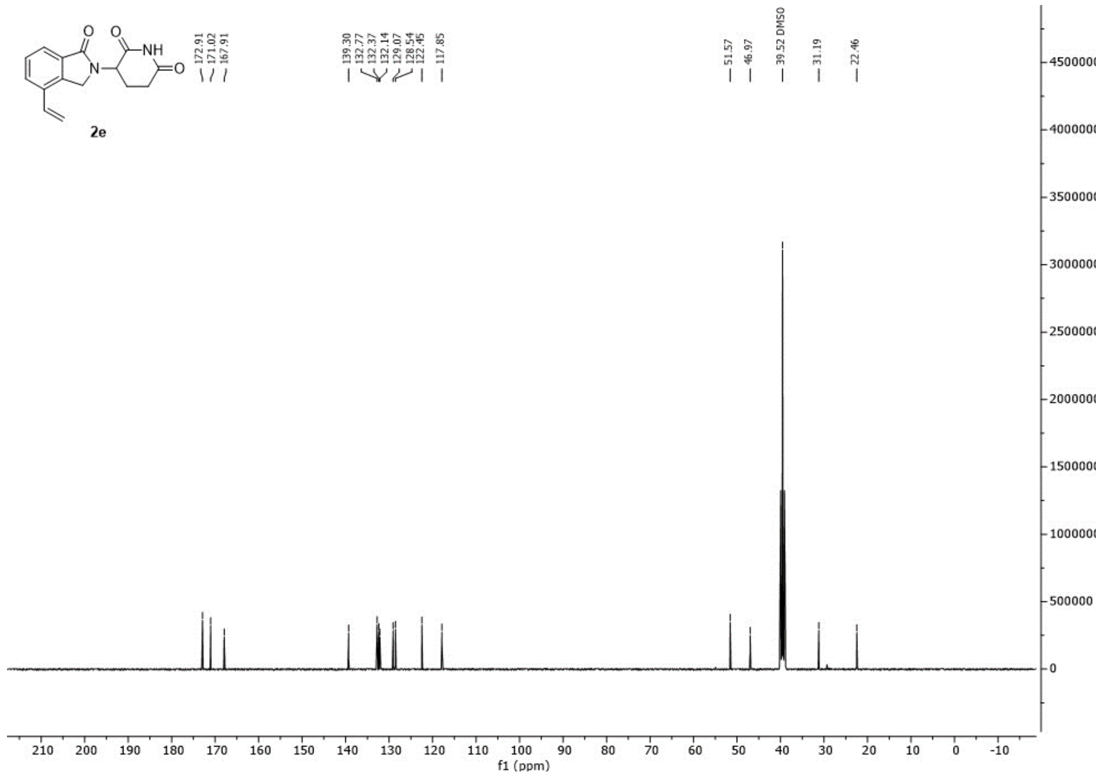

$^{13}C\{^1H\}$ NMR (101 MHz) spectrum for 2f

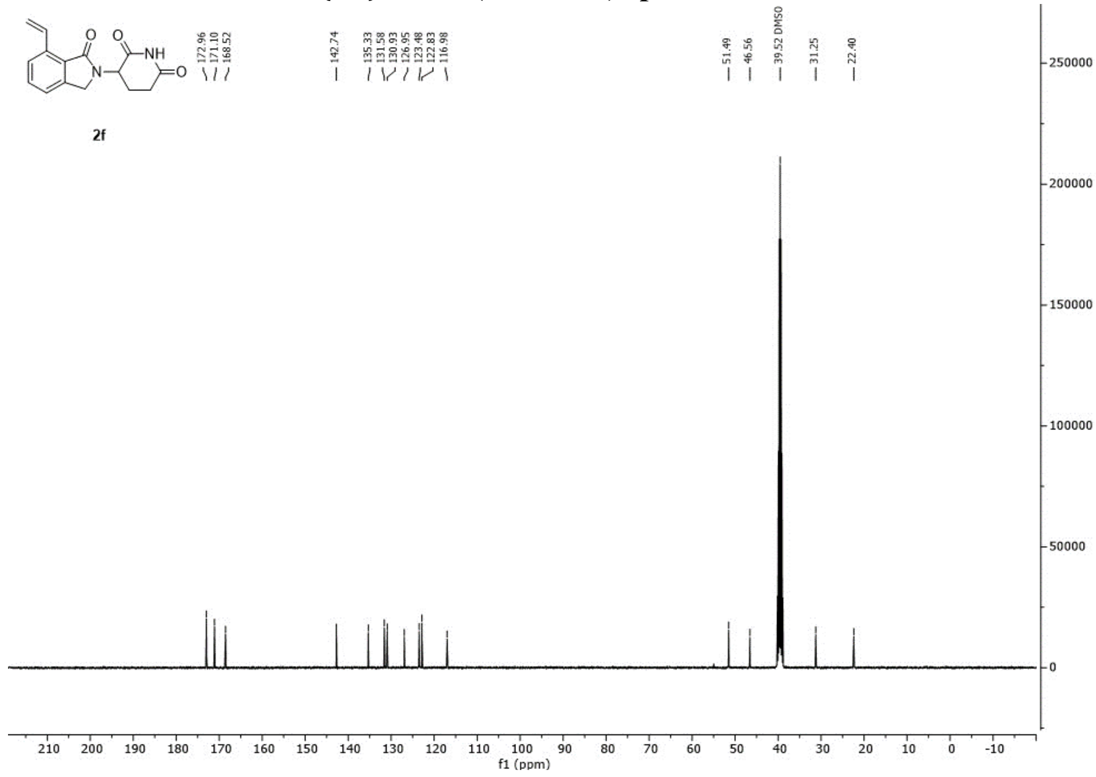

$^{13}C\{^1H\}$ NMR (101 MHz) spectrum for 5a

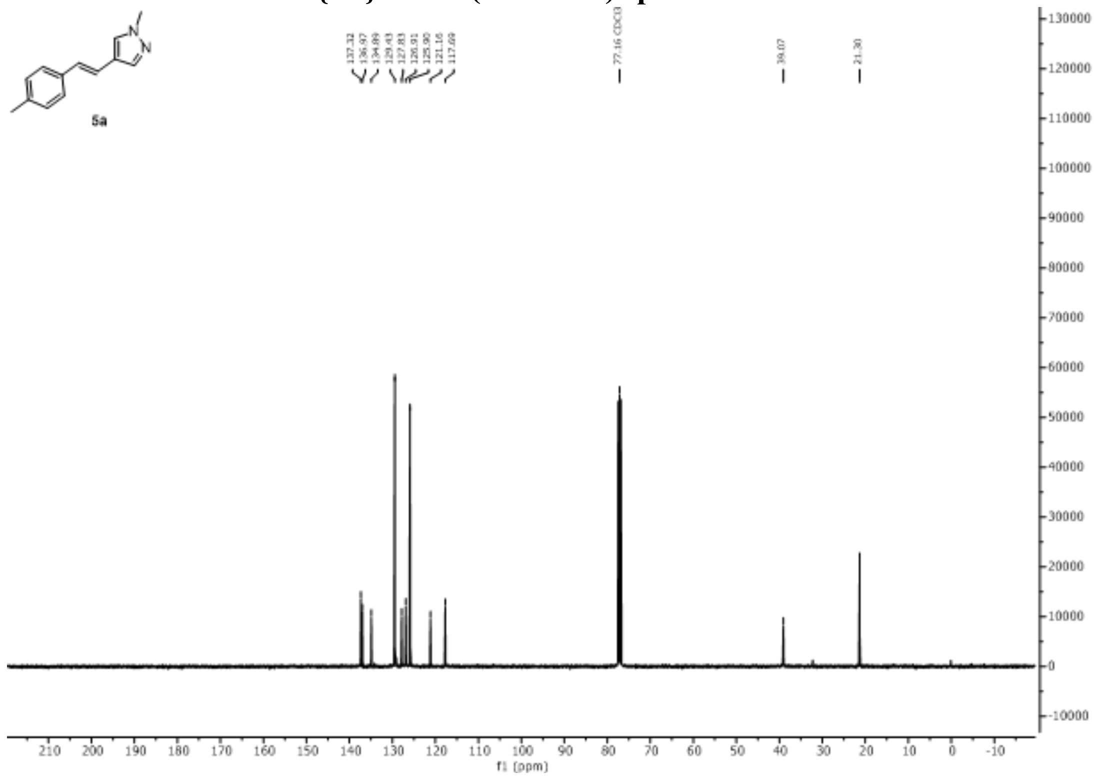

$^{13}C\{^1H\}$ NMR (101 MHz) spectrum for 5b

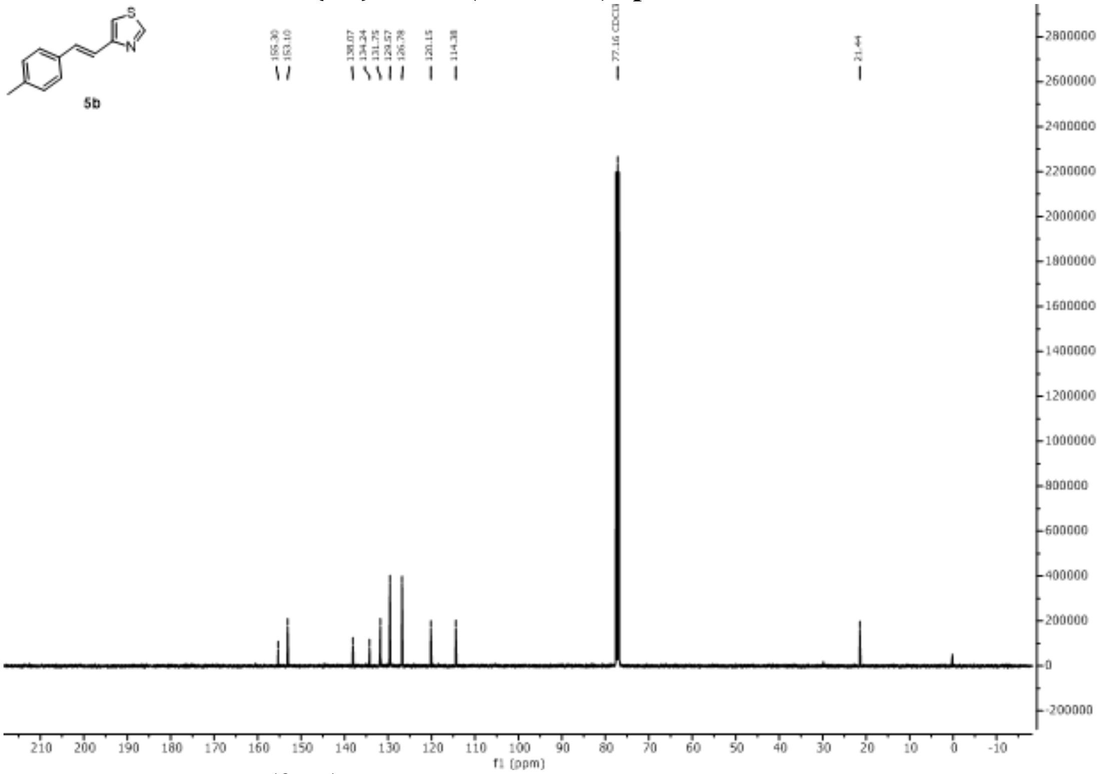

$^{13}C\{^1H\}$ NMR (101 MHz) spectrum for 5c

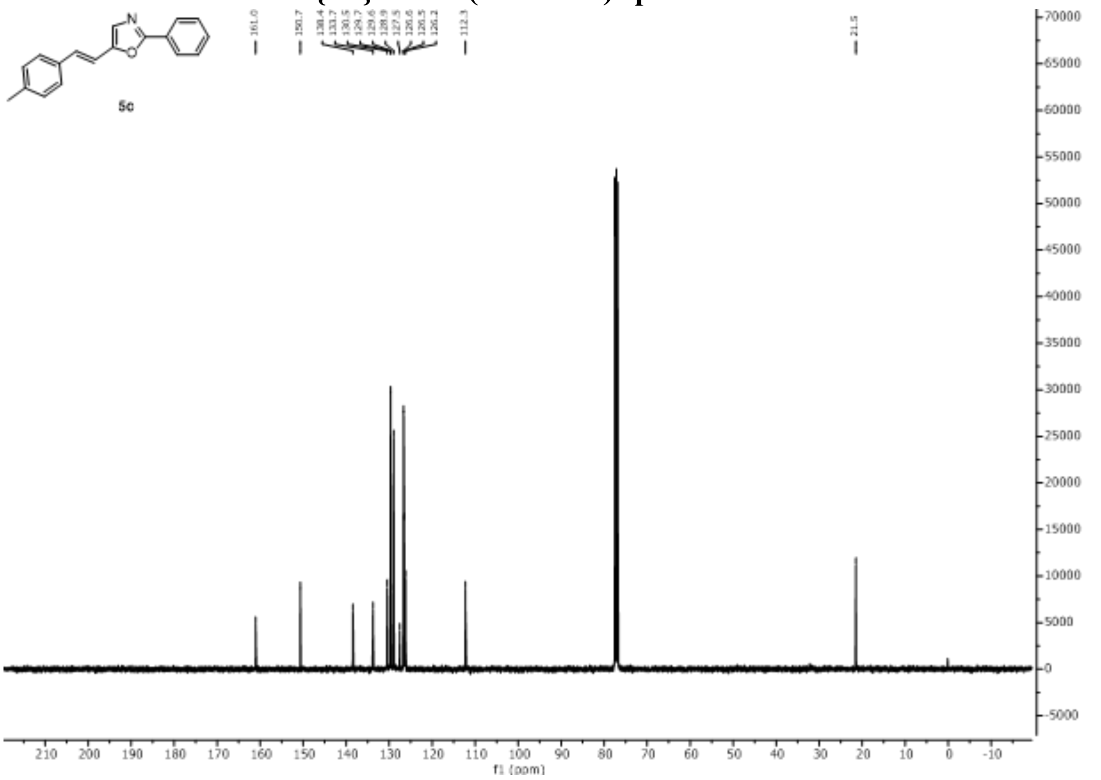

$^{13}C\{^1H\}$ NMR (101 MHz) spectrum for 5d

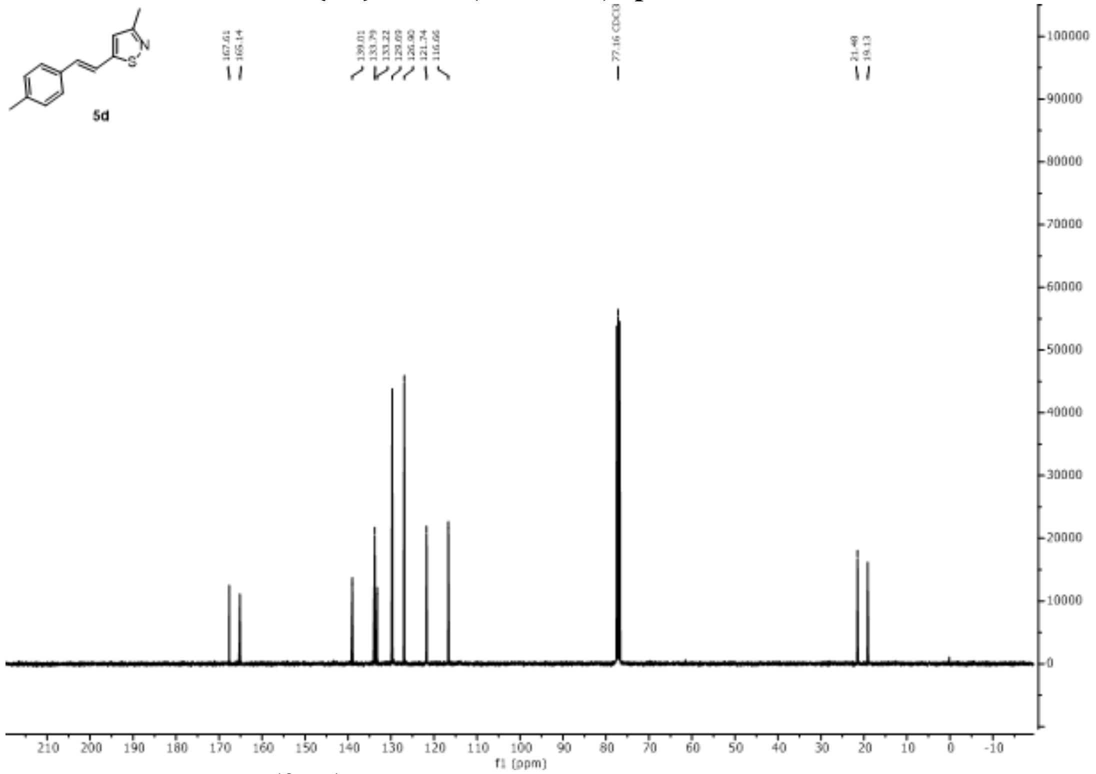

$^{13}C\{^1H\}$ NMR (101 MHz) spectrum for 5f

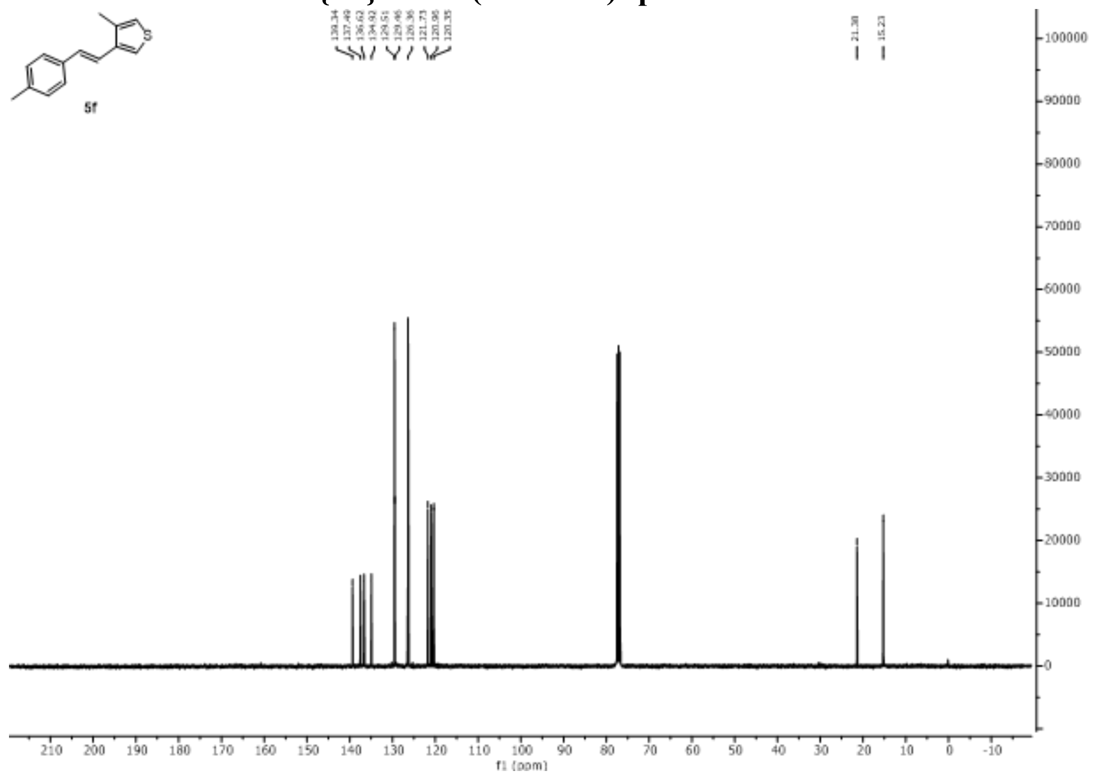

$^{13}C\{^{1}H\}$ NMR (101 MHz) spectrum for 5g

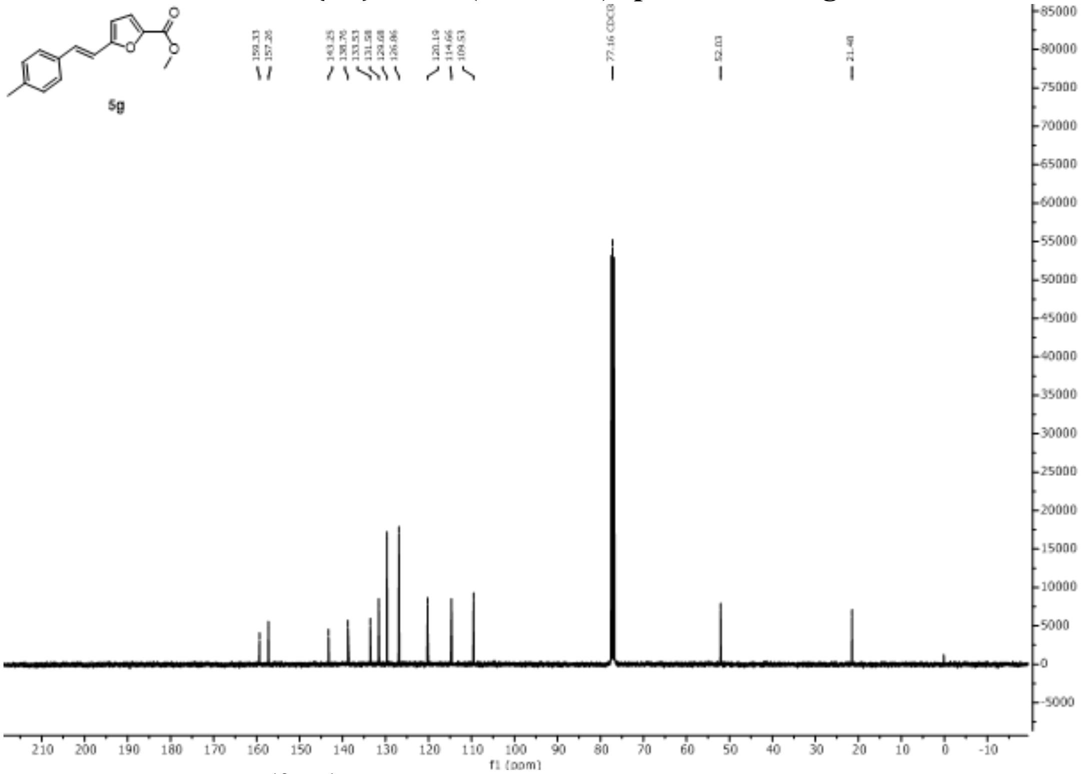

$^{13}C\{^{1}H\}$ NMR (101 MHz) spectrum for 6a

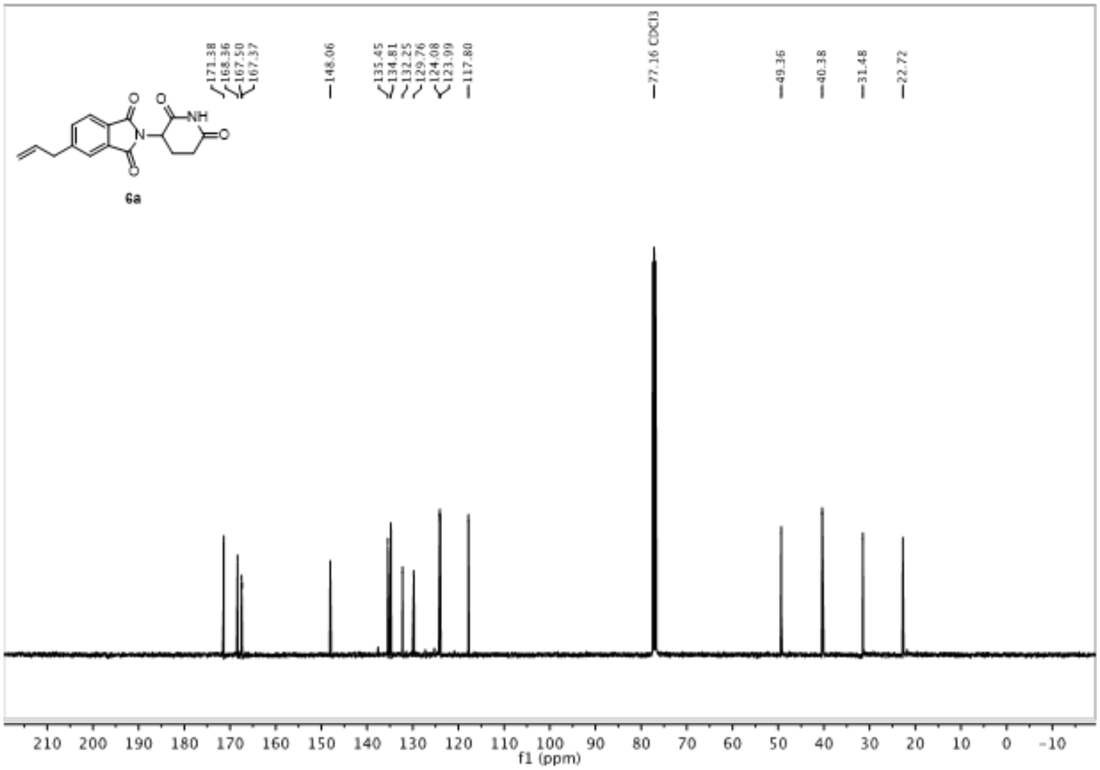

$^{13}C\{^1H\}$ NMR (101 MHz) spectrum for 6b

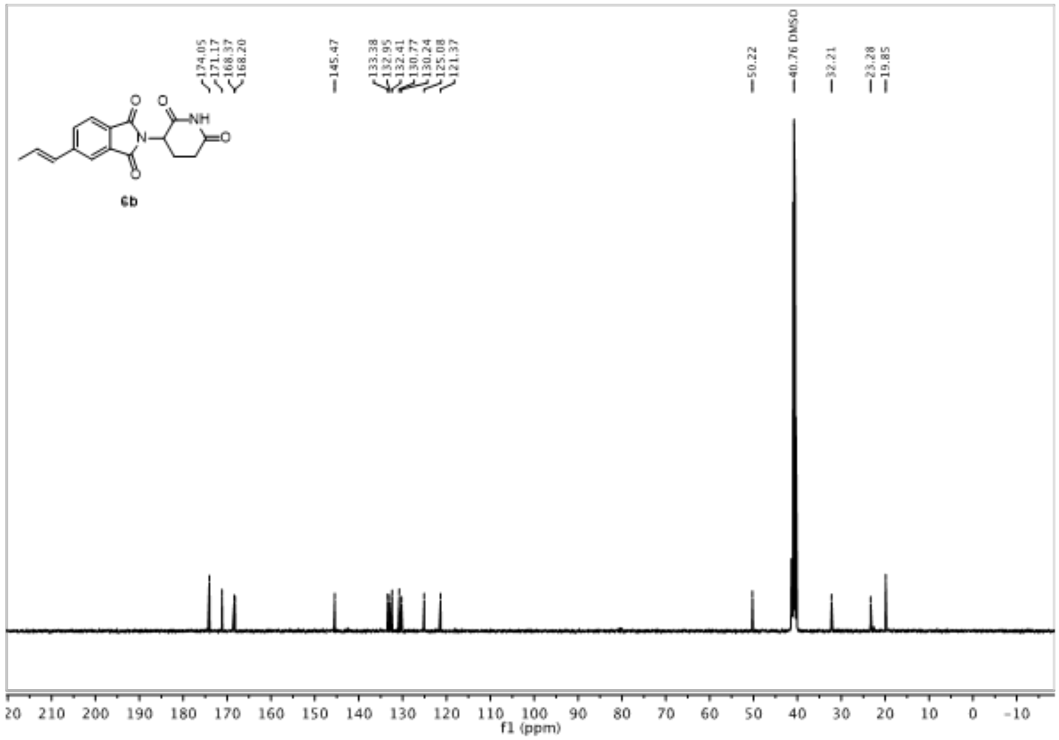

$^{13}C\{^1H\}$ NMR (101 MHz) spectrum for 6c

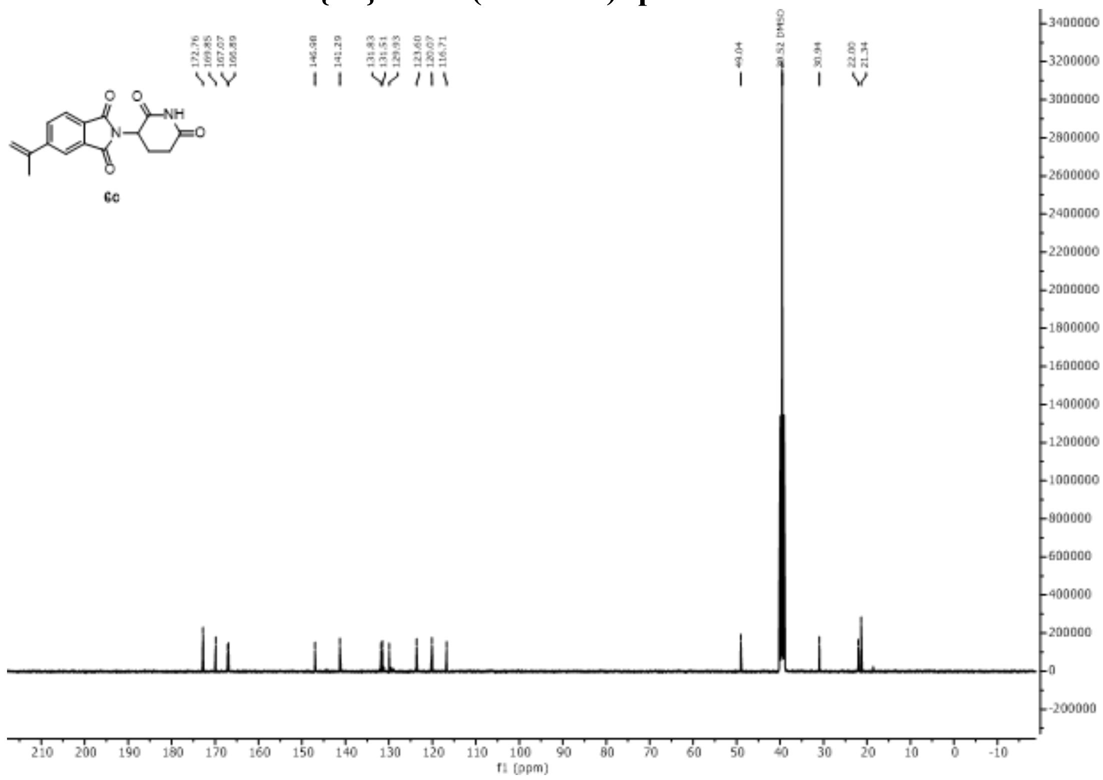

$^{13}C\{^{1}H\}$ NMR (101 MHz) spectrum for 6d

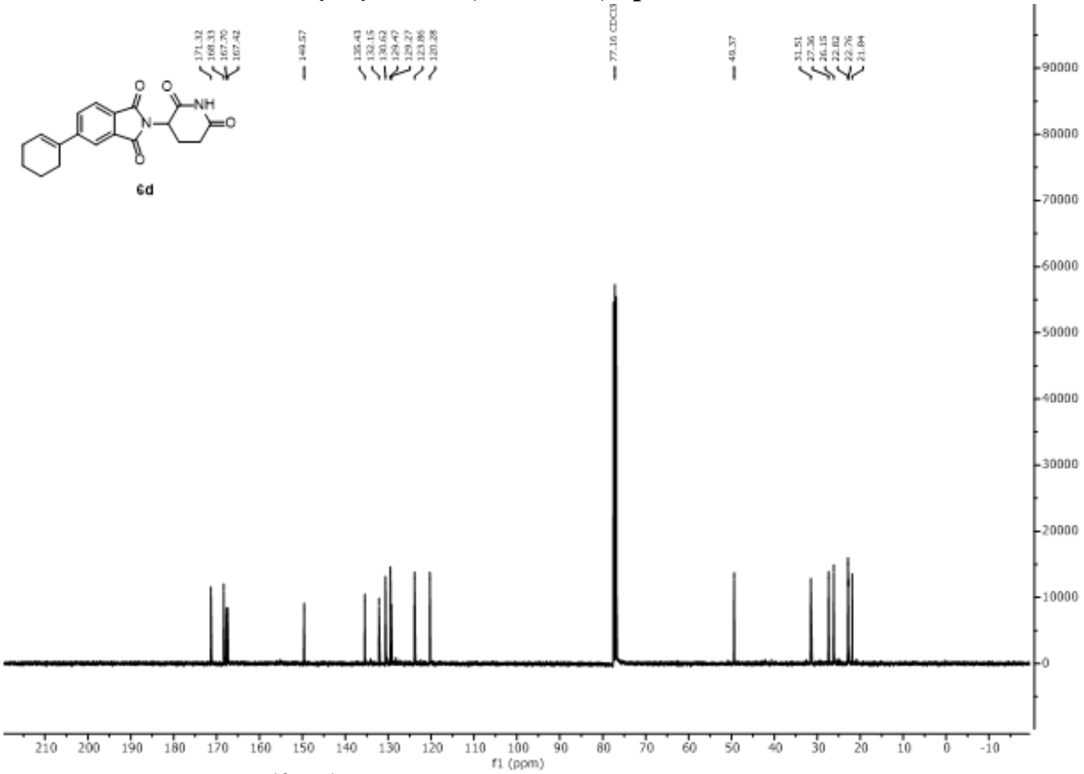

$^{13}C\{^{1}H\}$ NMR (101 MHz) spectrum for 6e

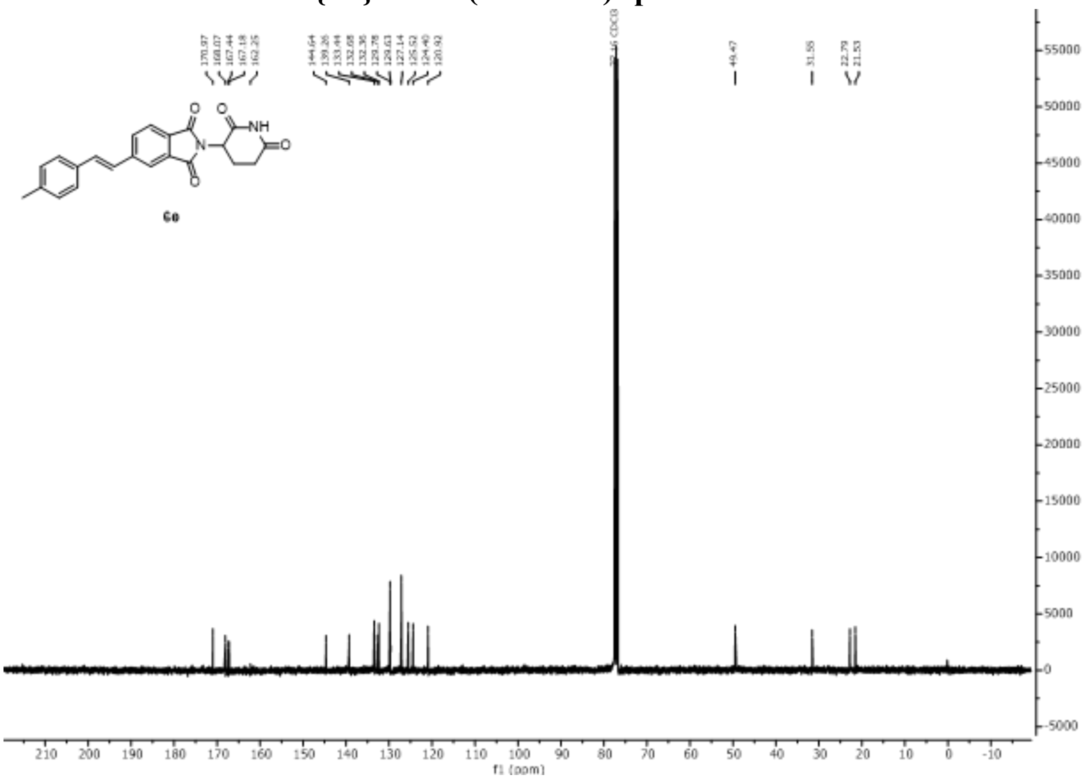

$^{13}C\{^{1}H\}$ NMR (101 MHz) spectrum for 6g

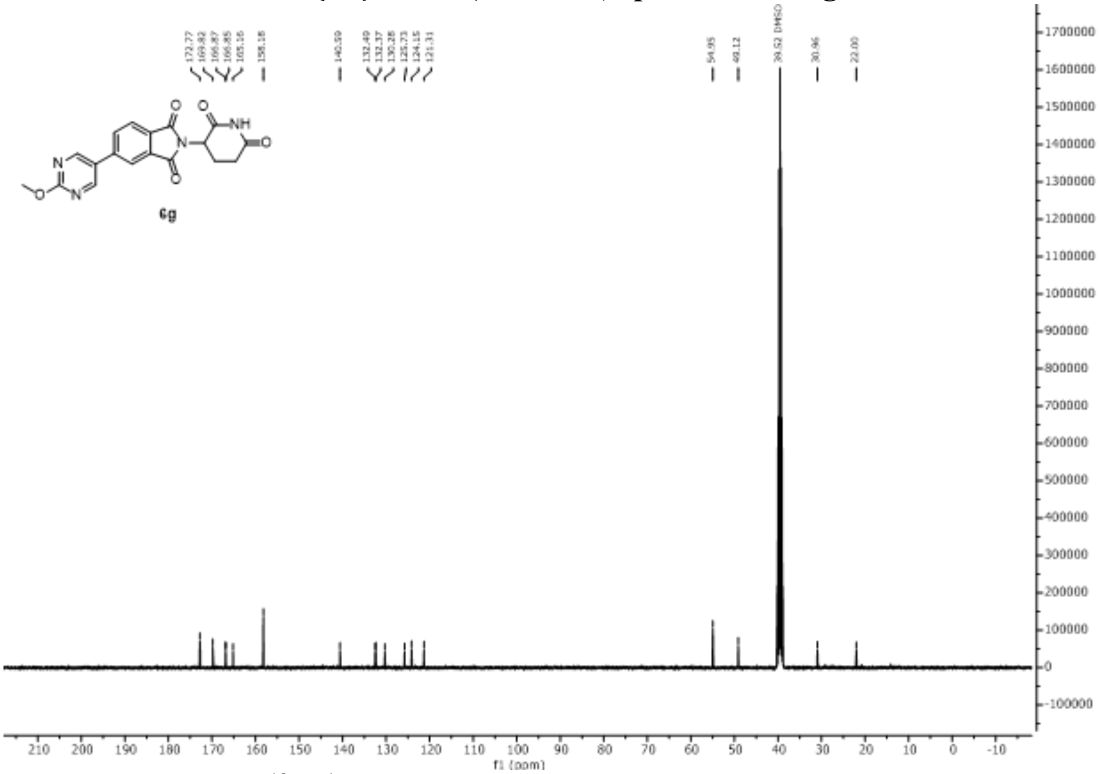

$^{13}C\{^{1}H\}$ NMR (101 MHz) spectrum for 6h

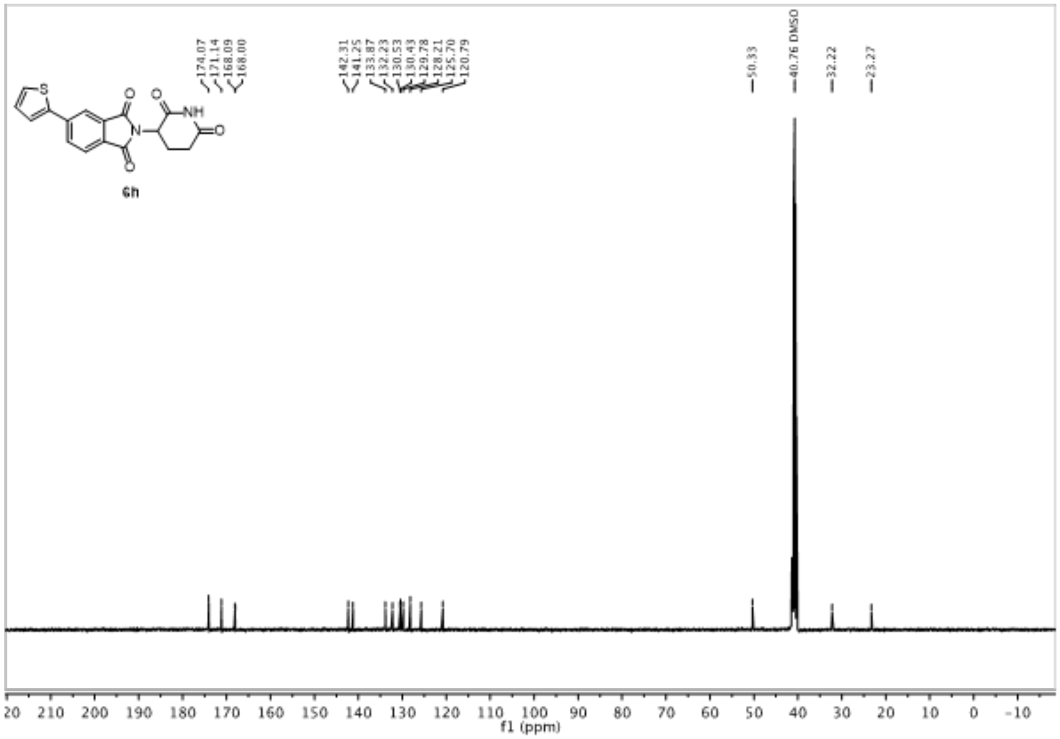

$^{13}C\{^1H\}$ NMR (101 MHz) spectrum for 6i

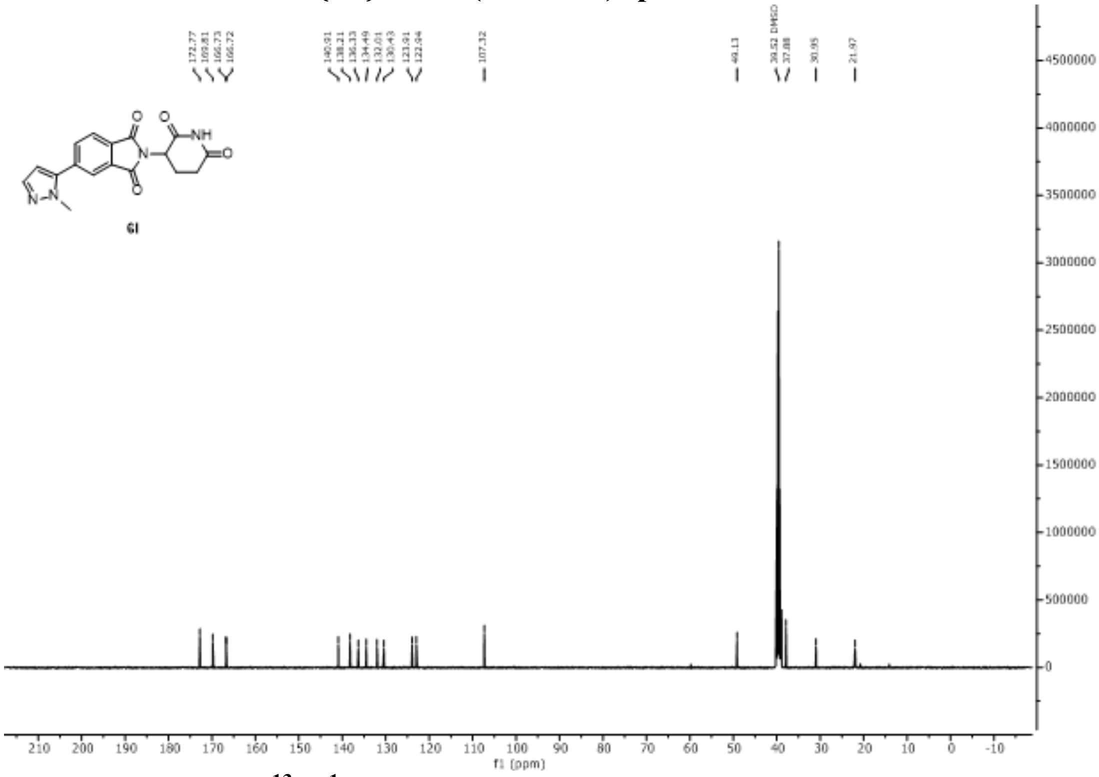

$^{13}C\{^1H\}$ NMR (101 MHz) spectrum for 6j

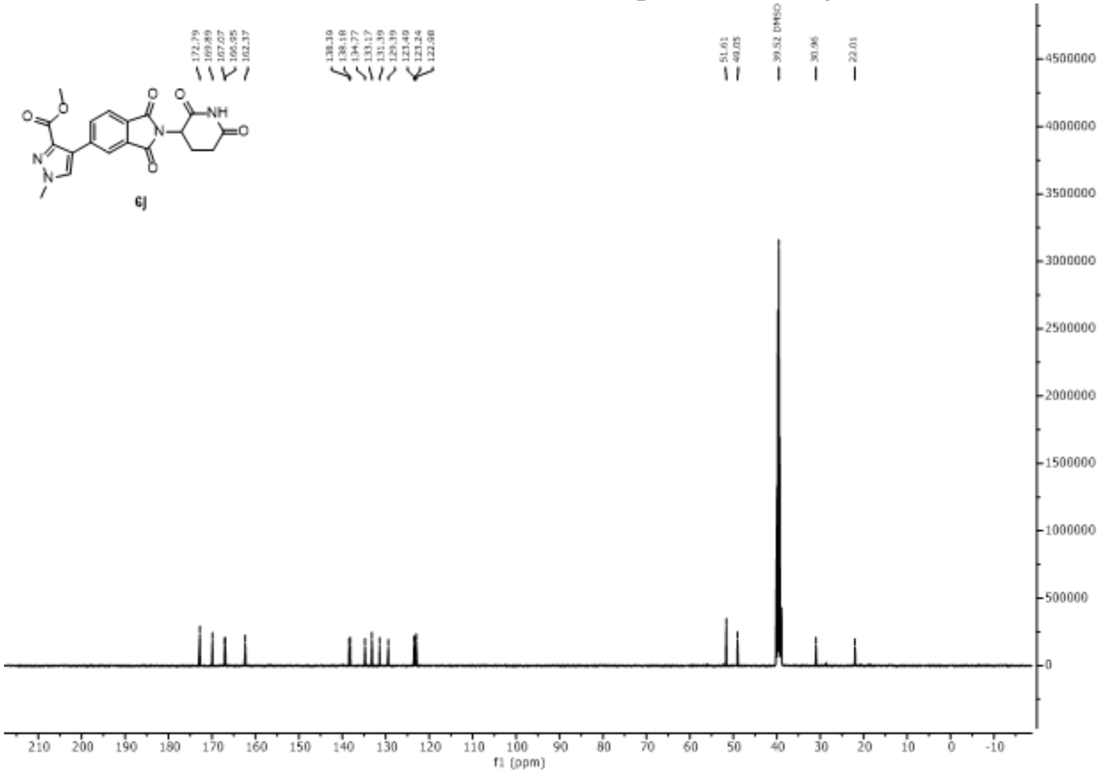

$^{13}C\{^{1}H\}$ NMR (101 MHz) spectrum for 6k

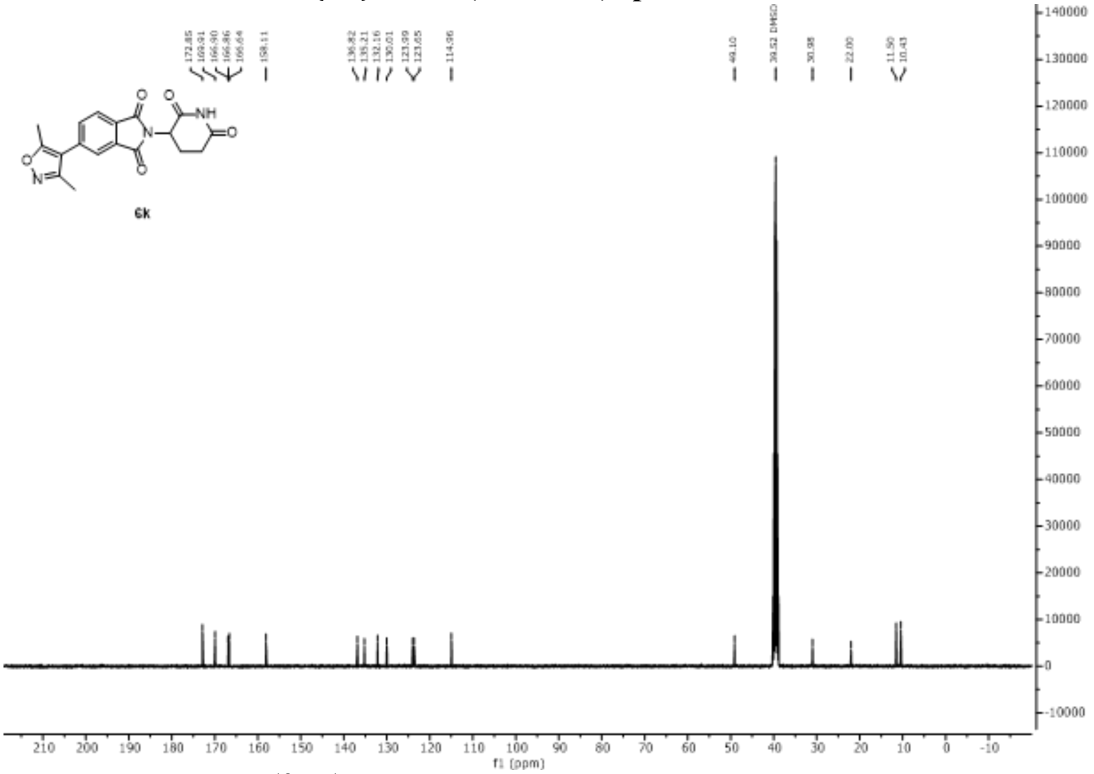

$^{13}C\{^{1}H\}$ NMR (101 MHz) spectrum for 6l

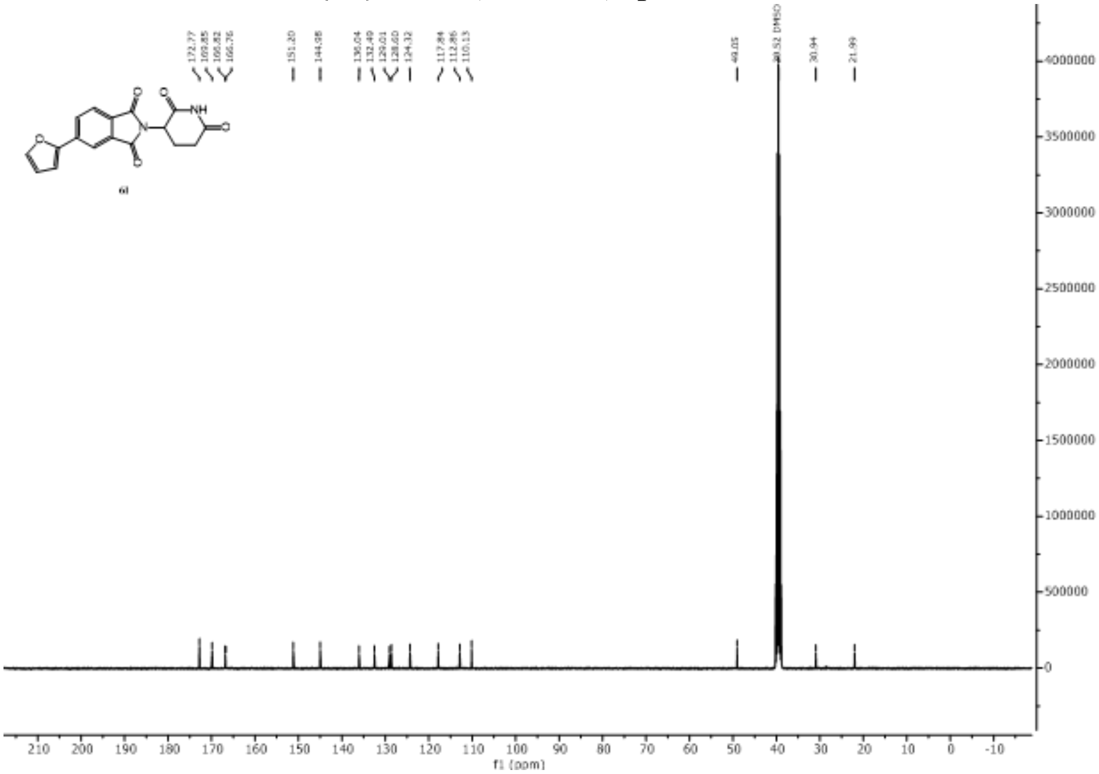

Chromatographic Data

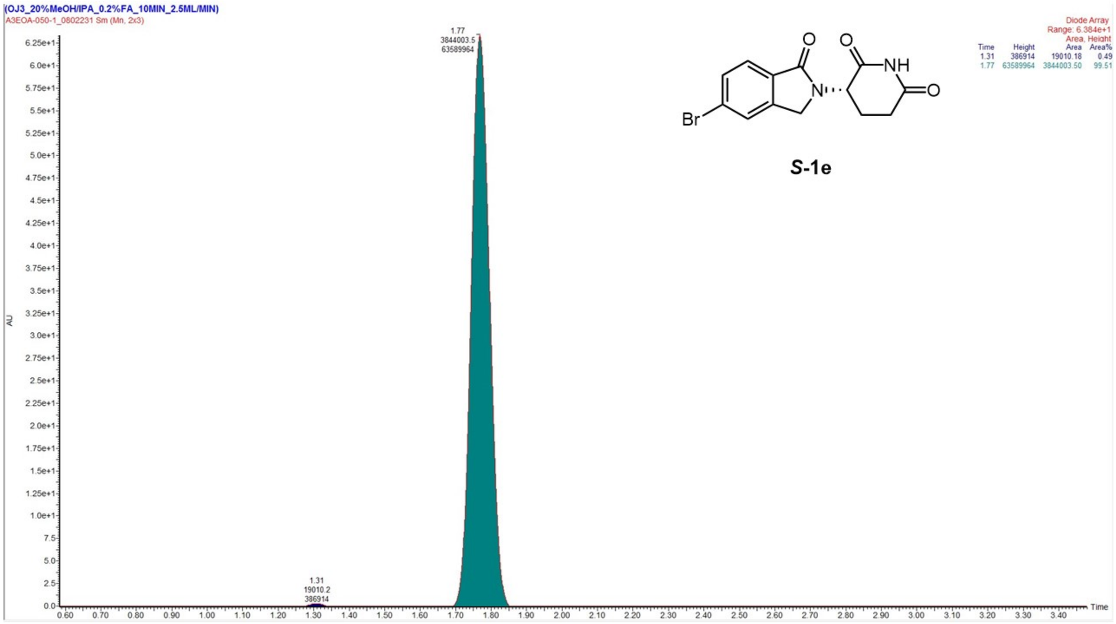

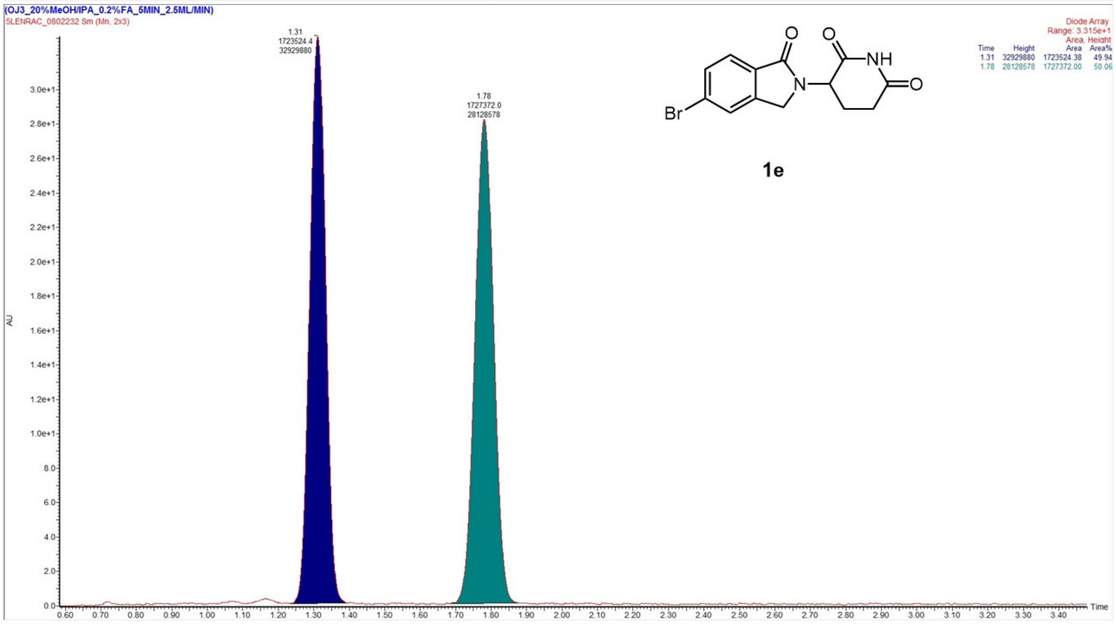

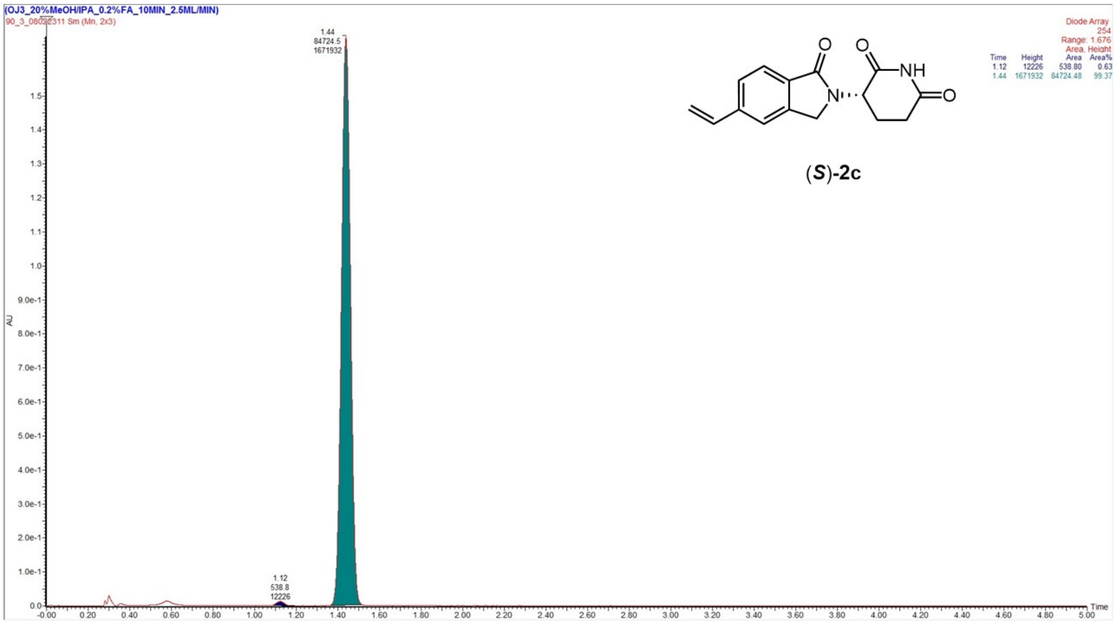

(OJ3_20%MeOH/IPA_0.2%FA_10MIN_2.5ML/MIN)
Diode Array
254
Range: 1.676
Area, Height
Time Height Area Area%
1.12 12226 538.80 0.63
1.44 1671932 84724.48 99.37
1.44
84724.5
1671932
1.12
538.8
12226
AU
Time
(S)-2c

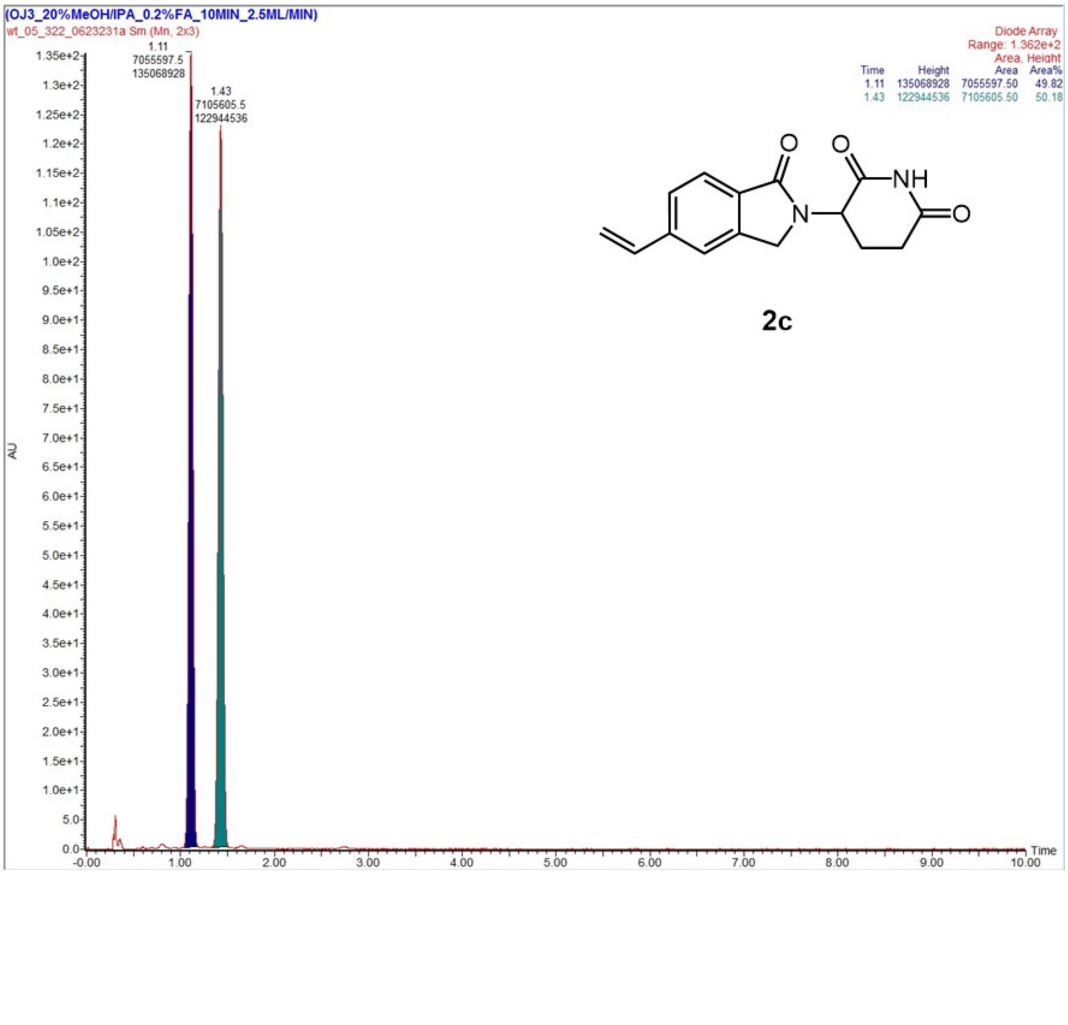

(OJ3_20%MeOH/IPA_0.2%FA_10MIN_2.5ML/MIN)
wt_05_322_0623231a Sm (Mn, 2x3)
Diode Array
Range: 1.362e+2
Area, Height
Time Height Area Area%
1.11 135068928 7055597.50 49.82
1.43 122944536 7105605.50 50.18
1.11
7055597.5
135068928
1.43
7105605.5
122944536
AU
Time
2c

References

1. Gaussian 16, Revision C.01, M. J. Frisch, G. W. Trucks, H. B. Schlegel, G. E. Scuseria, M. A. Robb, J. R. Cheeseman, G. Scalmani, V. Barone, G. A. Petersson, H. Nakatsuji, X. Li, M. Caricato, A. V. Marenich, J. Bloino, B. G. Janesko, R. Gomperts, B. Mennucci, H. P. Hratchian, J. V. Ortiz, A. F. Izmaylov, J. L. Sonnenberg, D. Williams-Young, F. Ding, F. Lipparini, F. Egidi, J. Goings, B. Peng, A. Petrone, T. Henderson, D. Ranasinghe, V. G. Zakrzewski, J. Gao, N. Rega, G. Zheng, W. Liang, M. Hada, M. Ehara, K. Toyota, R. Fukuda, J. Hasegawa, M. Ishida, T. Nakajima, Y. Honda, O. Kitao, H. Nakai, T. Vreven, K. Throssell, J. A. Montgomery, Jr., J. E. Peralta, F. Ogliaro, M. J. Bearpark, J. J. Heyd, E. N. Brothers, K. N. Kudin, V. N. Staroverov, T. A. Keith, R. Kobayashi, J. Normand, K. Raghavachari, A. P. Rendell, J. C. Burant, S. S. Iyengar, J. Tomasi, M. Cossi, J. M. Millam, M. Klene, C. Adamo, R. Cammi, J. W. Ochterski, R. L. Martin, K. Morokuma, O. Farkas, J. B. Foresman, and D. J. Fox, Gaussian, Inc., Wallingford CT, 2016.
